# Pulse Dipolar Electron Paramagnetic Resonance Spectroscopy Distance Measurements at Low Nanomolar Concentrations: The Cu^II^-Trityl Case

**DOI:** 10.1021/acs.jpclett.3c03311

**Published:** 2024-01-31

**Authors:** Katrin Ackermann, Caspar A. Heubach, Olav Schiemann, Bela E. Bode

**Affiliations:** † EaStCHEM School of Chemistry and Biomedical Sciences Research Complex, Centre of Magnetic Resonance, University of St Andrews, North Haugh, St Andrews KY16 9ST, U.K.; ‡ Clausius-Institute of Physical and Theoretical Chemistry, University of Bonn, Wegelerstrasse 12, 53115 Bonn, Germany

## Abstract

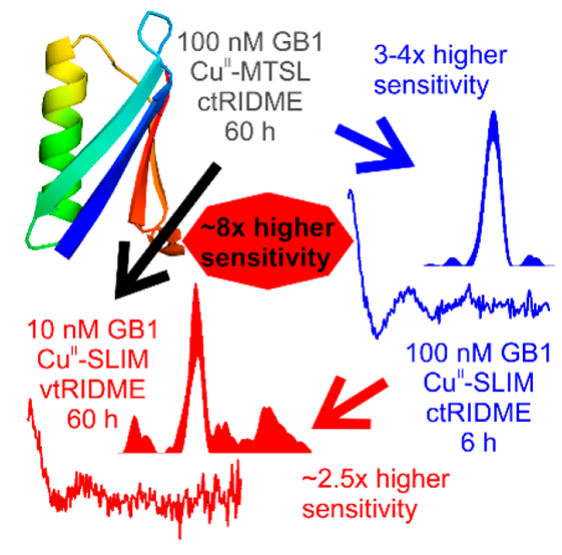

Recent sensitivity enhancements in pulse dipolar electron paramagnetic resonance spectroscopy (PDS) have afforded distance measurements at submicromolar spin concentrations. This development opens the path for new science as more biomolecular systems can be investigated at their respective physiological concentrations. Here, we demonstrate that the combination of orthogonal spin-labeling using Cu^II^ ions and trityl yields a >3-fold increase in sensitivity compared to that of the established Cu^II^-nitroxide labeling strategy. Application of the recently developed variable-time relaxation-induced dipolar modulation enhancement (RIDME) method yields a further ∼2.5-fold increase compared to the commonly used constant-time RIDME. This overall increase in sensitivity of almost an order of magnitude makes distance measurements in the range of 3 nm with protein concentrations as low as 10 nM feasible, >2 times lower than the previously reported concentration. We expect that experiments at single-digit nanomolar concentrations are imminent, which have the potential to transform biological PDS applications.

Studying the structure–function relationship of proteins and their complexes under physiological conditions is a major task for our understanding of the biomolecular mechanisms underpinning health and disease. However, the high concentration sensitivity required to meet physiologically relevant conditions pushes many biophysical methods, especially magnetic resonance techniques, to or even beyond their sensitivity limits. In this Letter, we demonstrate that distance measurements based on pulse dipolar electron paramagnetic resonance spectroscopy (PDS) can be performed at the required low nanomolar protein concentrations. This becomes possible by combining recent developments in pulse sequences with new labeling techniques.

Recent developments in electron paramagnetic resonance (EPR) spectroscopy have firmly established PDS in the toolbox for structural biology, due to its accuracy, reproducibility, and concentration sensitivity, and by providing access to full conformational ensembles of biomolecules.^1−6^ PDS methods such as pulsed electron–electron double resonance (PELDOR or DEER),^7−9^ relaxation-induced dipolar modulation enhancement (RIDME),^10−14^ and double quantum coherence (DQC)^1,3,15^ provide distance distributions in the nanometer range (from ∼2 to ∼16 nm)^16−18^ and have become important tools for studying complex biomolecular systems.^19,20^

PDS requires the presence of paramagnetic centers that are dipolarly coupled; distance distributions can then be derived from the dipolar coupling frequencies. The beauty of this technique is that PDS is exquisitely and exclusively sensitive to these paramagnetic centers, such that the size, shape, and complexity of the overall biomolecular assembly are not considered limiting factors, allowing studies of proteins, nucleic acids, and their complexes *in vitro* and in cell.^2,18,21−32^

In most cases, paramagnetic centers are introduced via site-directed spin-labeling (SDSL) of specific residues, usually employing stable organic radicals such as nitroxides^1,33−35^ or trityls^3,23,36^ or paramagnetic metal ions such as Gd^III^,^21,34,36−39^ Mn^II^,^40^ or Cu^II^.^41−44^ In most cases, SDSL involves stable radicals introduced via cysteine-specific chemistry, where the label-specific length of the linker contributes degrees of freedom to the dynamics of the protein, which artificially broadens distance distributions or allows only distinct label conformations that could make interpretation of distance distributions ambiguous.^45,46^ Additionally, different spin-labels may lead to different degrees of perturbation of the native structure due to their size and interactions with protein residues, e.g., trityl labels. Another approach uses Cu^II^ complexed with nitrilotriacetic acid (CuNTA) coordinated to a site-specifically introduced double-histidine (dHis) motif (dHis-CuNTA), posing more stringent requirements on the labeling site but yielding very narrow distance distributions due to the rigidity of this labeled side chain.^35,42,47−50^ CuNTA increases specificity to dHis sites compared to free Cu^II^ in solution, which has a stronger propensity for unspecific binding.^42,47,51^ Depending on the secondary structural elements (α-helix or β-sheet), dissociation constants (*K*_d_) were determined to be on the order of 10^–5^ to 10^–7^ under EPR conditions.^49,52^ An advantage of using spectroscopically orthogonal labels is that binding sites can be saturated by an excess of CuNTA without overlap with the detected signal.^52^ Furthermore, the dHis-CuNTA labeling has been shown to be robust against competing ligands and to retain its high affinity binding over a wide pH range, thus demonstrating biologically relevant compatibility.^50^ While a majority of the benchmarking studies on dHis-CuNTA have been performed on different constructs of a model protein (GB1, *vide infra*), this labeling approach has also been applied to a variety of more complex biological systems.^30,45,53^ Despite its vulnerability to reduction, CuNTA has been used for in-cell PDS.^54^

Sensitivity is crucially important for the PDS studies. Over the past decade, the research questions investigated with PDS have involved ever larger biomolecular complexes.^19^ It is conceivable that these are concentration-limited, due to either poor solubility or a low expression yield. Studying the dispersed state of proteins undergoing liquid–liquid phase separation at micromolar concentrations^55^ requires submicromolar sensitivities. Furthermore, physiologically relevant conditions normally require high-sensitivity techniques due to the generally low concentrations of most biomolecules *in cellulo* or *in vivo*. Recent benchmarking studies have demonstrated that PDS allows measurements at nanomolar spin concentrations *in vitro* and in cell. In one study, spectroscopically orthogonal labeling using dHis-CuNTA and the nitroxide MTSL in combination with RIDME measurements yielded reliable distance information in the short-to-medium distance range down to a protein concentration of 100 nM *in vitro*,^6^ and similar concentration limits were reported for *in vitro* nitroxide–nitroxide and in-cell Gd^III^–Gd^III^ PELDOR measurements.^2^ Recent studies reported DQC-based distance measurements at protein concentrations of 25 nM (short distance range) and 45 nM (long distance range) doubly labeled with MTSL^1^ and oxSLIM, respectively.^3^ At sufficiently low temperature and spin concentration, the electron spin dephasing time becomes independent of concentration,^56^ and the achievable distance range is largely limited by deuteration levels of the sample.^4,57^

With the commonly used large volume resonators and rectangular pulses, one limiting factor for the sensitivity of nitroxides is the excitation bandwidth; here, labels with a narrow spectral line width such as trityls could entail a 3–4-fold increase in sensitivity by allowing excitation of the full spectrum. In this work, we benchmarked concentration sensitivities for spectroscopically orthogonal labeling using dHis-CuNTA^47^ in combination with the trityl-based label SLIM^23^ at standard Q-band (34 GHz) settings in a model protein. We hypothesized that by exchanging the nitroxide MTSL^6^ with the trityl SLIM, and by employing the recently introduced variable-time RIDME experiment,^13^ distance measurements in the low-nanomolar concentration regime should be feasible.

To enable direct comparison to some of the previous benchmarking studies,^5,6^ dHis-CuNTA and SLIM were grafted onto the *Streptococcus* sp. group G protein G, B1 domain (GB1), an established model protein extensively used for EPR studies.^1,5,6,36,42,47,49,50,52,58^ Here, the GB1 construct I6C/K28H/Q32H was used,^6,52^ bearing the dHis motif for coordination to CuNTA in an α-helix and a cysteine residue for SLIM labeling in a β-sheet (Figure 1).

**Figure 1:**
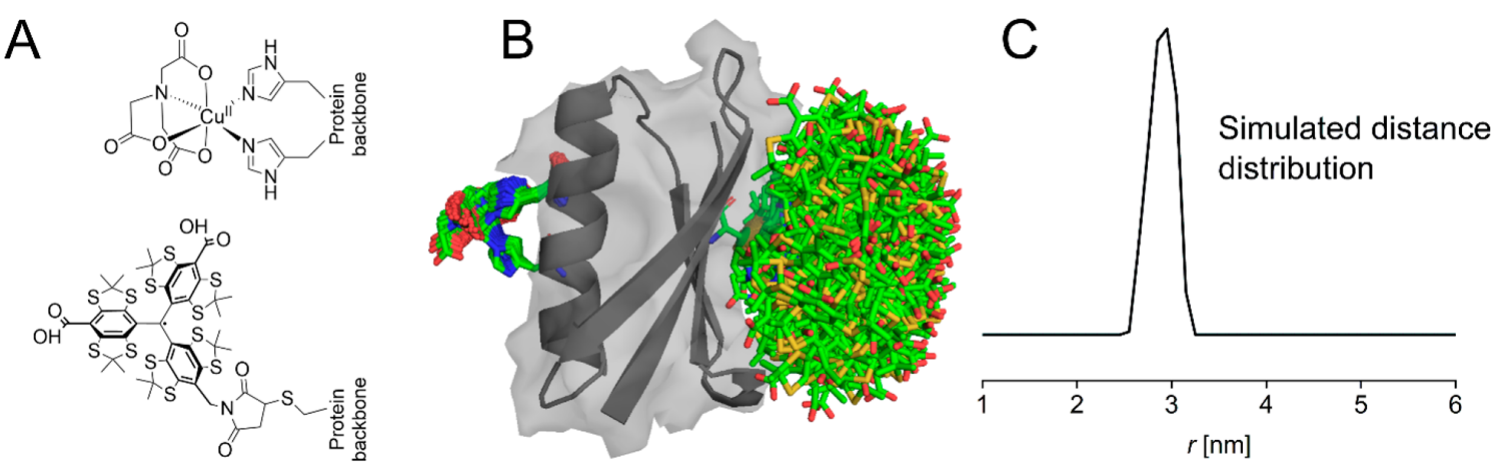
Predicted spatial distribution of the SLIM label and dHis-CuNTA in the GB1 construct I6SLIM/K28H/Q32H, based on the crystal structure of Protein Data Bank entry 4WH4. (A) Structural drawings for CuNTA coordinated to two histidine residues (top) and the SLIM label (bottom). (B) Visualization of the labeled GB1 construct, with modeled dHis-CuNTA and SLIM rotamers^59^ shown as sticks. (C) Simulated distance distribution obtained from Colab running mtsslWizard for bipedal labels.^35,59,60^

GB1 I6C/K28H/Q32H was expressed and purified as described previously^52^ and labeled with SLIM and CuNTA according to established protocols (for more details, see the Supporting Information).^23,45,52^ Quantitative labeling with SLIM could be achieved as demonstrated by sample characterization using continuous wave (CW) EPR spectroscopy (Figure S1), electrospray ionization (ESI) mass spectrometry (Figure S2), and ultraviolet–visible (UV–vis) spectroscopy (Figure S3) for concentration determination and estimation of labeling efficiency.

Initially, temperature optimization in the range of 30–70 K was performed by generating a temperature-dependent RIDME sensitivity profile (Figure S4 and Tables S2 and S3).^52^ An optimum temperature of 40 K was determined for CuNTA-SLIM RIDME measurements, in agreement with a recent study.^45^ Thus, compared to the CuNTA-nitroxide RIDME measured at 30 K,^52^ here the unfavorable change in thermal polarization resulting from a 10 K increase in temperature was outweighed by a faster repetition rate with a higher temperature. With the repetition rate of the trityl (at 40 K in this study) and of the nitroxide (at 30 K)^6,52^ being very similar, an overall increase in the sensitivity of the CuNTA-SLIM RIDME measurement will therefore be due to the excitation of the full spectral width of the trityl. Assuming that only approximately one-quarter to one-third of the nitroxide spectrum has been excited in previous CuNTA-nitroxide RIDME studies,^6,52^ we hypothesized that an increase in sensitivity by a factor of 3–4 could be expected in an ideal case.

To test this hypothesis, a dilution series was set up, ranging from 500 nM to 10 nM protein, with the CuNTA concentration in each sample calculated to yield approximately 90% binding (Table S1).^52^ Standard constant-time (ct) RIDME measurements were performed with identical experimental parameters for each sample, with the exception of the averaging times (and, thus, the number of scans) varying between 2 h for the 500 nM sample to ∼48 h for the 10 nM sample (Figure 2 and Figure S5).

**Figure 2:**
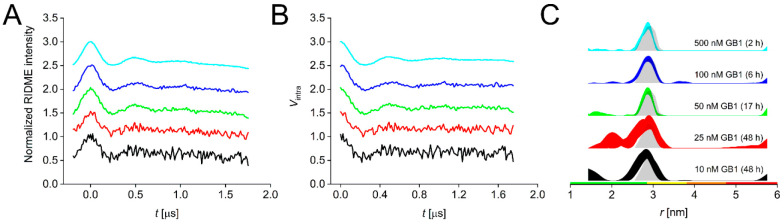
ctRIDME data for the GB1 I6SLIM/K28H/Q32H dilution series. (A) Stacked raw and (B) background-corrected RIDME traces for the 500 nM (cyan), 100 nM (blue), 50 nM (green), 25 nM (red), and 10 nM (black) samples. (C) Corresponding distance distributions given as 95% confidence estimates (±2σ) with 50% noise added for error estimation during statistical analysis and simulated distance distributions shown as gray shaded areas. Color bars represent reliability ranges: green for shape reliable, yellow for mean and width reliable, orange for mean reliable, and red for no quantification possible.

ctRIDME data were processed using a Tikhonov regularization procedure within DeerAnalysis2022^61^ as described previously,^6^ and the obtained distance distributions for all samples were in excellent agreement with the simulated distribution. It should be noted that our in-house RIDME processing protocol follows a very conservative approach, potentially overestimating the uncertainty in the resulting distance distribution (for details, see the Supporting Information). While we obtained high-confidence distance distributions down to 50 nM protein, lower concentrations led to significantly increased uncertainties, including broader confidence bands, and such data should be interpreted only with great care.^4^ Thus, concentrations of <50 nM remain a challenge for ctRIDME even when using CuNTA-SLIM label pairs, and higher-quality data with lower uncertainties than seen for 10 and 25 nM will be required to, e.g., determine whether multiple distance populations are present, as observed previously.^5^ This is in line with data obtained from alternative deep neural network processing (DEERNet^62^ with a RIDME background model^63^ and ConsensusDeerAnalyzer2.0 within DeerAnalysis2022), which returned confirmative results for higher protein concentrations (Table S5) but failed for concentrations of ≤50 nM.

Data were also evaluated on the basis of their respective sensitivity values. Briefly, we define sensitivity as the ratio of modulation depth divided by the root-mean-square experimental noise; this modulation-to-noise ratio is further normalized as described previously,^5,6,52^ thereby allowing a direct comparison of sensitivities obtained in different studies (see the Supporting Information for details on sensitivity analysis and tabulated values). Here, of particular interest was the direct sensitivity comparison to CuNTA-nitroxide ctRIDME measurements on the same GB1 construct that was used in this study (GB1 I6R1/K28H/Q32H, with R1 being the name of the side chain obtained from MTSL labeling of a cysteine residue) at protein concentrations of 50 and 100 nM.^6^ As detailed above, the ability to excite the full width of the trityl EPR spectrum should lead to an increase in sensitivity of a factor of approximately 3–4 in an ideal case. Indeed, our data revealed an improvement of a factor of >3 (Table S4), and we were able to measure a 50 nM GB1 sample overnight (17 h) instead of averaging for ∼60 h;^6^ we would refrain from using the data obtained at lower concentrations using ctRIDME due to the poor modulation-to-noise ratios achieved. The sensitivity comparison also identifies the 25 nM sample as an outlier of the series, with an extrapolated sensitivity of around half of the expected value, while the 100, 50, and 10 nM samples give values in good agreement with extrapolated values (see Table S4, column “S_t_ at 1 μM extrapolated”).

In the next step, we investigated whether the recently reported variable-time RIDME experiment (vtRIDME)^13^ would enable a further sensitivity increase particularly valuable at these low concentrations. To ensure reproducibility and minimize errors from sample positioning and experiment optimization, four experiments were recorded on the samples of the dilution series: a ctRIDME similar to the measurement described above, a vtRIDME with otherwise identical parameters, and corresponding reference traces for both the ctRIDME and the vtRIDME to enable deconvolution of the data (division by the reference trace).^52^ Nondeconvoluted vtRIDME data are shown in Figure 3, and for a complete set of the ct/vtRIDME data, see Figure S6.

**Figure 3:**
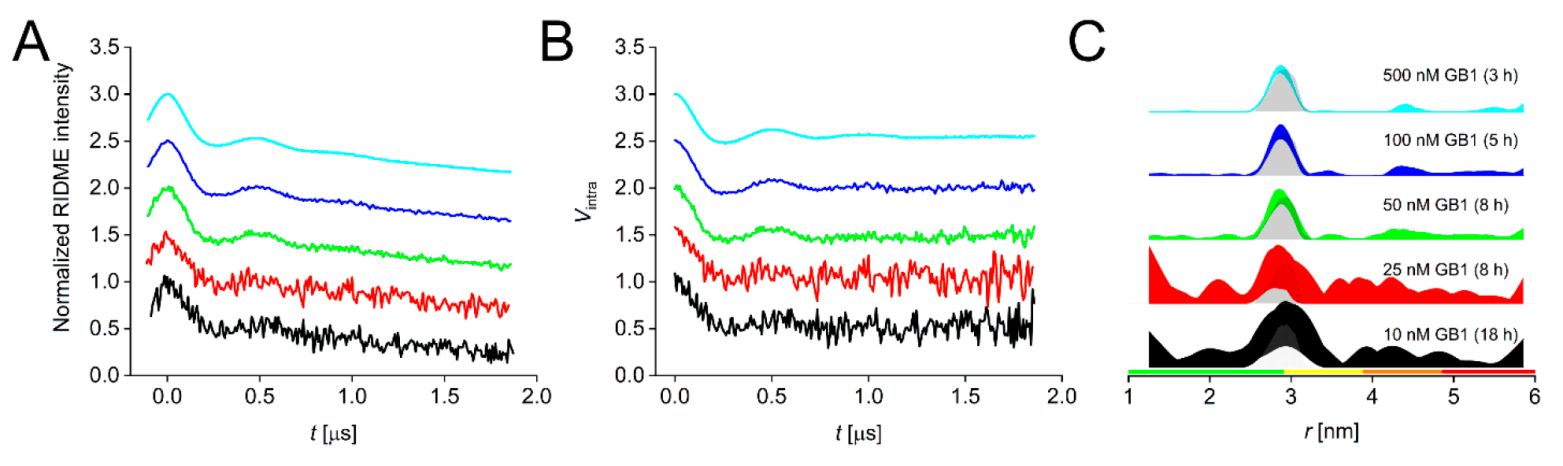
vtRIDME data for the GB1 I6SLIM/K28H/Q32H dilution series. (A) Stacked raw and (B) background-corrected RIDME traces for the 500 nM (cyan), 100 nM (blue), 50 nM (green), 25 nM (red), and 10 nM (black) samples. (C) Corresponding distance distributions given as 95% confidence estimates (±2σ) with 50% noise added for error estimation during statistical analysis and simulated distance distributions shown as gray shaded areas. Color bars represent reliability ranges: green for shape reliable, yellow for mean and width reliable, orange for mean reliable, and red for no quantification possible.

For the sake of convenience, the four different experiments have been combined into a single pulse program. For samples with a protein concentration of <100 nM, recording the reference traces became unfeasible due to the required extended averaging times and was therefore done only for concentrations of ≥100 nM. As observed previously,^13,35^ modulation depths were slightly higher for the vtRIDME than for the ctRIDME. Deconvolution reduced the observed modulation depths for both ct- and vtRIDME measurements as expected. vtRIDME data were processed in the same way as described for the ctRIDME. Notably, here the additional processing with ConsensusDeerAnalyzer2.0 failed only for the lowest concentration (10 nM GB1) (Table S6), and a high-confidence distance distribution was obtained at 50 nM in 8 h. Sensitivity analysis revealed an approximately 2.5-fold increase in sensitivity for vtRIDME compared to ctRIDME (Table S4), which is in excellent agreement with recently published data.^13^ While for the 50 nM vtRIDME a modulation-to-noise ratio of >20 was achieved with an averaging time of 8 h, the 25 and 10 nM samples yielded ratios of <10 with averaging times of 8 and 18 h, respectively; data of this quality should not be used to interpret distance distributions.^4^ Thus, together with the change from CuNTA-nitroxide to CuNTA-SLIM labeling, an overall increase in sensitivity of almost an order of magnitude (∼8-fold) compared to the previous study^6^ has been achieved.

Finally, to demonstrate that 10 nM measurements can be recorded at sufficient quality, we averaged a vtRIDME experiment of the 10 nM GB1 sample for approximately 60 h, our internal maximum averaging time used for previous sensitivity benchmarking,^6^ which yielded clearly visible oscillations, and a modulation-to-noise ratio of 12.5 (Figure 4). While this is below the recent recommendation of a modulation-to-noise ratio of at least 20, these data are still of sufficient quality to provide useful structural restraints.^4^ An attempt to further decrease the protein concentration to 5 nM for a vtRIDME measurement turned out to be beyond our current sensitivity limit and was considered unsuitable for processing (Figure S7).

**Figure 4:**
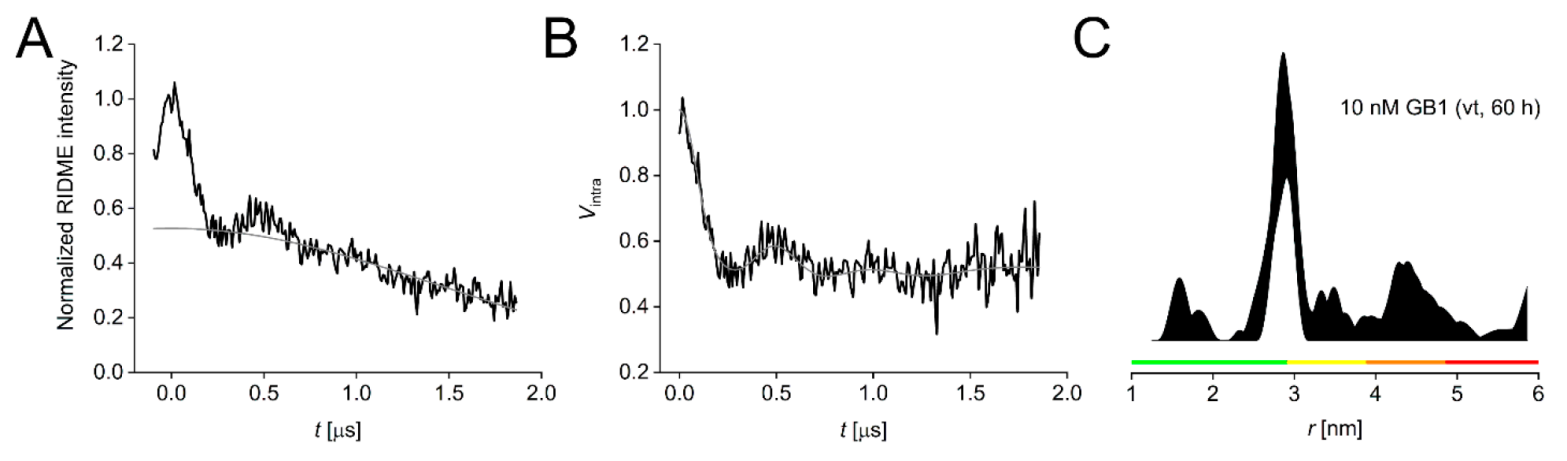
vtRIDME data for the 10 nM GB1 I6SLIM/K28H/Q32H sample averaged over 60 h. (A) Raw vtRIDME trace (black) with background (gray). (B) Background-corrected vtRIDME trace (black) and fit (gray). (C) Corresponding distance distribution given as the 95% confidence estimate (±2σ) with 50% noise added for error estimation during statistical analysis. Color bars represent reliability ranges: green for shape reliable, yellow for mean and width reliable, orange for mean reliable, and red for no quantification possible.

In summary, we show that combining CuNTA-SLIM spin-labeling with vtRIDME can significantly enhance PDS sensitivity by almost an order of magnitude compared to established procedures (i.e., ctRIDME measurement of CuNTA-nitroxide-labeled protein), reaching a concentration limit of 10 nM in the distance range of 3 nm. It is expected that longer distances and broader or more complex distributions will require longer dipolar evolution times or a better modulation-to-noise ratio. This would likely demand concentrations higher than those achieved here. Together with recent advances in hardware,^1,64^ we predict that single-digit nanomolar PDS distance measurements will soon be within reach in favorable cases. This will enable new science, particularly with respect to concentration-limited systems.

## Data Availability

The research data supporting this publication will be accessible at 10.17630/4a648038-3e79-4eaf-a185-f6721e4060fa.^65^

## Supporting Information Available

Protein expression, purification, and spin-labeling; continuous wave (CW) EPR, mass spectrometry, and UV–vis data; EPR sample preparation and temperature optimization for RIDME; RIDME measurements and data processing and analysis; modeling; sensitivity considerations; supplementary RIDME data and CDA2.0 report; and references and author contributions (PDF)
Transparent Peer Review report available (PDF)
