# Supplementary material for: Pulse Dipolar Electron Paramagnetic Resonance Spectroscopy Distance Measurements at Low Nanomolar Concentrations: The CuII-Trityl Case
Source: J Phys Chem Lett. 2024 Jan 31;15(5):1455–61. doi: 10.1021/acs.jpclett.3c03311 (PMC10860127; doi:10.1021/acs.jpclett.3c03311)
Supplement: Supplementary file 1 — jz3c03311_si_001.pdf [file jz3c03311_si_001.pdf]

**Supporting Information**

# Pulse Dipolar Electron Paramagnetic Resonance Spectroscopy Distance Measurements at Low Nanomolar Concentrations: The Cu$^{II}$-Trityl Case

*Katrin Ackermann,[1] Caspar A. Heubach,[2] Olav Schiemann,[2]*, Bela E. Bode[1]**

[1]EaStCHEM School of Chemistry and Biomedical Sciences Research Complex, Centre of Magnetic Resonance, University of St Andrews, North Haugh, St Andrews, KY16 9ST, U.K.; [2]Clausius Institute of Physical and Theoretical Chemistry, University of Bonn, Wegelerstr. 12, 53115 Bonn, Germany.

AUTHOR INFORMATION

**Corresponding Author**

*schiemann@pc.uni-bonn.de; *beb2@st-andrews.ac.uk

# Table of Contents


1) Protein expression, purification and spin labelling ..... S3
2) Continuous wave (CW) EPR ..... S3
3) Electrospray ionization (ESI) mass spectrometry ..... S4
4) UV/VIS ..... S5
5) EPR sample preparation ..... S5
6) Temperature optimisation for RIDME measurements ..... S6
7) RIDME measurements ..... S8
8) RIDME data processing and analysis ..... S9
9) Modelling ..... S9
10) Sensitivity considerations ..... S10
11) Supplementary RIDME data ..... S12
   A) ctRIDME data set ..... S12
   B) ctvtRIDME data set ..... S14
12) References ..... S17
13) Author contributions ..... S18
14) CDA2.0 Reports ..... S19

## 1) Protein expression, purification and spin labelling

The I6C/K28H/Q32H construct of the immunoglobulin-binding B1 domain of group G streptococcal protein G (GB1) was purified as described previously.[1] Purified protein was SLIM-labelled according to published protocols,[2, 3] using a 3.5-fold molar excess of SLIM per cysteine. Successful labelling was confirmed via continuous wave (CW) EPR and mass spectrometric analysis (see below), and trityl and protein concentrations were determined via CW EPR spin counting and UV/VIS (see below) as described.[2, 3] Labelling with CuNTA was performed as reported previously.[1]

## 2) Continuous wave (CW) EPR

Room-temperature CW EPR measurements to assess labelling efficiency were performed using a Bruker EMX 10/12 spectrometer equipped with an ELEXSYS Super Hi-Q resonator at an operating frequency of ~9.9 GHz (X-band) with 100 kHz modulation.

Samples were recorded using a 150 G field sweep centred at 3505 G, a time constant of 20.48 ms, a conversion time of 20.02 ms, and 1500 points resolution. An attenuation of 20.0 dB (2 mW power) and a modulation amplitude of 0.2 G were used. The GB1 sample was measured in a 20 µL capillary at approximately 50 µM protein concentration and the double integral was compared to 4-Hydroxy-TEMPO (4-Hydroxy-2,2,6,6-tetramethylpiperidine 1-oxyl; Acros) as a standard. Labelling efficiency was determined as ~98%, corresponding to a protein concentration of 49 µM (Figure S1).

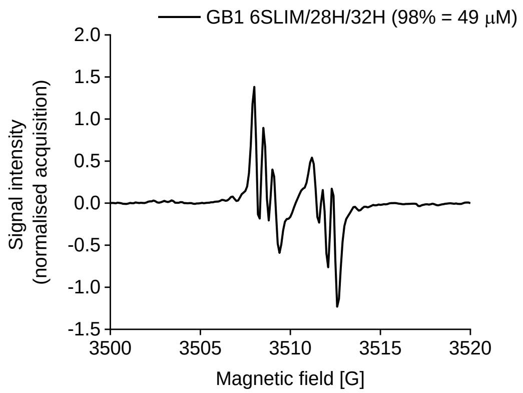


**Figure S1.** Room-temperature CW EPR spectrum for GB1 I6SLIM/K28H/Q32H.

## 3) Electrospray ionization (ESI) mass spectrometry

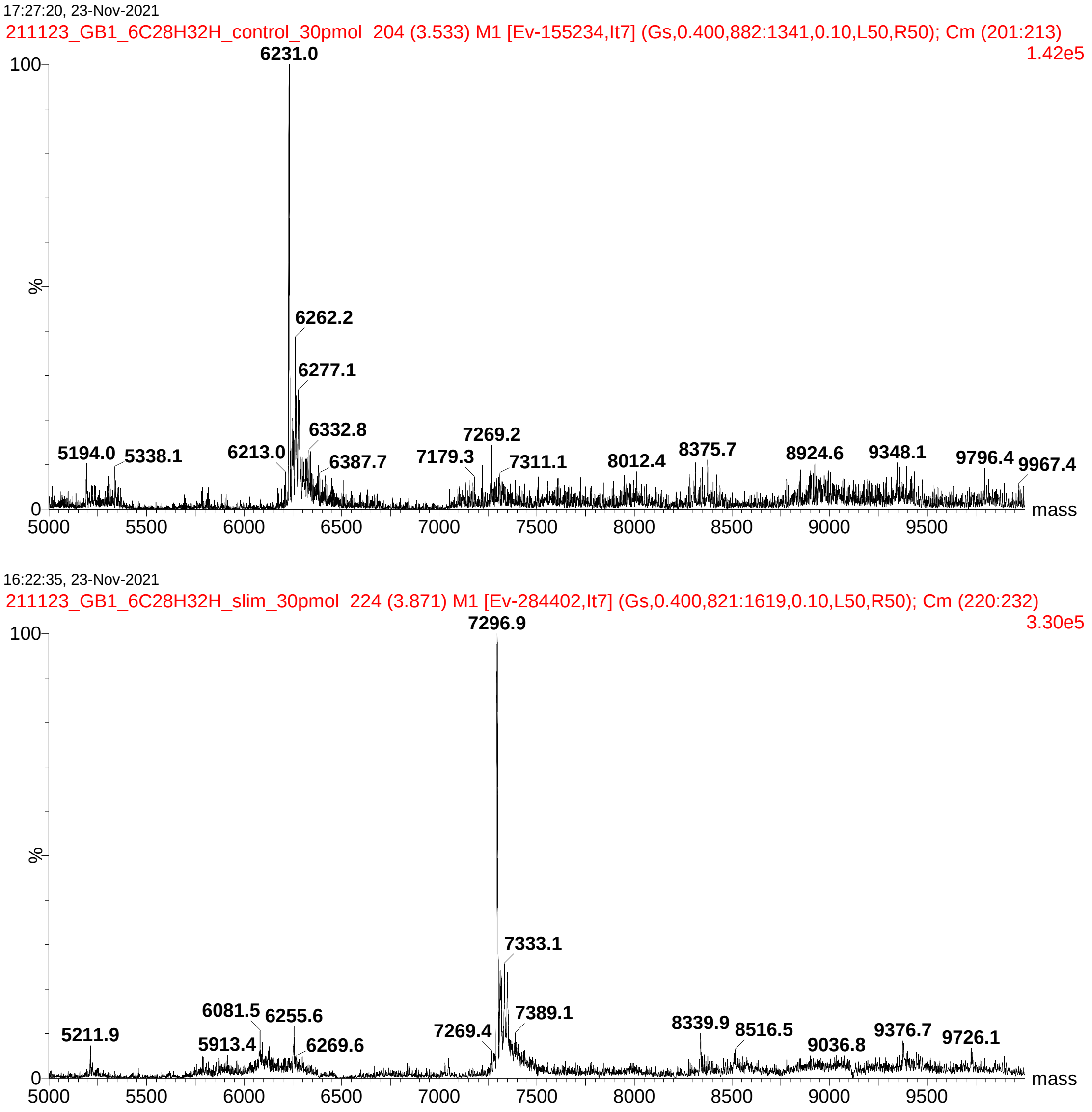


**Figure S2**. ESI mass spectrometry spectra for unlabelled (top) and SLIM-labelled (bottom) GB1 I6C/K28H/Q32H.

Successful spin labelling was confirmed via electrospray ionisation (ESI) mass spectrometry using the in-house mass spectrometry facility. ESI mass spectrometry was performed on samples before (control) and after spin labelling. Samples were diluted to 1 µM in 1% formic acid (FA). 30 pmol per sample were injected onto the liquid chromatography (LC) system (Waters Xevo G2 TOF MS with Acquity HPLC) using a MassPrep cartridge column (Waters), applying a 5 minute gradient

from 95% water, 5% acetonitrile to 5% water, 95% acetonitrile (eluents supplemented with 1% FA). Data were collected in positive mode from 500-2500 m/z, and charged ion series deconvolution to 0.1 Da resolution was performed using the MaxEnt I algorithm utilising a peak width at half height of 0.4 m/z. Expected masses were obtained before (6231 Da) and after (7297 Da) labelling (Figure S2).

## 4) UV/VIS

A UV/VIS spectrum from 200 nm to 800 nm of SLIM-labelled GB1 I6C/K28H/A32H was measured with a Jenway 6850 double beam spectrophotometer (Figure S3). Protein and trityl concentrations were determined according to the previously established protocol.[2]

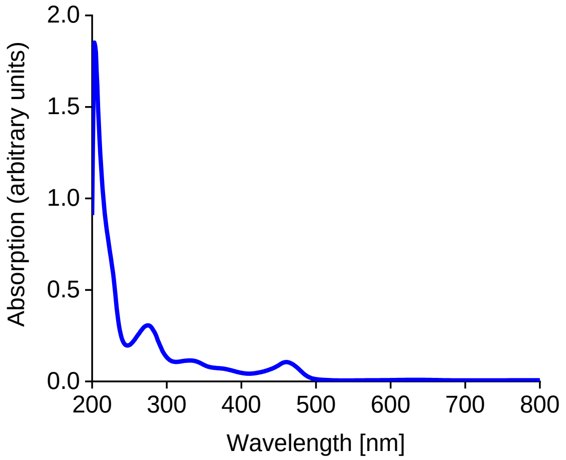


**Figure S3.** UV/VIS spectrum of SLIM-labelled GB1 I6C/K28H/Q32H.

## 5) EPR sample preparation

For RIDME experiments, samples with a final volume of 65 μL were prepared with varying protein and CuNTA concentrations (Table S1) in deuterated phosphate buffer (42.4 mM $Na_2HPO_4$, 7.6 mM $KH_2PO_4$, 150 mM NaCl, pH 7.4) and 50% (v/v) deuterated ethylene glycol (Deutero) for cryoprotection as described, with CuNTA concentrations determined to yield approximately 90% labelling of the α-helical double-histidine (dHis) site based on a dissociation constant of 200 nM.[1]

| Sample # | [GB1] | [CuNTA] |
|---|---|---|
| 1 | 5.0 µM | 6.5 µM |
| 2 | 500 nM | 2.25 µM |
| 3 | 100 nM | 2.0 µM |
| 4 | 50 nM | 2.0 µM |
| 5 | 25 nM | 2.0 µM |
| 6 | 10 nM | 2.0 µM |
| 7 | 5 nM | 2.0 µM |

**Table S1**. Overview of protein and CuNTA concentrations for each EPR sample.

## 6) Temperature optimisation for RIDME measurements

Temperature-dependent relaxation behaviour of SLIM was investigated via relaxation measurements performed between 30 K and 70 K using the 5 µM GB1 I6SLIM/K28H/Q32H sample to determine the temperature with the optimum sensitivity for RIDME measurements. Longitudinal relaxation times ($T_1$) were estimated from 3-pulse inversion recovery measurements under the monoexponential approximation. Transverse dephasing times ($T_m$, here ~$T_2$) were estimated from 2-pulse electron-spin echo decay measurements under the stretched exponential approximation. $T_1$, $T_m$, and corresponding 1/e and ½ × $1/e^2$ times for SLIM are given in Tables S2 and S3, respectively. Note that at all temperatures, the 1/e and ½ × $1/e^2$ times are very similar, suggesting that the SLIM relaxation behaviour is well met by the monoexponential approximation.

Subsequently, SLIM relaxation times were used to determine relative sensitivities per temperature (Figure S4). Here, for the CuNTA-SLIM label combination, the expression described previously[1] can be simplified by removing the last two terms: $(\exp(-T_{mix}/T_1^A))\times((1-\exp(-T_{mix}/T_1^B))/2)$, where A = SLIM and B = $Cu^{II}$ in this case. The second-last term can be neglected as it is only significantly different from 1 when $T_{mix}$ approaches or exceeds $T_1^A$, however $T_1^{SLIM}$ is several orders of magnitude longer than the chosen $T_{mix}$; the last term refers to the modulation depth, which is maximal with our chosen $T_{mix}$, so the term can be considered constant for all temperatures.

Thus, the expression is simplified to: SNR ≈ (1/T)×(exp(−2×$t_{max}$/$T_m^{SLIM}$))×(1/sqrt($T_1^{SLIM}$)), with SNR being the signal-to-noise ratio, $t_{max}$ was set to 2 µs and assuming a $T_{mix}$ of 60 µs (i.e., << $T_1^{SLIM}$).

| Temp | Mono-exponential $T_1$ | 95% conf. | 1/e time | ½ × 1/e² time |
|---|---|---|---|---|
| [K] | [ms] | | [ms] | [ms] |
| 30 | 51.2 | 51.1-51.3 | 49.8 | 50.4 |
| 35 | 29.3 | 29.2-29.3 | 28.8 | 29.2 |
| 40 | 18.5 | 18.4-18.5 | 18.0 | 18.1 |
| 45 | 12.5 | 12.4-12.5 | 12.0 | 12.3 |
| 50 | 9.24 | 9.21-9.26 | 9.15 | 9.4 |
| 60 | 5.82 | 5.80-5.85 | 5.80 | 5.85 |
| 70 | 3.49 | 3.47-3.50 | 3.50 | 3.58 |

**Table S2.** Estimated $T_1$ values under the monoexponential approximation for SLIM, including the 95% confidence bounds.

| Temp | Stretched exponential $T_m$ | Stretching exponent | 1/e time | ½ × 1/e² time |
|---|---|---|---|---|
| [K] | [µs] | | [µs] | [µs] |
| 30 | 3.67 | 1.0000 | 4.02 | 4.31 |
| 35 | 3.51 | 1.0000 | 3.74 | 4.00 |
| 40 | 3.27 | 1.0000 | 3.58 | 3.62 |
| 45 | 2.99 | 1.0000 | 3.36 | 3.26 |
| 50 | 2.70 | 1.0000 | 3.44 | 3.15 |
| 60 | 2.31 | 1.0000 | 2.86 | 2.52 |
| 70 | 2.11 | 1.0000 | 2.66 | 2.38 |

**Table S3**. Estimated $T_m$ values under the stretched exponential approximation for SLIM, including the stretching exponent.

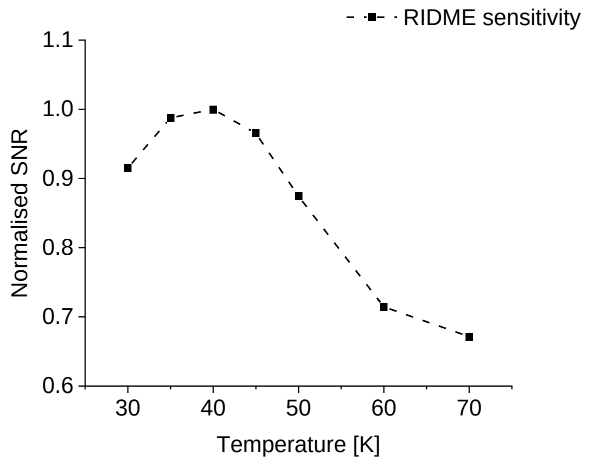


**Figure S4.** Sensitivity profile for CuNTA-SLIM RIDME, calculated using the equation given above (modified from Wort *et al.*[1]). A measurement temperature of 40 K shows maximal sensitivity. SNR = signal-to-noise ratio.

## 7) RIDME measurements

All RIDME experiments were performed using a Bruker ELEXSYS 580 pulse EPR spectrometer. Temperatures were maintained using a cryogen-free variable temperature cryostat (Cryogenic Ltd) operating in the 1.8-300 K temperature range. Samples were measured with the 5-pulse constant time (ct; $\pi/2$–$\tau_1$–$\pi$–($\tau_1$+$t$)–$\pi/2$–$T_{mix}$–$\pi/2$–($\tau_2$–$t$)–$\pi$–$\tau_2$–echo) RIDME[4] and the recently introduced variable time (vt; $\pi/2$–$\tau_1$–$\pi$–($\tau_1$+$t$)–$\pi/2$–$T_{mix}$–$\pi/2$–$\tau_0$–$\pi$–($\tau_2$+$t$)–echo) RIDME[5] experiment at 40 K, using a high-power 150 W travelling-wave tube (TWT; Applied Systems Engineering) at Q-band (34 GHz). For the initial ctRIDME measurement series, the 3 mm cylindrical resonator (Bruker ER 5106QT-2w) was critically coupled (highQ mode) as previously reported,[1, 6, 7] while for the ctvtRIDME (constant and variable time RIDME in a single combined pulse program) measurements it was used overcoupled (lowQ mode), since the $\tau_1$ in highQ is too long for efficient vtRIDME setup due to the increased ring-down time.[8] Pulses were applied on the maximum of the nitroxide echo detected field sweep. All RIDME measurements were performed with detection pulse lengths of 8 and 16 ns for $\pi/2$ and $\pi$, respectively, and each trace was acquired using an SRT of 26 ms, and a $\tau_1$ of 400 ns. For ctRIDME (highQ), 2 shots-per-loop, 8-step phase cycling, and a mixing time of 60 μs were used. For ctvtRIDME (lowQ) we used between 1 and 13 shots-per-loop and 32-step phase cycling, and for samples at 100 nM or higher protein concentration, a short

(reference) and a long mixing time of 5 and 60 μs, respectively, were recorded, to allow deconvolution of the traces.[5] For convenience, the resulting four experiments were combined into a single pulse programme, and samples at 500 nM, and 100 nM protein concentration were averaged for approximately 13 h, and 19 h, respectively (total time for all four experiments).

## 8) RIDME data processing and analysis

RIDME experiments were analysed using DeerAnalysis2022[9] (downloaded 3 May 2022) as previously described.[7] Briefly, data were subjected to Tikhonov regularization using a homogeneous 6-dimensional background function followed by statistical analysis (validation tool) varying background start from 5 to 30% of the trace length in 8 trials and varying the background dimension from 3 to 6 in 7 trials. Resulting best-fit background start time and dimension were subsequently used as starting points for a second round of Tikhonov regularization followed by a second round of statistical analysis, this time also including the addition of 50% random noise in 16 trials, except for the 5 nM sample, where only 10% random noise was added. Second round validation trials were pruned with a prune level of 1.15, where trials exceeding the root mean square deviation of the best fit by at least 15% are discarded. In all cases the regularization parameter $\alpha$ was chosen according to the L-curve criterion[10] (Lcc) or the generalised cross-validation (GCV),[11] and the goodness-of-fit. The Lcc was used as the standard, however for traces with lower modulation-to-noise ratio GCV was found more reliable to avoid over- or undersmoothing. The ct slice of the ctvtRIDME measurement of the 25 nM sample required manual choice of $\alpha$.

Additionally, RIDME data were analysed with deep neural network processing employing DEERNet[12] (Spinach Rev 5662) with specified RIDME background[13, 14] within the ComparativeDEERAnalyzer version 2.0 (CDA2.0) in DeerAnalysis2022 for comparison.

## 9) Modelling

The predicted distance distribution for GB1 I6SLIM/K28H/Q32H coordinated to CuNTA was modelled based on a GB1 crystal structure (PDB 4WH4).[15] A SLIM label was introduced at residue 6 and CuNTA at residues 28 and 32. Modelling was done using colab running mtsslWizard for

bipedal labels.[16, 17] The corresponding cartoon representation and simulated distance distribution are shown in Figure 1 of the main text.

## 10) Sensitivity considerations

To estimate sensitivity, RIDME modulation depths (Δ) were obtained during processing in DeerAnalysis2022 from the second round of Tikhonov regularisation (i.e., using optimised background start time and background dimension, see section 8) for details), and noise levels (root mean square deviation, RMSD), were calculated from the second and third quartile of the imaginary part of the phase-corrected RIDME traces as described.[6]

Sensitivities (S) were then determined as the ratio Δ to RMSD. S values were further divided by the square root of total echoes per point and multiplied with the square root of the averaging rate, yielding the sensitivity per unit time ($S_t$).[6] A summary of obtained values is given in Table S4, which also includes data from previous nanomolar studies,[1, 6, 7] and an extrapolation of $S_t$ to a hypothetical protein concentration of 1 μM for direct comparison of results.

Data revealed an increase in sensitivity of more than a factor three for the SLIM vs MTSL, and a further increase of approximately 2.5-fold when using the vtRIDME instead of the ctRIDME experiment.

The 25 nM sample is worse than extrapolation would suggest. As we cannot measure the final concentrations but just the stocks and then dilute this may be an error or just variation. However, we believe it would be unethical to remove a data point for no objective reason.

| GB1 | $Cu^{II}$ | Experiment | Δ | Δ | S (this study) | $S_t$ (sqrt Hz) | $S_t$ at 1 μM | Reference |
|---|---|---|---|---|---|---|---|---|
| [nM] | [μM] | | Tikh. | assumed | experiment | experiment | extrapolated | |
| 500 | 2.25 | $Cu^{II}$-SLIM RIDME, ct | 0.389 | n.a. | 78.54 | 10.764 | 21.527 | this study (Fig. S6) |
| 100 | 2 | $Cu^{II}$-SLIM RIDME, ct | 0.446 | n.a. | 18.74 | 2.297 | 22.970 | this study (Fig. S6) |
| 50 | 2 | $Cu^{II}$-SLIM RIDME, ct | 0.449 | n.a. | 10.75 | 1.042 | 20.830 | this study (Fig. S6) |
| 25 | 2 | $Cu^{II}$-SLIM RIDME, ct | 0.373 | n.a. | 2.82 | 0.273 | 10.931 | this study (Fig. S6) |
| 10 | 2 | $Cu^{II}$-SLIM RIDME, ct | 0.340 | n.a. | 2.46 | 0.151 | 15.064 | this study (Fig. S6) |
| 500 | 2.25 | $Cu^{II}$-SLIM RIDME, vt | 0.447 | n.a. | 205.3 | 28.134 | 56.269 | this study (Fig. 3 & S6) |
| 100 | 2 | $Cu^{II}$-SLIM RIDME, vt | 0.494 | n.a. | 42.05 | 5.154 | 51.536 | this study (Fig. 3 & S6) |
| 50 | 2 | $Cu^{II}$-SLIM RIDME, vt | 0.527 | n.a. | 25.54 | 2.475 | 49.497 | this study (Fig. 3 & S6) |
| 25 | 2 | $Cu^{II}$-SLIM RIDME, vt | 0.505 | n.a. | 7.90 | 0.766 | 30.629 | this study (Fig. 3 & S6) |
| 10 | 2 | $Cu^{II}$-SLIM RIDME, vt | 0.492 | n.a. | 8.64 | 0.529 | 52.934 | this study (Fig. 3 & S6) |
| 10 | 2 | $Cu^{II}$-SLIM RIDME, vt | 0.473 | n.a. | 12.50 | 0.425 | 42.510 | this study (Fig. 4) |
| 5 | 2 | $Cu^{II}$-SLIM RIDME, vt | 0.312 | n.a. | 3.16 | 0.118 | 23.605 | this study (Fig. S7) |
| 500 | 2.25 | $Cu^{II}$-SLIM RIDME, ct dec | 0.298 | n.a. | 50.25 | 6.886 | 13.772 | this study (Fig. S6) |
| 100 | 2 | $Cu^{II}$-SLIM RIDME, ct dec | 0.340 | n.a. | 9.60 | 1.176 | 11.765 | this study (Fig. S6) |
| 500 | 2.25 | $Cu^{II}$-SLIM RIDME, vt dec | 0.311 | n.a. | 70.97 | 9.725 | 19.450 | this study (Fig. S6) |
| 100 | 2 | $Cu^{II}$-SLIM RIDME, vt dec | 0.331 | n.a. | 12.11 | 1.484 | 14.840 | this study (Fig. S6) |
| | | | | | | | | |
| 500 | 2.25 | $Cu^{II}$-SLIM RIDME, ct | 0.38 | n.a. | 46.34 | 7.333 | 14.667 | this study (Fig. 2 & S5) |
| 100 | 2 | $Cu^{II}$-SLIM RIDME, ct | 0.409 | n.a. | 20.54 | 1.877 | 18.767 | this study (Fig. 2 & S5) |
| 50 | 2 | $Cu^{II}$-SLIM RIDME, ct | 0.391 | n.a. | 17.9 | 0.962 | 19.242 | this study (Fig. 2 & S5) |
| 25 | 2 | $Cu^{II}$-SLIM RIDME, ct | 0.332 | n.a. | 7.02 | 0.227 | 9.064 | this study (Fig. 2 & S5) |
| 10 | 2 | $Cu^{II}$-SLIM RIDME, ct | 0.333 | n.a. | 5.37 | 0.173 | 17.335 | this study (Fig. 2 & S5) |
| | | | | | | | | |
| 50 | 8.1 | $Cu^{II}$-NO RIDME, ct | 0.427 | n.a. | | 0.172 | 3.440 | [7] |
| 50 | 2.7 | $Cu^{II}$-NO RIDME, ct | 0.371 | n.a. | | 0.269 | 5.376 | [7] |
| 50 | 0.9 | $Cu^{II}$-NO RIDME, ct | 0.308 | n.a. | | 0.198 | 3.959 | [7] |
| 50 | 0.3 | $Cu^{II}$-NO RIDME, ct | 0.247 | n.a. | | 0.182 | 3.650 | [7] |
| 50 | 0.1 | $Cu^{II}$-NO RIDME, ct | 0.188 | n.a. | | 0.152 | 3.043 | [7] |
| | | | | | | | | |
| 50 | 8.1 | $Cu^{II}$-NO RIDME, ct | 0.41 | n.a. | | 0.257 | 5.147 | [7] |
| 50 | 2.7 | $Cu^{II}$-NO RIDME, ct | 0.427 | n.a. | | 0.314 | 6.275 | [7] |
| 50 | 0.9 | $Cu^{II}$-NO RIDME, ct | 0.388 | n.a. | | 0.205 | 4.110 | [7] |
| 50 | 0.3 | $Cu^{II}$-NO RIDME, ct | 0.243 | n.a. | | 0.204 | 4.077 | [7] |
| 50 | 0.1 | $Cu^{II}$-NO RIDME, ct | 0.292 | n.a. | | 0.207 | 4.135 | [7] |
| | | | | | | | | |
| 100 | 8.1 | $Cu^{II}$-NO RIDME, ct | 0.418 | n.a. | | 0.792 | 7.918 | [7] |
| 100 | 2.7 | $Cu^{II}$-NO RIDME, ct | 0.425 | n.a. | | 0.591 | 5.909 | [7] |
| 100 | 0.9 | $Cu^{II}$-NO RIDME, ct | 0.340 | n.a. | | 0.428 | 4.284 | [7] |
| 100 | 0.3 | $Cu^{II}$-NO RIDME, ct | 0.323 | n.a. | | 0.452 | 4.516 | [7] |
| 100 | 0.1 | $Cu^{II}$-NO RIDME, ct | 0.206 | n.a. | | 0.272 | 2.719 | [7] |
| | | | | | | | | |
| 25000 | 30 | Dummy $Cu^{II}$-NO PELDOR | n.a. | 0.300 | | 68.414 | 2.737 | [7] |
| 25000 | 30 | Dummy $Cu^{II}$-NO RIDME LQ | n.a. | 0.450 | | 79.796 | 3.192 | [7] |
| 25000 | 30 | Dummy $Cu^{II}$-NO RIDME HQ | n.a. | 0.450 | | 143.009 | 5.720 | [7] |
| | | | | | | | | |
| 25000 | 50 | Dummy $Cu^{II}$-$Cu^{II}$ PELDOR | n.a. | 0.010 | | 0.658 | 0.026 | [1] |
| 25000 | 50 | Dummy $Cu^{II}$-$Cu^{II}$ RIDME HQ | n.a. | 0.200 | | 62.000 | 2.480 | [1] |
| 25000 | 30 | Dummy $Cu^{II}$-NO RIDME HQ | n.a. | 0.450 | | 99.100 | 3.964 | [1] |
| | | | | | | | | |
| 100 | 0 | NO-NO PELDOR | 0.223 | n.a. | | 0.300 | 3.002 | [6] |
| 500 | 0 | NO-NO PELDOR | 0.292 | n.a. | | 4.465 | 8.930 | [6] |
| 500 | 1.6 | $Cu^{II}$-$Cu^{II}$ RIDME | 0.055 | n.a. | | 0.452 | 0.905 | [6] |

**Table S4.** Modulation depths Δ, sensitivities (this study), and sensitivities per unit time ($S_t$) obtained for the ctRIDME and ctvtRIDME experiments from processing using Tikhonov regularisation. References for data from previous studies are indicated. All $S_t$ values are extrapolated to a protein concentration of 1 μM for direct comparison (see highlighted rows for convenience).

# 11) Supplementary RIDME data

## A) ctRIDME data set

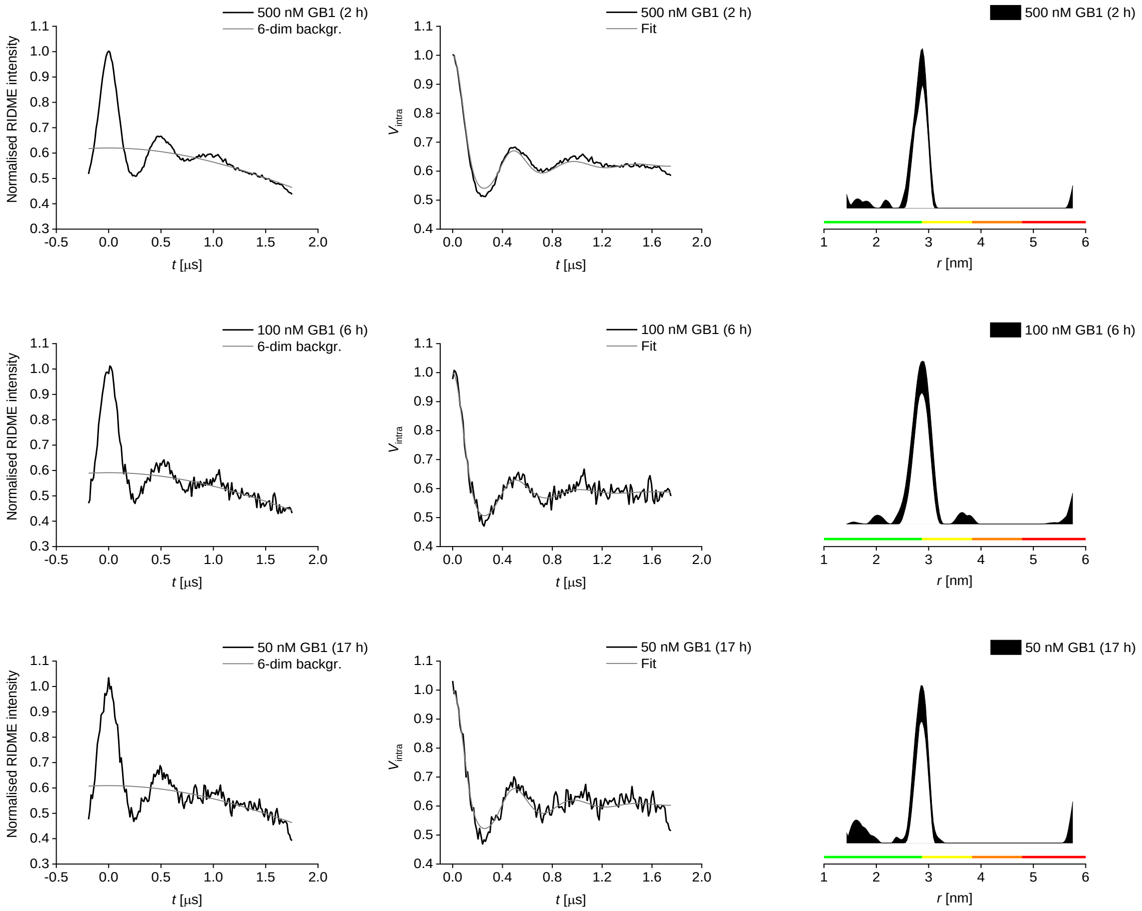


**Figure S5.** ctRIDME data for the GB1 I6SLIM/K28H/Q32H dilution series. Raw (left) and background-corrected (middle) ctRIDME traces with 6-dimensional background and fit (grey), respectively, for the 500 nM, 100 nM, 50 nM, 25 nM and 10 nM samples. Right: Corresponding distance distributions given as 95% confidence estimates (± 2s) with 50% noise added for error estimation during statistical analysis. Colour bars represent reliability ranges (green: shape reliable; yellow: mean and width reliable; orange: mean reliable; red: no quantification possible).

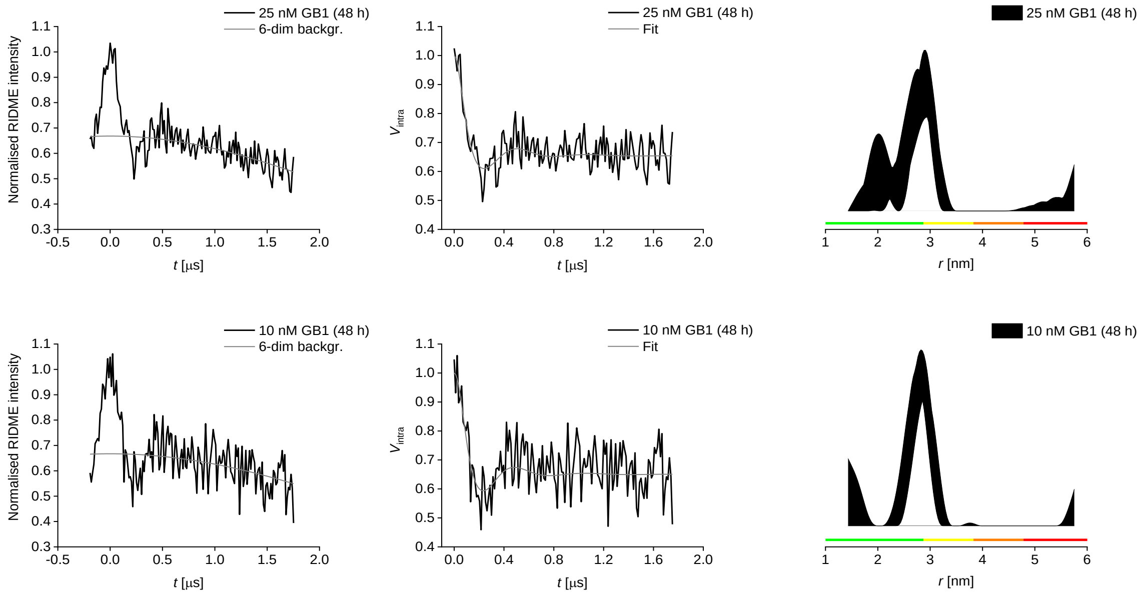


**Figure S5, cont.** ctRIDME data for the GB1 I6SLIM/K28H/Q32H dilution series. Raw (left) and background-corrected (middle) ctRIDME traces with 6-dimensional background and fit (grey), respectively, for the 500 nM, 100 nM, 50 nM, 25 nM and 10 nM samples. Right: Corresponding distance distributions given as 95% confidence estimates (± 2s) with 50% noise added for error estimation during statistical analysis. Colour bars represent reliability ranges (green: shape reliable; yellow: mean and width reliable; orange: mean reliable; red: no quantification possible).

## B) ctvtRIDME data set

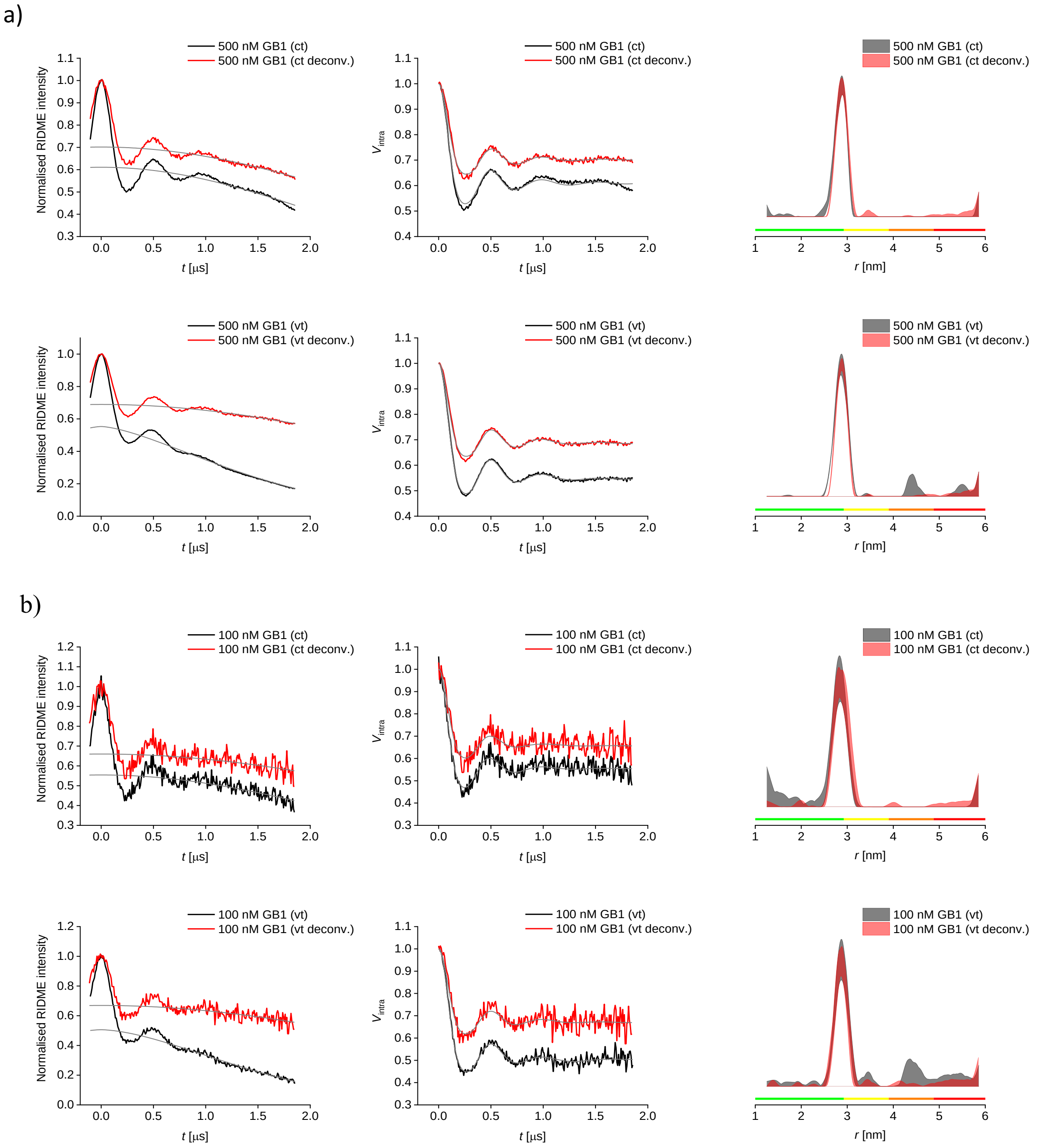


**Figure S6**. ctvtRIDME data for the GB1 I6SLIM/K28H/Q32H dilution series. Raw (left) and background-corrected (middle) RIDME traces for the 500 nM (a), 100 nM (b), 50 nM (c), 25 nM (d) and 10 nM (e) samples. Deconvoluted data are provided as available (red), as indicated on each plot. Right: Corresponding distance distributions given as 95% confidence estimates (± 2s) with 50% noise added for error estimation during statistical analysis. Colour bars represent reliability ranges (green: shape reliable; yellow: mean and width reliable; orange: mean reliable; red: no quantification possible).

c)

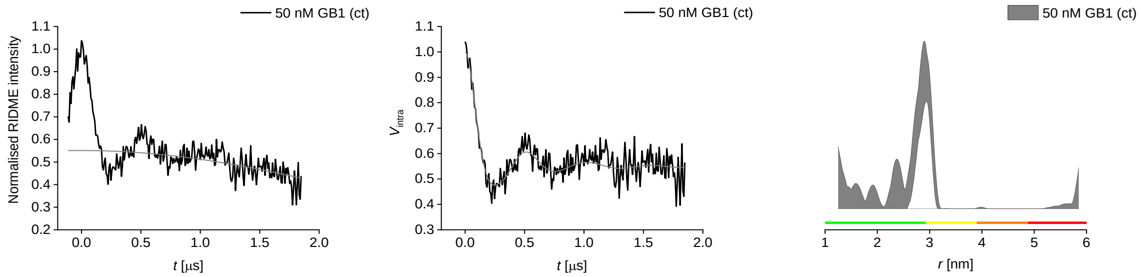


d)

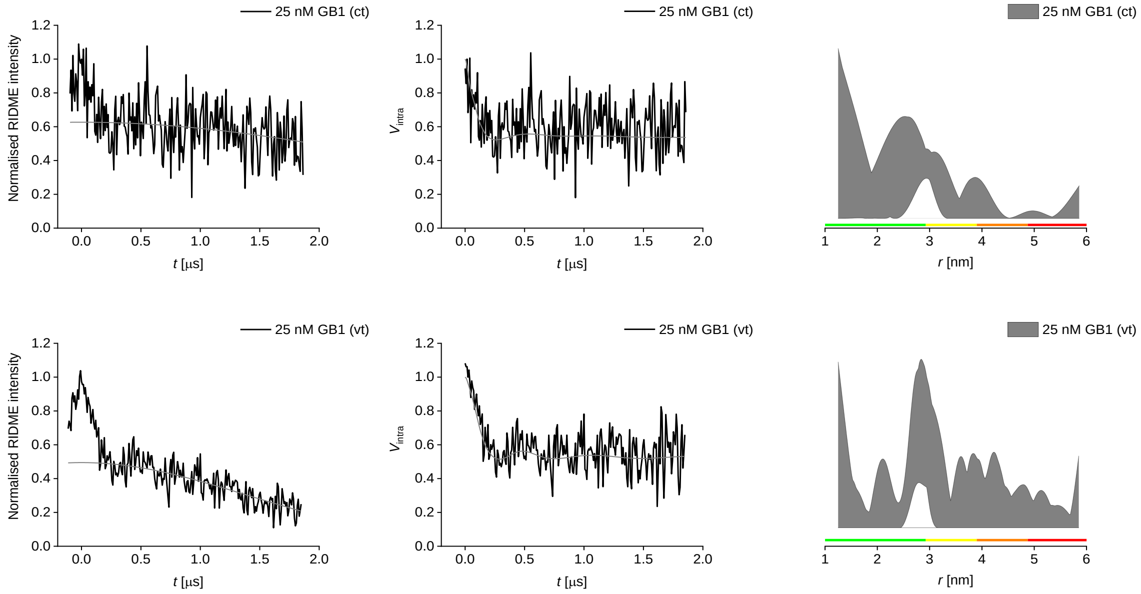


**Figure S6, continued**. ctvtRIDME data for the GB1 I6SLIM/K28H/Q32H dilution series. Raw (left) and background-corrected (middle) RIDME traces for the 500 nM (a), 100 nM (b), 50 nM (c), 25 nM (d) and 10 nM (e) samples. Deconvoluted data are provided as available (red), as indicated on each plot. Right: Corresponding distance distributions given as 95% confidence estimates (± 2s) with 50% noise added for error estimation during statistical analysis. Colour bars represent reliability ranges (green: shape reliable; yellow: mean and width reliable; orange: mean reliable; red: no quantification possible).

e)

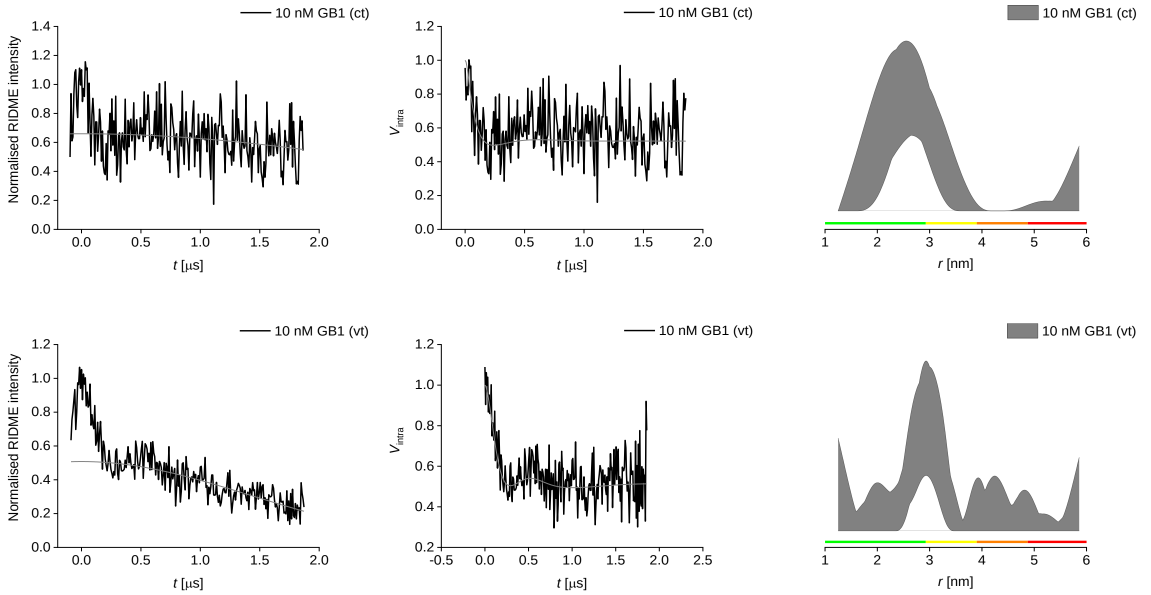


**Figure S6, continued**. ctvtRIDME data for the GB1 I6SLIM/K28H/Q32H dilution series. Raw (left) and background-corrected (middle) RIDME traces for the 500 nM (a), 100 nM (b), 50 nM (c), 25 nM (d) and 10 nM (e) samples. Deconvoluted data are provided as available (red), as indicated on each plot. Right: Corresponding distance distributions given as 95% confidence estimates (± 2s) with 50% noise added for error estimation during statistical analysis. Colour bars represent reliability ranges (green: shape reliable; yellow: mean and width reliable; orange: mean reliable; red: no quantification possible).

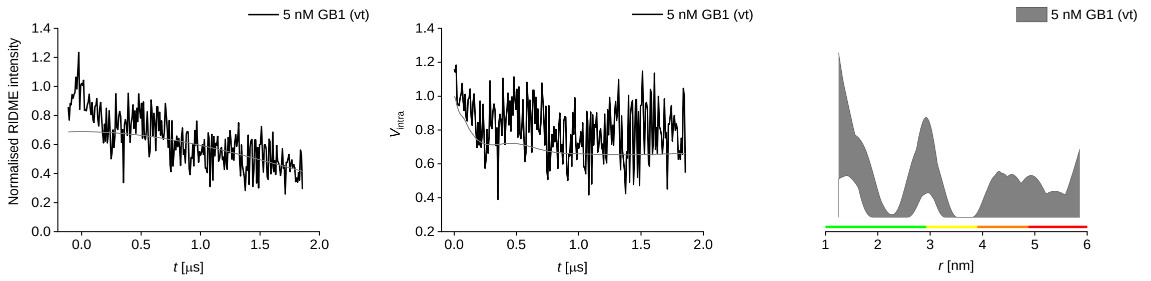


**Figure S7.** vtRIDME data for 5 nM GB1 I6SLIM/K28H/Q32H. Raw (left) and background-corrected (middle) RIDME trace with background and fit (grey), respectively. Right: Corresponding distance distribution given as 95% confidence estimate (± 2s) with 10% noise added for error estimation during statistical analysis. Colour bars represent reliability ranges (green: shape reliable; yellow: mean and width reliable; orange: mean reliable; red: no quantification possible). Note that the achieved modulation-to-noise was considered too low to deliver a distance distribution fit for interpretation.

## 12) References


(1) Wort, J. L.; Ackermann, K.; Giannoulis, A.; Stewart, A. J.; Norman, D. G.; Bode, B. E. Sub-Micromolar Pulse Dipolar EPR Spectroscopy Reveals Increasing $Cu^{II}$-labelling of Double-Histidine Motifs with Lower Temperature. *Angew. Chem. Int. Ed.* **2019**, *58*, 11681-11685.

(2) Fleck, N.; Heubach, C. A.; Hett, T.; Haege, F. R.; Bawol, P. P.; Baltruschat, H.; Schiemann, O. SLIM: A Short-Linked, Highly Redox-Stable Trityl Label for High-Sensitivity In-Cell EPR Distance Measurements. *Angew. Chem. Int. Ed.* **2020**, *59*, 9767-9772.

(3) Heubach, C. A.; Hasanbasri, Z.; Abdullin, D.; Reuter, A.; Korzekwa, B.; Saxena, S.; Schiemann, O. Differentiating between Label and Protein Conformers in Pulsed Dipolar EPR Spectroscopy with the dHis-$Cu^{2+}$(NTA) Motif. *Chem. Eur. J.* **2023**, e202302541.

(4) Milikisyants, S.; Scarpelli, F.; Finiguerra, M. G.; Ubbink, M.; Huber, M. A Pulsed EPR Method to Determine Distances Between Paramagnetic Centers with Strong Spectral Anisotropy and Radicals: The Dead-time Free RIDME Sequence. *J. Magn. Reson.* **2009**, *201*, 48-56.

(5) Wort, J. L.; Ackermann, K.; Giannoulis, A.; Bode, B. E. Enhanced sensitivity for pulse dipolar EPR spectroscopy using variable-time RIDME. *J. Magn. Reson.* **2023**, *352*, 107460.

(6) Ackermann, K.; Wort, J. L.; Bode, B. E. Nanomolar Pulse Dipolar EPR Spectroscopy in Proteins: $Cu^{II}$–$Cu^{II}$ and Nitroxide–Nitroxide Cases. *J. Phys. Chem. B* **2021**, *125*, 5358-5364.

(7) Ackermann, K.; Wort, J. L.; Bode, B. E. Pulse Dipolar EPR for Determining Nanomolar Binding Affinities. *Chem. Commun.* **2022**, *58*, 8790-8793.

(8) Vitali, V.; Ackermann, K.; Hagelueken, G.; Bode, B. E. Spectroscopically Orthogonal Labelling to Disentangle Site-Specific Nitroxide Label Distributions. *Appl. Magn. Reson.* **2023**.

(9) Jeschke, G.; Chechik, V.; Ionita, P.; Godt, A.; Zimmermann, H.; Banham, J.; Timmel, C. R.; Hilger, D.; Jung, H. DeerAnalysis2006 - a Comprehensive Software Package for Analyzing Pulsed ELDOR Data. *Appl. Magn. Reson.* **2006**, *30*, 473-498.

(10) Chiang, Y. W.; Borbat, P. P.; Freed, J. H. The Determination of Pair Distance Distributions by Pulsed ESR using Tikhonov Regularization. *J. Magn. Reson.* **2005**, *172*, 279-295.

(11) Edwards, T. H.; Stoll, S. Optimal Tikhonov Regularization for DEER Spectroscopy. *J Magn Reson* **2018**, *288*, 58-68.

(12) Worswick, S. G.; Spencer, J. A.; Jeschke, G.; Kuprov, I. Deep Neural Network Processing of DEER Data. *Sci. Adv.* **2018**, *4*, eaat5218.

(13) Keeley, J.; Choudhury, T.; Galazzo, L.; Bordignon, E.; Feintuch, A.; Goldfarb, D.; Russell, H.; Taylor, M. J.; Lovett, J. E.; Eggeling, A.; Fábregas Ibáñez, L.; Keller, K.; Yulikov, M.; Jeschke, G.; Kuprov, I. Neural Networks in Pulsed Dipolar Spectroscopy: A Practical Guide. *J. Magn. Reson.* **2022**, *338*, 107186.

(14) Keller, K.; Qi, M.; Gmeiner, C.; Ritsch, I.; Godt, A.; Jeschke, G.; Savitsky, A.; Yulikov, M. Intermolecular Background Decay in RIDME Experiments. *Phys. Chem. Chem. Phys.* **2019**, *21*, 8228-8245.

(15) Cunningham, T. F.; Putterman, M. R.; Desai, A.; Horne, W. S.; Saxena, S. The Double-Histidine $Cu^{2+}$-Binding Motif: a Highly Rigid, Site-Specific Spin Probe for Electron Spin Resonance Distance Measurements. *Angew. Chem. Int. Ed.* **2015**, *54*, 6330-6334.

(16) https://colab.research.google.com/github/gha2012/mtsslWizard_colab/blob/main/mtsslWizard_colab.ipynb. Accessed 25 September 2023.

(17) Hagelueken, G.; Ward, R.; Naismith, J. H.; Schiemann, O. MtsslWizard: In Silico Spin-Labeling and Generation of Distance Distributions in PyMOL. *Appl. Magn. Reson.* **2012**, *42*, 377-391.

## 13) Author contributions

*Katrin Ackermann*: Conceptualisation (supporting), data curation (lead), formal analysis (lead), funding acquisition (supporting), investigation (lead), methodology (equal), writing-original draft (lead), writing-review & editing (equal)

*Caspar Heubach*: Conceptualisation (supporting), formal analysis (supporting), investigation (supporting), methodology (equal), writing-review & editing (equal)

*Olav Schiemann*: Conceptualisation (equal), investigation (supporting), formal analysis (supporting), funding acquisition (equal), methodology (supporting), supervision (equal), writing-review & editing (equal)

*Bela E. Bode*: Conceptualisation (equal), investigation (supporting), formal analysis (supporting), funding acquisition (equal), methodology (supporting), supervision (equal), writing-original draft (supporting), writing-review & editing (equal)

## 14) CDA2.0 Reports

In the following, the full CDA2.0 reports obtained for the ctRIDME (Table S5) and the ctvtRIDME (Table S6) measurements are attached.

| Sample # | [GB1] | [CuNTA] | CDA2.0 report name |
|---|---|---|---|
| 2 | 500 nM | 2.25 µM | 220607_KAq198.2_RIDME_comparative_DEER_analyzer_report |
| 3 | 100 nM | 2.0 µM | 220607_KAq198.3_RIDME_comparative_DEER_analyzer_report |

**Table S5.** CDA2.0 reports for ctRIDME measurements.

| Sample # | [GB1] | [CuNTA] | | CDA2.0 report name |
|---|---|---|---|---|
| 2 | 500 nM | 2.25 μM | ct | 230413_78.60_vtctRIDME_ct_comparative_DEER_analyzer_report |
| | | | ct dec. | 230413_78.60_vtctRIDME_ct_deconv_comparative_DEER_analyzer_report |
| | | | vt | 230413_78.60_vtctRIDME_vt_comparative_DEER_analyzer_report |
| | | | vt dec. | 230413_78.60_vtctRIDME_vt_deconv_comparative_DEER_analyzer_report |
| 3 | 100 nM | 2.0 μM | ct | 2330414_KAq213.2_vtctRIDME_5_60_ct_comparative_DEER_analyzer_report |
| | | | ct dec. | 2330414_KAq213.2_vtctRIDME_5_60_ct_deconv_comparative_DEER_analyzer_report |
| | | | vt | 2330414_KAq213.2_vtctRIDME_5_60_vt_comparative_DEER_analyzer_report |
| | | | vt dec. | 2330414_KAq213.2_vtctRIDME_5_60_vt_deconv_comparative_DEER_analyzer_report |
| 4 | 50 nM | 2.0 μM | ct | 2330417_KAq213.4_vtctRIDME_60_1_comparative_DEER_analyzer_report |
| | | | vt | 2330417_KAq213.4_vtctRIDME_60_0_comparative_DEER_analyzer_report |
| 5 | 25 nM | 2.0 μM | vt | 2330418_KAq213.6_vtctRIDME_60_0_comparative_DEER_analyzer_report |

**Table S6.** CDA2.0 reports for ctvtRIDME measurements (dec. = deconvoluted).

# DEER analysis report on dataset 220607_KAq198.2_RIDME

## DEERNet Spinach SVN Rev 5662 and DeerLab 0.9.1 Tikhonov regularization

### ComparativeDEERAnalyzer version 2.0

see: S. G. Worswick et al., DOI: 10.1126/sciadv.aat5218, L. Fabregas Ibanez et al., DOI: 10.5194/mr-1-209-2020

25-Jan-2023 10:47:23

# 1. Distance distributions

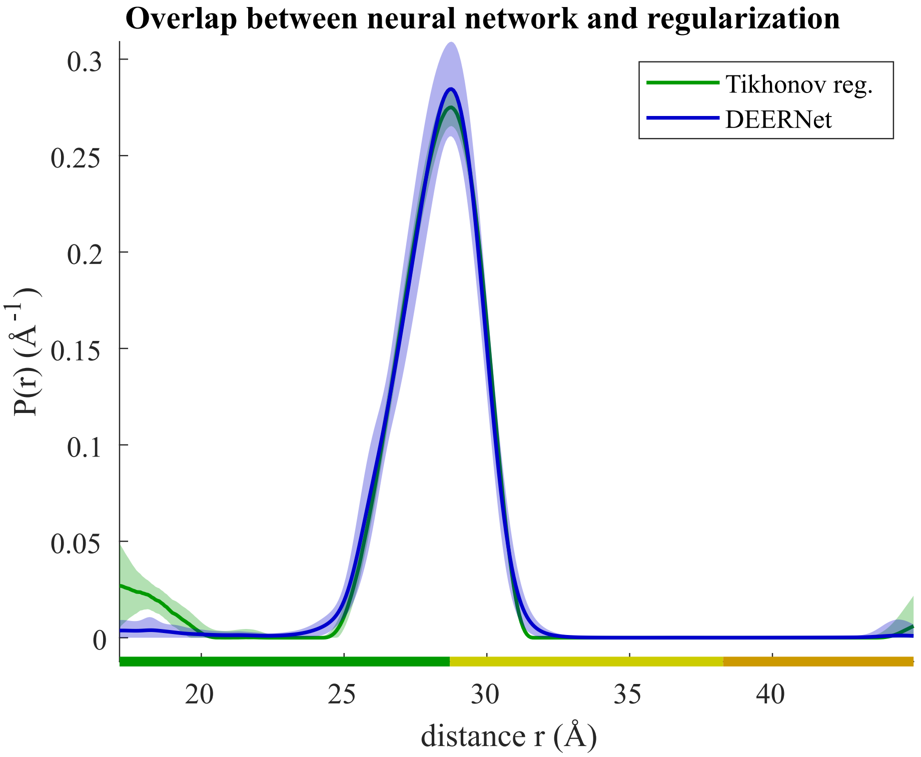


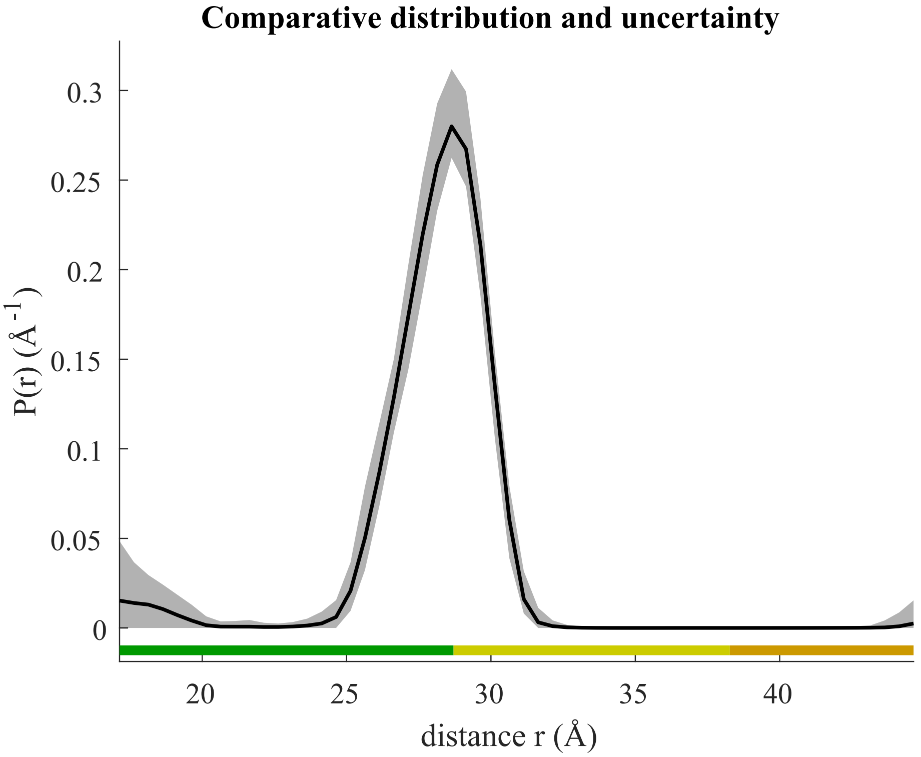

## 2. Fits of time-domain data

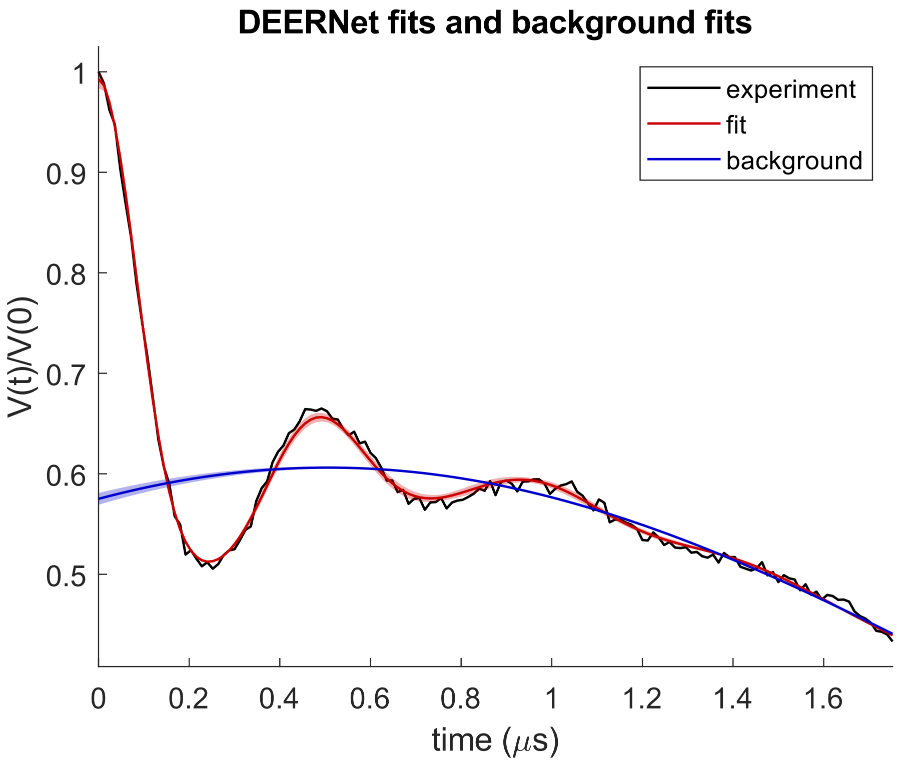


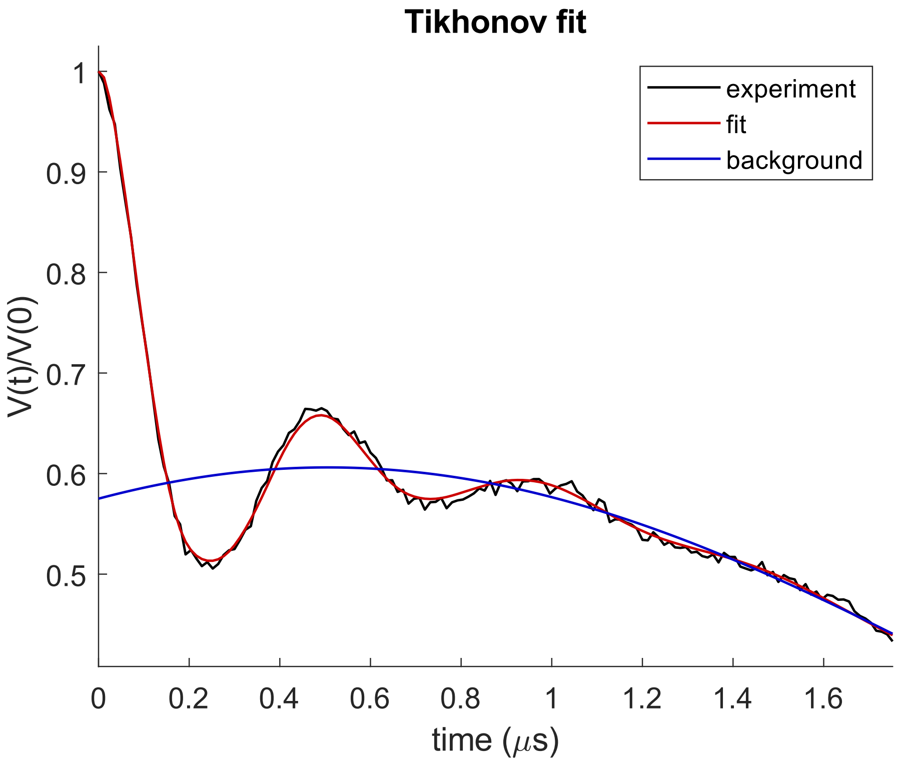

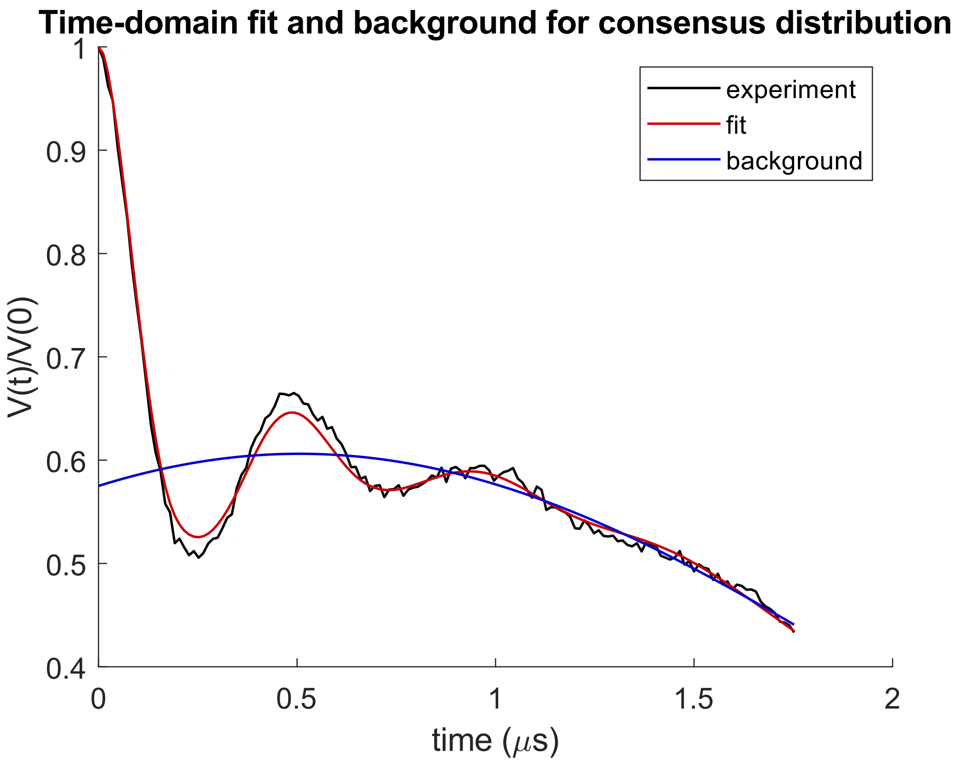
Time-domain fit and background for consensus distribution
experiment
fit
background
V(t)/V(0)
1
0.9
0.8
0.7
0.6
0.5
0.4
0
0.5
1
1.5
2
time ($\mu$s)

## 3. Experimental and processing parameters

**RIDME processing was requested. Only DEERNet output.**

Modulation depth: 0.421

Signal-to-noise ratio: 69.8 (w.r.t. modulation)

Noise estimates normalized to maximum signal

From imaginary part: 0.01173

From DEERNet fit: 0.00602

From Tikhonov fit: 0.00948

Zero time: 0 ns

Maximum time: 1752 ns

Time increment: 12 ns

Phase: 5.0 degree

Ensemble of 32 neural networks

Background separation by neural network

Background dimension: 3

Regularization parameter by best overlap with neural network solution

Regularization parameter used: 0.79

Reg. par. initial estimate by L-curve corner: 1.58

Overlap between DEERNet and regularization solutions: 0.949

Predicted overlap of consensus solution with ground truth: 0.81...0.99

Mean distance: 28.4 Å

Distance standard deviation: 1.2 Å

Full data set in Matlab format: C:\Users\ka44\Documents\OneDrive - University of St Andrews\StAndrews\Work\BEB\Projects\GB1\GB1_Nanomolar\GB1_SLIM\GB1_Cu_SLIM\GB1_Cu_SLIM_Qband\KAq198_6SLIM_28H32H_series\KAq198\500nM_198_2\220607_KAq198.2_RIDME_comparative_DEER_analysis.mat

Distance distributions in text format: C:\Users\ka44\Documents\OneDrive - University of St Andrews\StAndrews\Work\BEB\Projects\GB1\GB1_Nanomolar\GB1_SLIM\GB1_Cu_SLIM\GB1_Cu_SLIM_Qband\KAq198_6SLIM_28H32H_series\KAq198\500nM_198_2\220607_KAq198.2_RIDME_consensus_DEER_distribution.csv

Fit and background in text format: C:\Users\ka44\Documents\OneDrive - University of St Andrews\StAndrews\Work\BEB\Projects\GB1\GB1_Nanomolar\GB1_SLIM\GB1_Cu_SLIM\GB1_Cu_SLIM_Qband\KAq198_6SLIM_28H32H_series\KAq198\500nM_198_2\220607_KAq198.2_RIDME_consensus_DEER_fit.csv

Metadata: C:\Users\ka44\Documents\OneDrive - University of St Andrews\StAndrews\Work\BEB\Projects\GB1\GB1_Nanomolar\GB1_SLIM\GB1_Cu_SLIM\GB1_Cu_SLIM_Qband\KAq198_6SLIM_28H32H_series\KAq198\500nM_198_2\220607_KAq198.2_RIDME_comparative_DEER_meta_data.csv

# DEER analysis report on dataset 220607_KAq198.3_RIDME

## DEERNet Spinach SVN Rev 5662 and DeerLab 0.9.1 Tikhonov regularization

### ComparativeDEERAnalyzer version 2.0

see: S. G. Worswick et al., DOI: 10.1126/sciadv.aat5218, L. Fabregas Ibanez et al., DOI: 10.5194/mr-1-209-2020

25-Jan-2023 11:36:22

## 1. Distance distributions

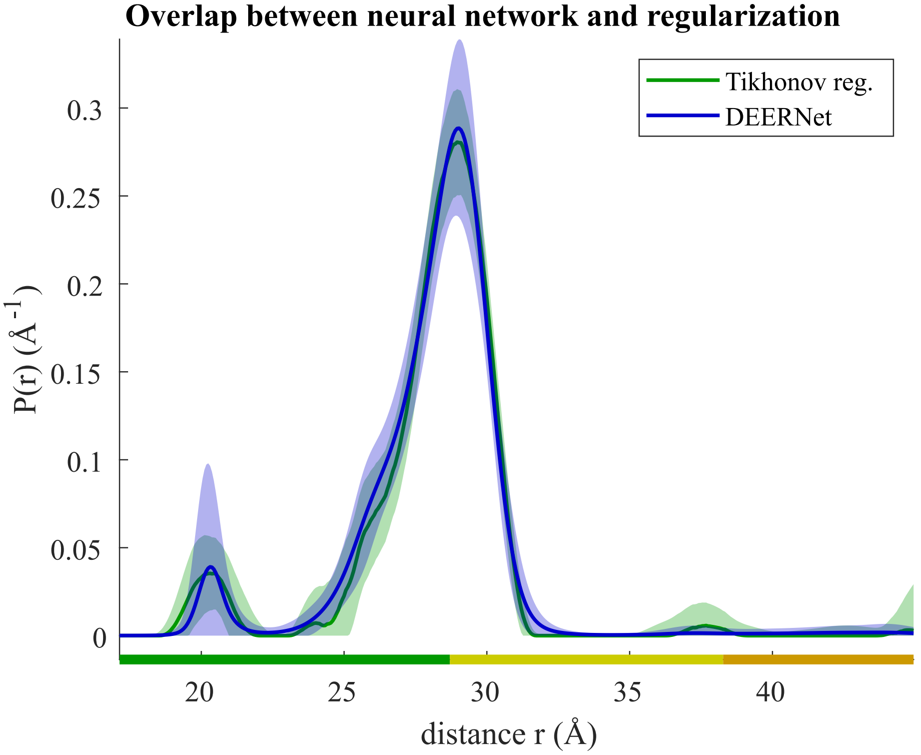


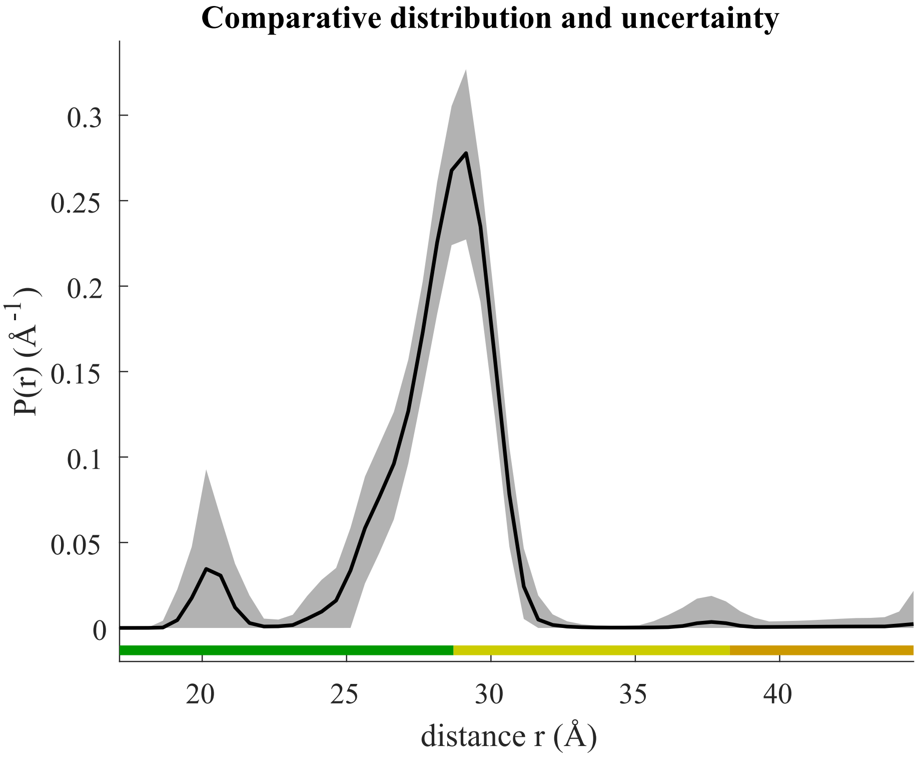

## 2. Fits of time-domain data

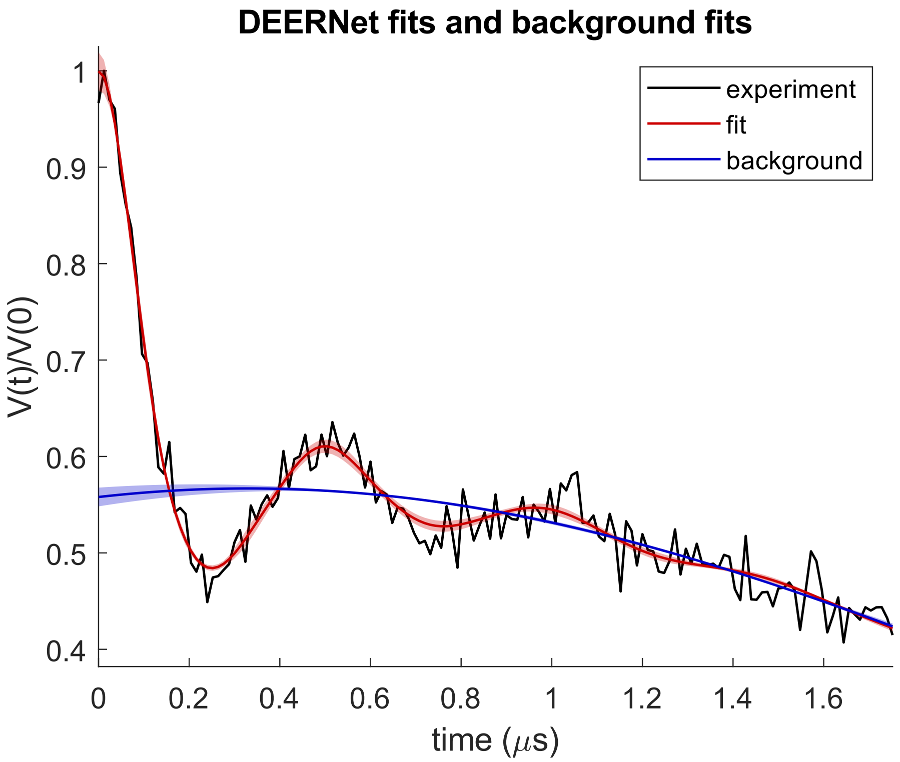


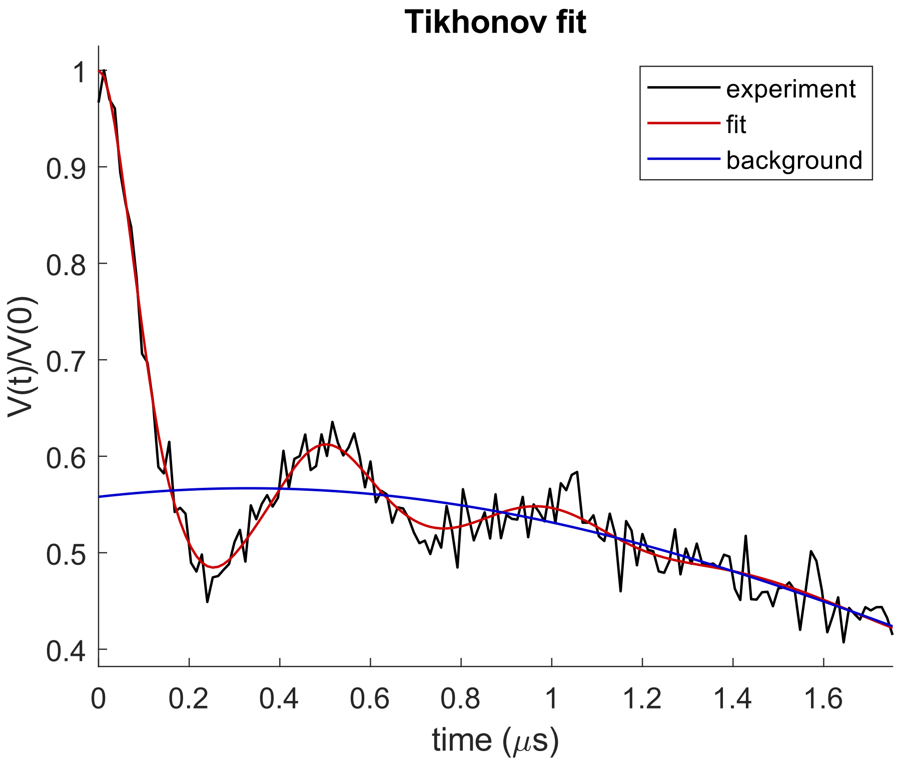

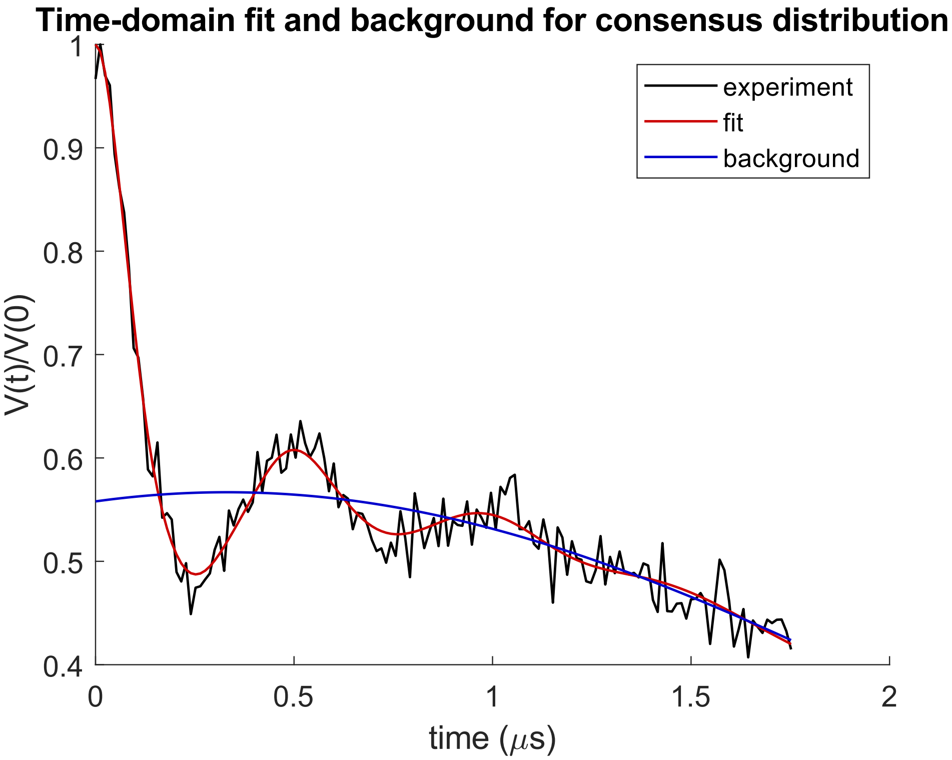
Time-domain fit and background for consensus distribution
experiment
fit
background
V(t)/V(0)
time ($\mu$s)
0
0.5
1
1.5
2
0.4
0.5
0.6
0.7
0.8
0.9
1

## 3. Experimental and processing parameters

**RIDME processing was requested. Only DEERNet output.**

Modulation depth: 0.442

Signal-to-noise ratio: 22.8 (w.r.t. modulation)

Noise estimates normalized to maximum signal

From imaginary part: 0.01911

From DEERNet fit: 0.01941

From Tikhonov fit: 0.01932

Zero time: -1 ns

Maximum time: 1752 ns

Time increment: 12 ns

Phase: -34.4 degree

Ensemble of 32 neural networks

Background separation by neural network

Background dimension: 3

Regularization parameter by best overlap with neural network solution

Regularization parameter used: 0.70

Reg. par. initial estimate by L-curve corner: 3.98

Overlap between DEERNet and regularization solutions: 0.940

Predicted overlap of consensus solution with ground truth: 0.81...0.98

Mean distance: 28.5 Å

Distance standard deviation: 1.4 Å

Full data set in Matlab format: C:\Users\ka44\Documents\OneDrive - University of St Andrews\StAndrews\Work\BEB\Projects\GB1\GB1_Nanomolar\GB1_SLIM\GB1_Cu_SLIM\GB1_Cu_SLIM_Qband\KAq198_6SLIM_28H32H_series\KAq198\100nM_198_3\220607_KAq198.3_RIDME_comparative_DEER_analysis.mat

Distance distributions in text format: C:\Users\ka44\Documents\OneDrive - University of St Andrews\StAndrews\Work\BEB\Projects\GB1\GB1_Nanomolar\GB1_SLIM\GB1_Cu_SLIM\GB1_Cu_SLIM_Qband\KAq198_6SLIM_28H32H_series\KAq198\100nM_198_3\220607_KAq198.3_RIDME_consensus_DEER_distribution.csv

Fit and background in text format: C:\Users\ka44\Documents\OneDrive - University of St Andrews\StAndrews\Work\BEB\Projects\GB1\GB1_Nanomolar\GB1_SLIM\GB1_Cu_SLIM\GB1_Cu_SLIM_Qband\KAq198_6SLIM_28H32H_series\KAq198\100nM_198_3\220607_KAq198.3_RIDME_consensus_DEER_fit.csv

Metadata: C:\Users\ka44\Documents\OneDrive - University of St Andrews\StAndrews\Work\BEB\Projects\GB1\GB1_Nanomolar\GB1_SLIM\GB1_Cu_SLIM\GB1_Cu_SLIM_Qband\KAq198_6SLIM_28H32H_series\KAq198\100nM_198_3\220607_KAq198.3_RIDME_comparative_DEER_meta_data.csv

# DEER analysis report on dataset 230413_78.60_vtctRIDME_ct

## DEERNet Spinach SVN Rev 5662 and DeerLab 0.9.1 Tikhonov regularization

### ComparativeDEERAnalyzer version 2.0

see: S. G. Worswick et al., DOI: 10.1126/sciadv.aat5218, L. Fabregas Ibanez et al., DOI: 10.5194/mr-1-209-2020

11-Sep-2023 17:10:18

## 1. Distance distributions

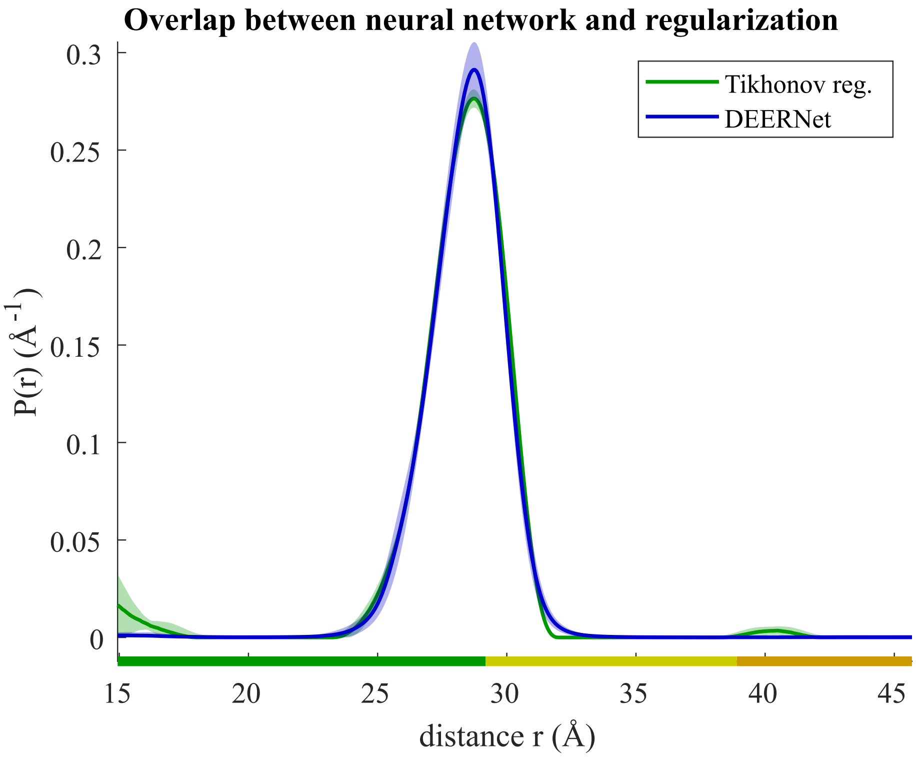


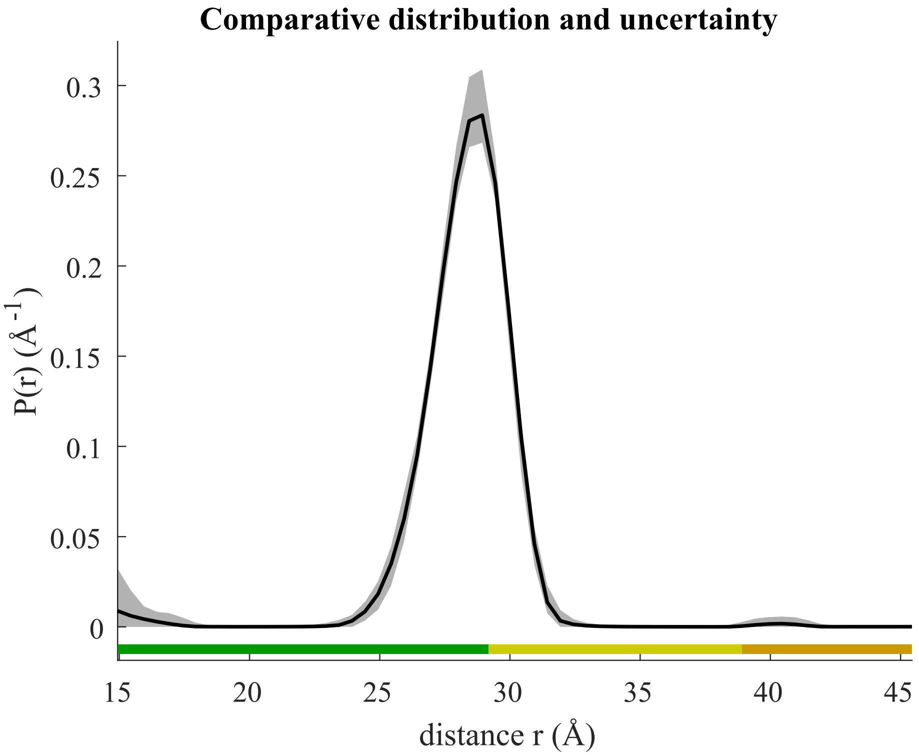

## 2. Fits of time-domain data

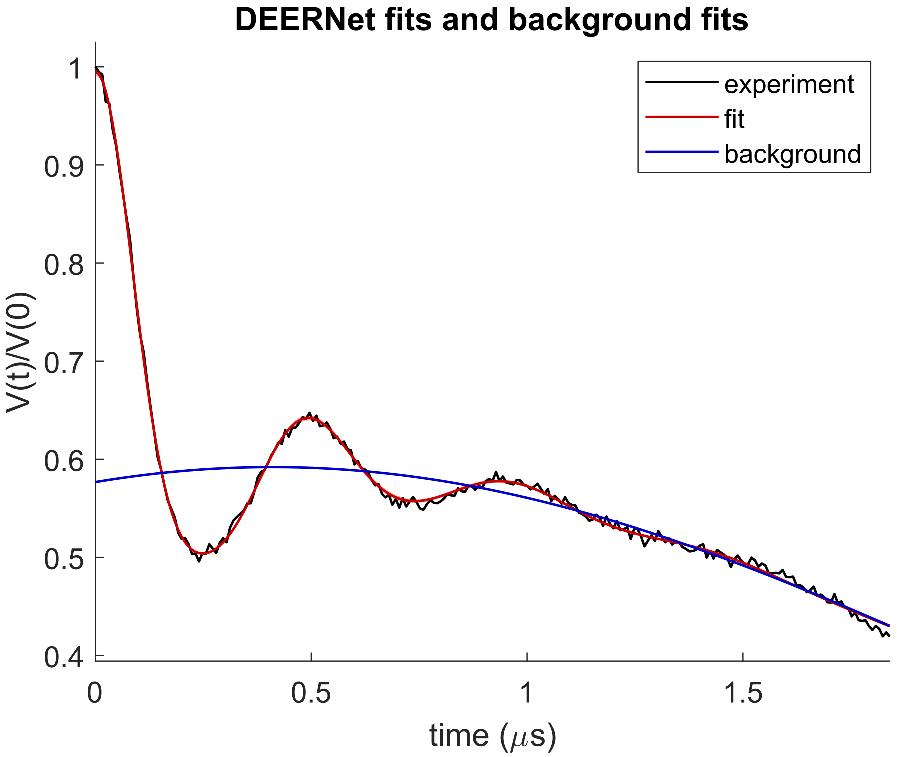


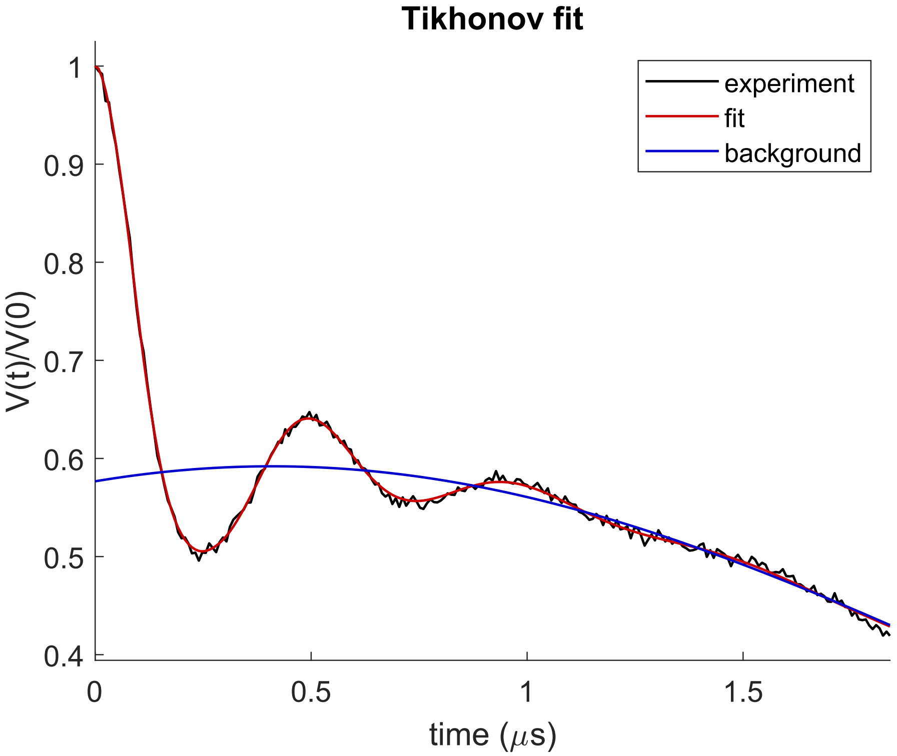

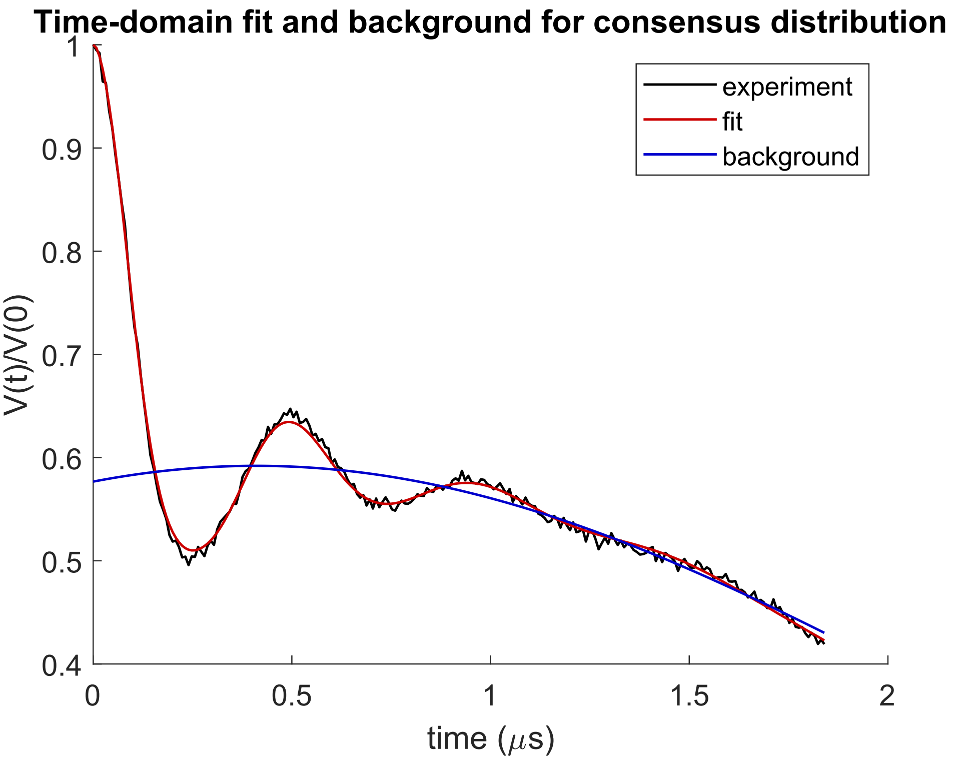
Time-domain fit and background for consensus distribution
experiment
fit
background
V(t)/V(0)
time ($\mu$s)
1
0.9
0.8
0.7
0.6
0.5
0.4
0
0.5
1
1.5
2

## 3. Experimental and processing parameters

**RIDME processing was requested. Only DEERNet output.**

Modulation depth: 0.420

Signal-to-noise ratio: 91.4 (w.r.t. modulation)

Noise estimates normalized to maximum signal

From imaginary part: 0.00583

From DEERNet fit: 0.00460

From Tikhonov fit: 0.00530

Zero time: 9 ns

Maximum time: 1840 ns

The last 1 % of the data was cut off

Time increment: 8 ns

Phase: 0.0 degree

Ensemble of 32 neural networks

Background separation by neural network

Background dimension: 3

Regularization parameter by best overlap with neural network solution

Regularization parameter used: 1.12

Reg. par. initial estimate by L-curve corner: 1.58

Overlap between DEERNet and regularization solutions: 0.959

Predicted overlap of consensus solution with ground truth: 0.82...0.99

Mean distance: 28.5 Å

Distance standard deviation: 1.3 Å

Full data set in Matlab format:
C:\Users\Katrin\Desktop\BEBQ78_500nM_ct\230413_78.60_vtctRIDME_ct_comparative_DEER_analysis.mat

Distance distributions in text format:
C:\Users\Katrin\Desktop\BEBQ78_500nM_ct\230413_78.60_vtctRIDME_ct_consensus_DEER_distribution.csv

Fit and background in text format:
C:\Users\Katrin\Desktop\BEBQ78_500nM_ct\230413_78.60_vtctRIDME_ct_consensus_DEER_fit.csv

Metadata:
C:\Users\Katrin\Desktop\BEBQ78_500nM_ct\230413_78.60_vtctRIDME_ct_comparative_DEER_meta_data.csv

# DEER analysis report on dataset 230413_78.60_vtctRIDME_ct_deconv

## DEERNet Spinach SVN Rev 5662 and DeerLab 0.9.1 Tikhonov regularization

### ComparativeDEERAnalyzer version 2.0

see: S. G. Worswick et al., DOI: 10.1126/sciadv.aat5218, L. Fabregas Ibanez et al., DOI: 10.5194/mr-1-209-2020

12-Sep-2023 09:19:31

## 1. Distance distributions

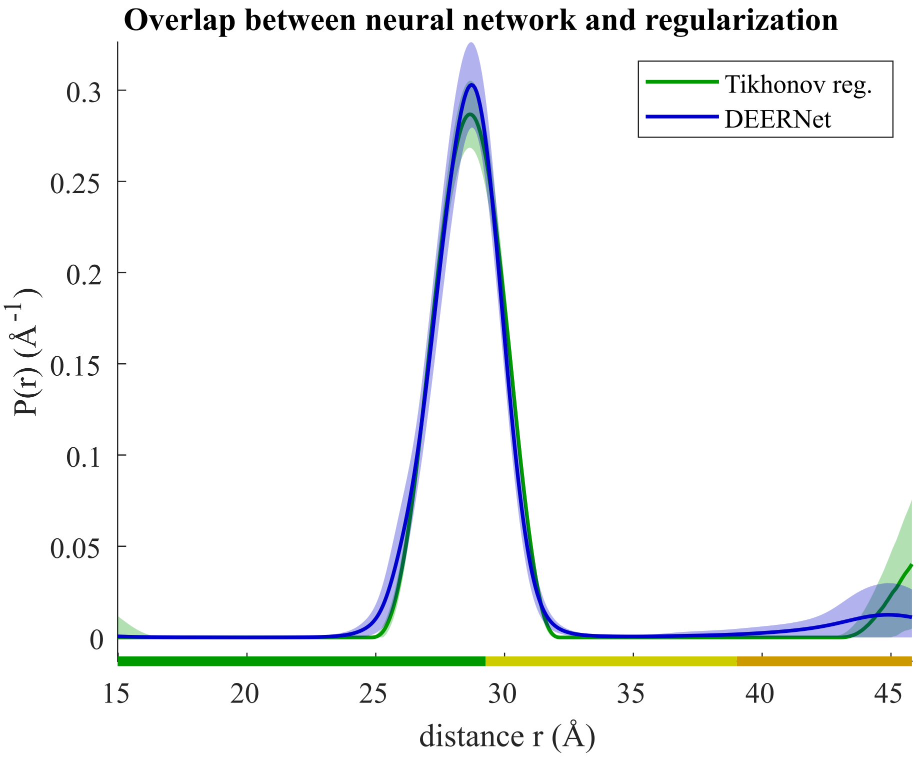


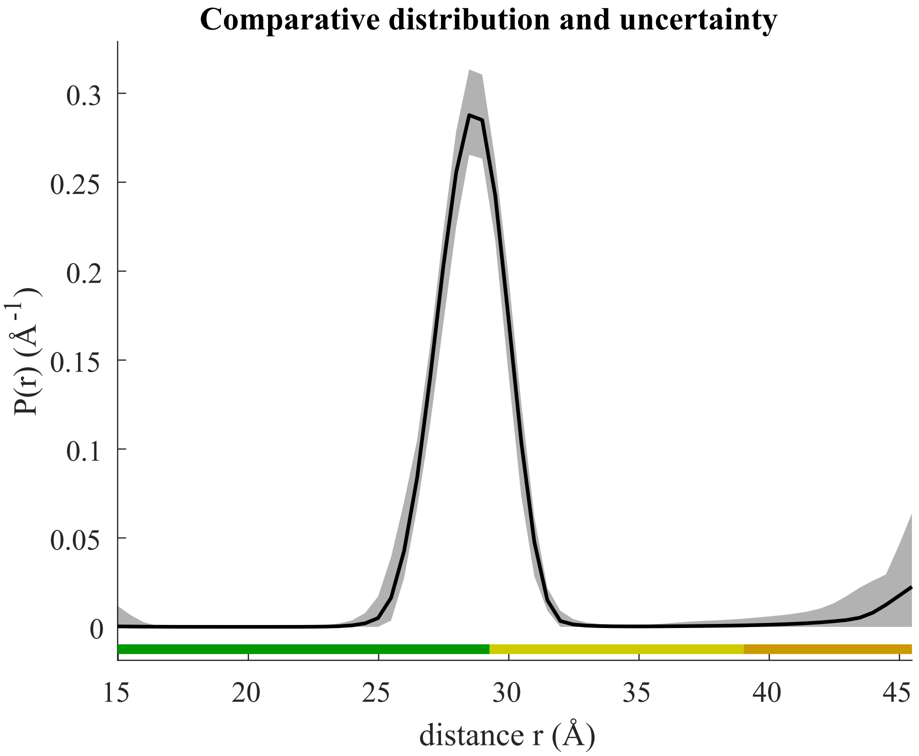

## 2. Fits of time-domain data

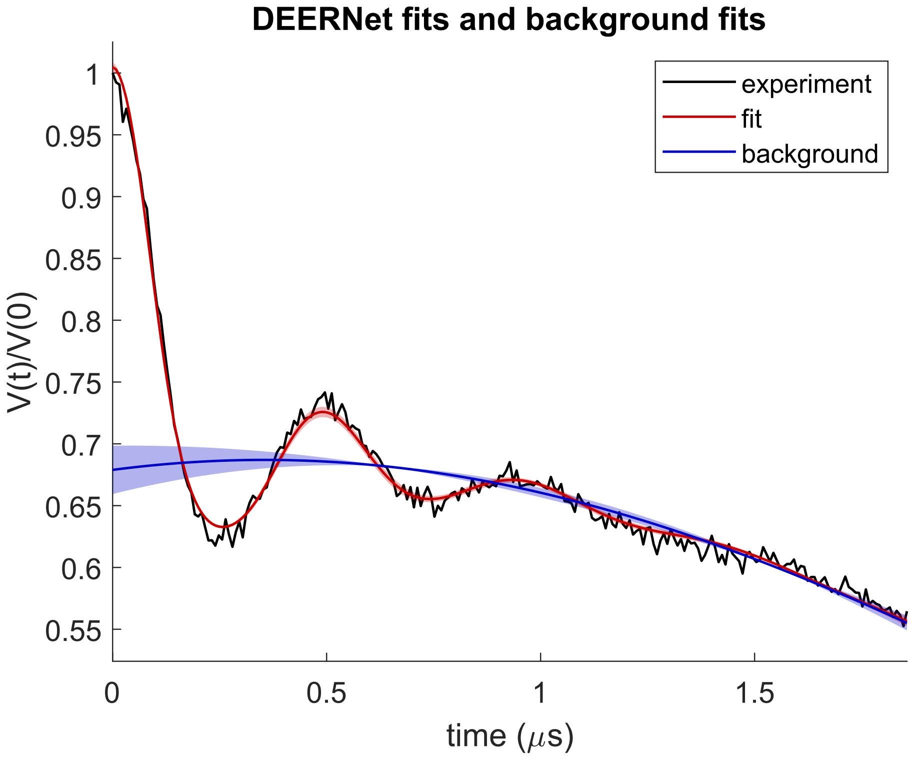


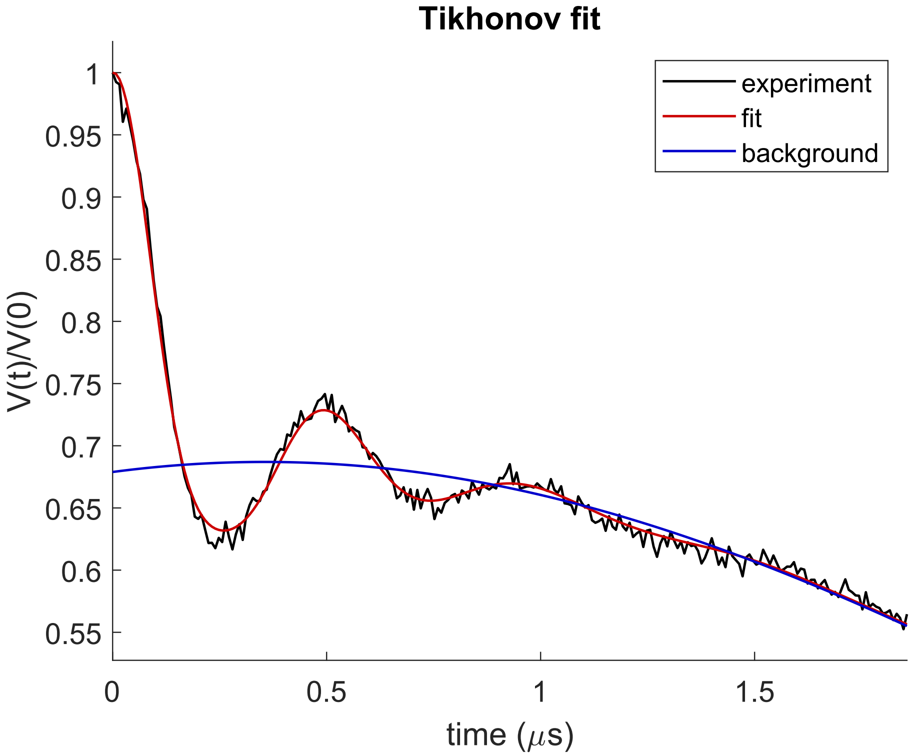

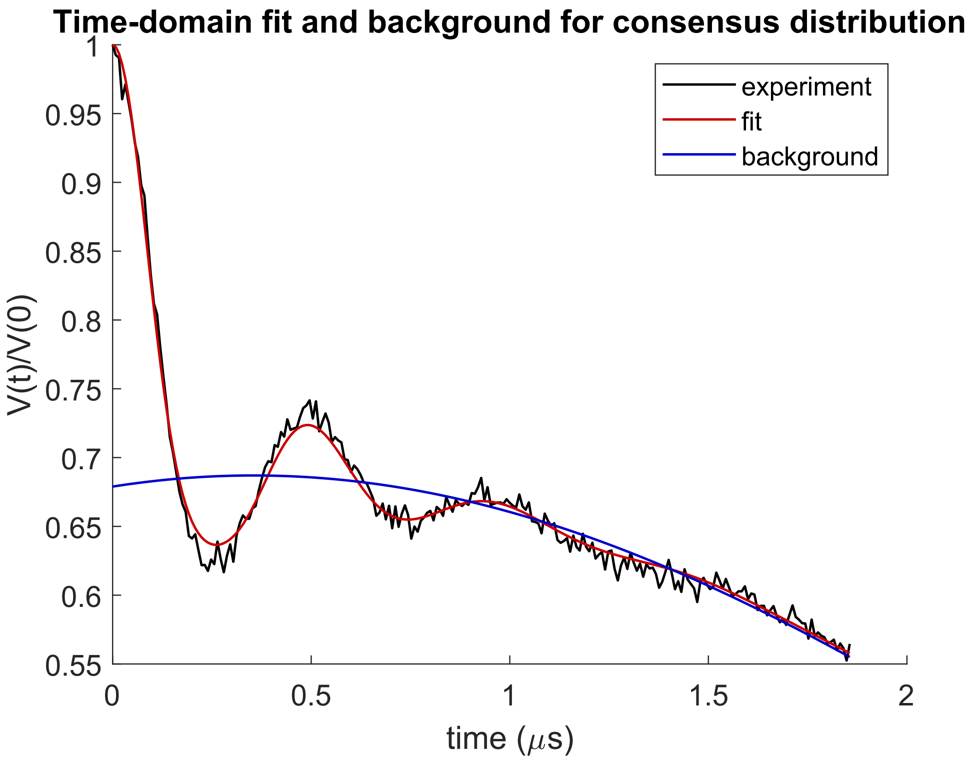
Time-domain fit and background for consensus distribution
experiment
fit
background
V(t)/V(0)
1
0.95
0.9
0.85
0.8
0.75
0.7
0.65
0.6
0.55
0
0.5
1
1.5
2
time (μs)

## 3. Experimental and processing parameters

**RIDME processing was requested. Only DEERNet output.**

Modulation depth: 0.324

Signal-to-noise ratio: 43.6 (w.r.t. modulation)

Noise estimates normalized to maximum signal

From imaginary part: 0.00613

From DEERNet fit: 0.00743

From Tikhonov fit: 0.00762

Zero time: 6 ns

Maximum time: 1856 ns

Time increment: 8 ns

Phase: -0.1 degree

Ensemble of 32 neural networks

Background separation by neural network

Background dimension: 3

Regularization parameter by best overlap with neural network solution

Regularization parameter used: 1.26

Reg. par. initial estimate by L-curve corner: 2.51

Overlap between DEERNet and regularization solutions: 0.933

Predicted overlap of consensus solution with ground truth: 0.80...0.97

Mean distance: 28.7 Å

Distance standard deviation: 1.2 Å

Full data set in Matlab format: C:\Users\Katrin\Desktop\BEBQ78_500nM_ct_deconv\230413_78.60_vtctRIDME_ct_deconv_comparative_DEER_analysis.mat

Distance distributions in text format: C:\Users\Katrin\Desktop\BEBQ78_500nM_ct_deconv\230413_78.60_vtctRIDME_ct_deconv_consensus_DEER_distribution.csv

Fit and background in text format:
C:\Users\Katrin\Desktop\BEBQ78_500nM_ct_deconv\230413_78.60_vtctRIDME_ct_deconv_consensus_DEER_fit.csv

Metadata:
C:\Users\Katrin\Desktop\BEBQ78_500nM_ct_deconv\230413_78.60_vtctRIDME_ct_deconv_comparative_DEER_meta_data.csv

# DEER analysis report on dataset 230413_78.60_vtctRIDME_vt

## DEERNet Spinach SVN Rev 5662 and DeerLab 0.9.1 Tikhonov regularization

### ComparativeDEERAnalyzer version 2.0

see: S. G. Worswick et al., DOI: 10.1126/sciadv.aat5218, L. Fabregas Ibanez et al., DOI: 10.5194/mr-1-209-2020

12-Sep-2023 09:37:21

## 1. Distance distributions

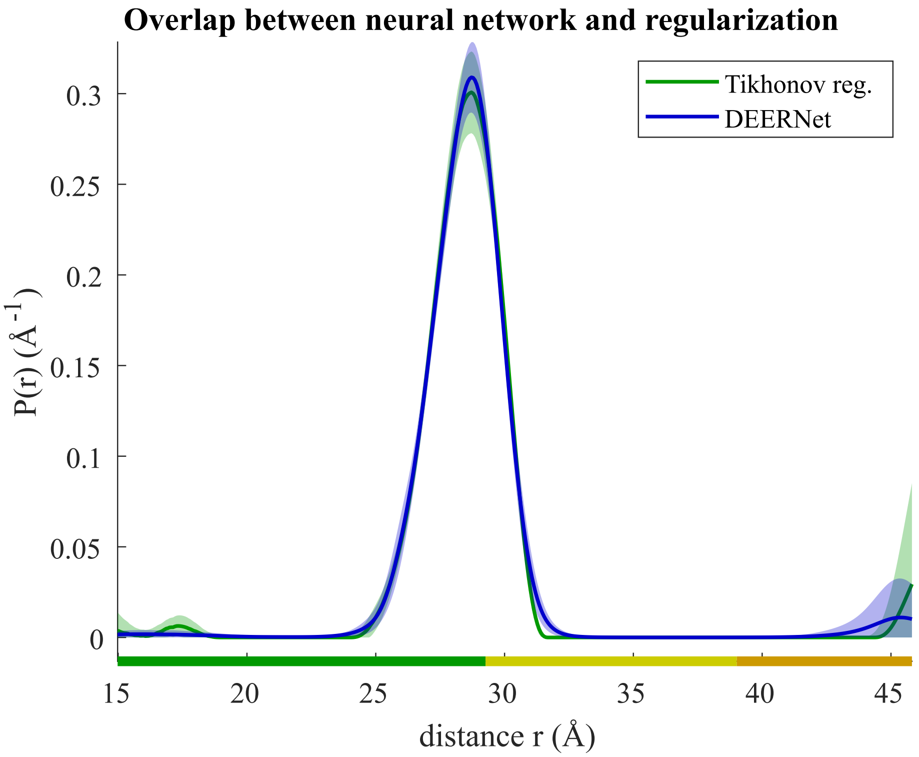


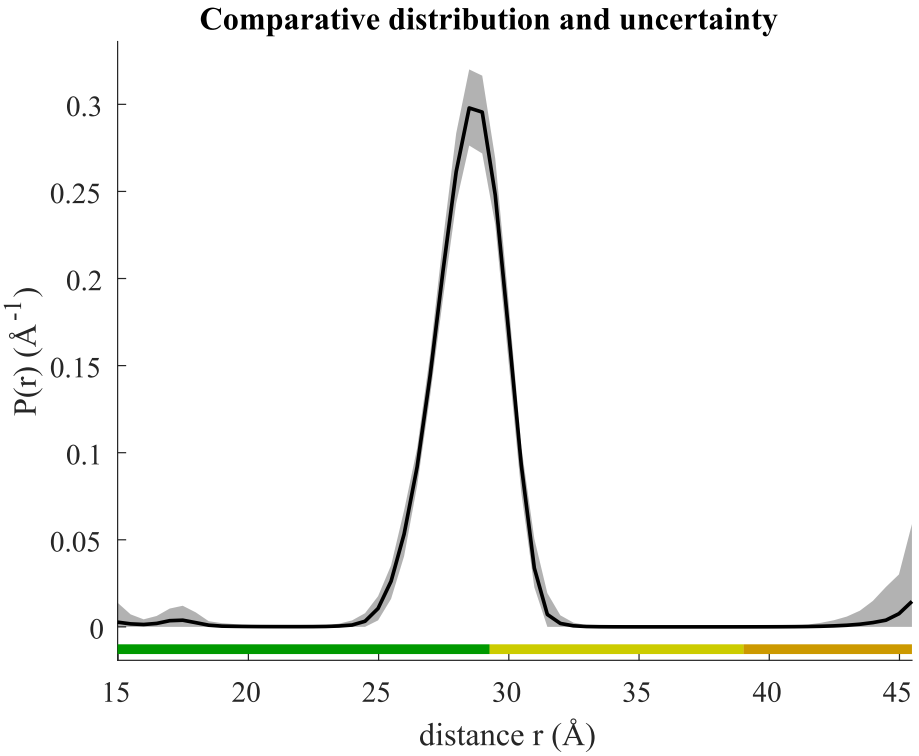

## 2. Fits of time-domain data

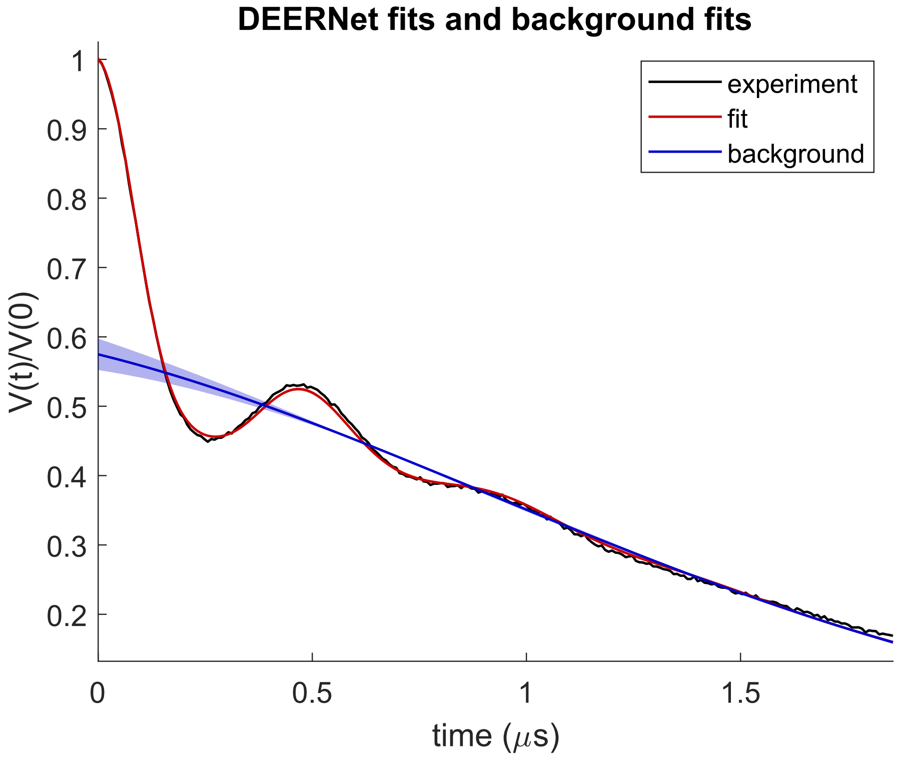


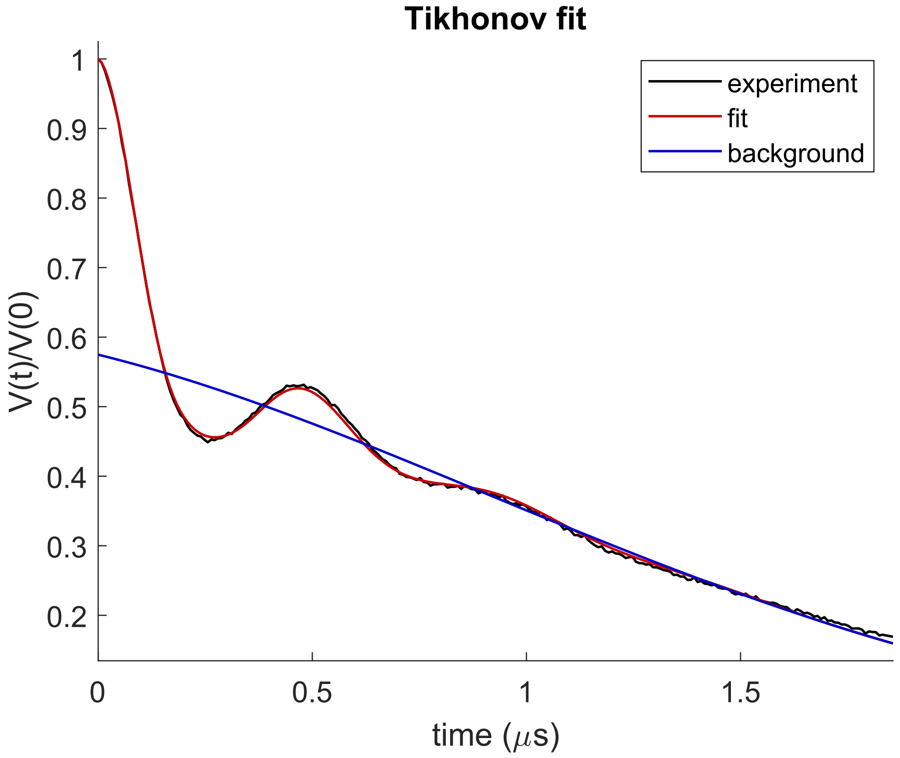

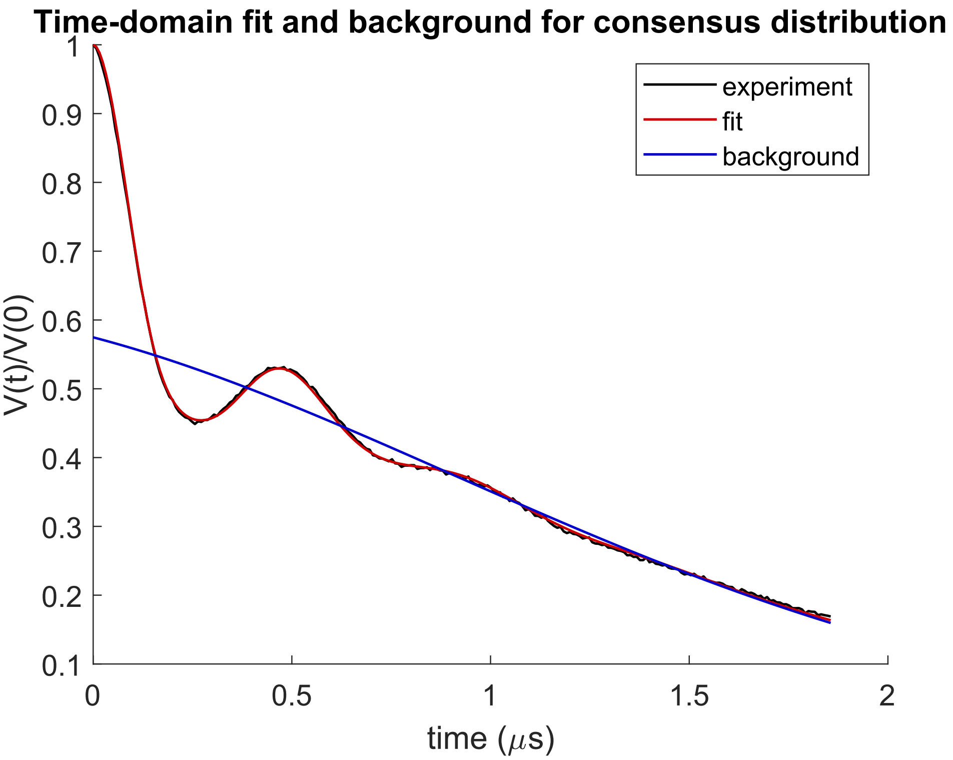
Time-domain fit and background for consensus distribution
experiment
fit
background
V(t)/V(0)
time ($\mu$s)
0
0.5
1
1.5
2
0.1
0.2
0.3
0.4
0.5
0.6
0.7
0.8
0.9
1

## 3. Experimental and processing parameters

**RIDME processing was requested. Only DEERNet output.**

Modulation depth: 0.425

Signal-to-noise ratio: 89.7 (w.r.t. modulation)

Noise estimates normalized to maximum signal

From imaginary part: 0.00285

From DEERNet fit: 0.00474

From Tikhonov fit: 0.00336

Zero time: 6 ns

Maximum time: 1856 ns

Time increment: 8 ns

Phase: -0.1 degree

Ensemble of 32 neural networks

Background separation by neural network

Background dimension: 3

Regularization parameter by best overlap with neural network solution

Regularization parameter used: 0.50

Reg. par. initial estimate by L-curve corner: 2.00

Overlap between DEERNet and regularization solutions: 0.966

Predicted overlap of consensus solution with ground truth: 0.83...1.00

Mean distance: 28.5 Å

Distance standard deviation: 1.3 Å

Full data set in Matlab format:
C:\Users\Katrin\Desktop\BEBQ78_500nM_vt\230413_78.60_vtctRIDME_vt_comparative_DEER_analysis.mat

Distance distributions in text format:
C:\Users\Katrin\Desktop\BEBQ78_500nM_vt\230413_78.60_vtctRIDME_vt_consensus_DEER_distribution.csv

Fit and background in text format:
C:\Users\Katrin\Desktop\BEBQ78_500nM_vt\230413_78.60_vtctRIDME_vt_consensus_DEER_fit.csv

Metadata:
C:\Users\Katrin\Desktop\BEBQ78_500nM_vt\230413_78.60_vtctRIDME_vt_comparative_DEER_meta_data.csv

# DEER analysis report on dataset 230413_78.60_vtctRIDME_vt_deconv

## DEERNet Spinach SVN Rev 5662 and DeerLab 0.9.1 Tikhonov regularization

### ComparativeDEERAnalyzer version 2.0

see: S. G. Worswick et al., DOI: 10.1126/sciadv.aat5218, L. Fabregas Ibanez et al., DOI: 10.5194/mr-1-209-2020

12-Sep-2023 09:55:21

# 1. Distance distributions

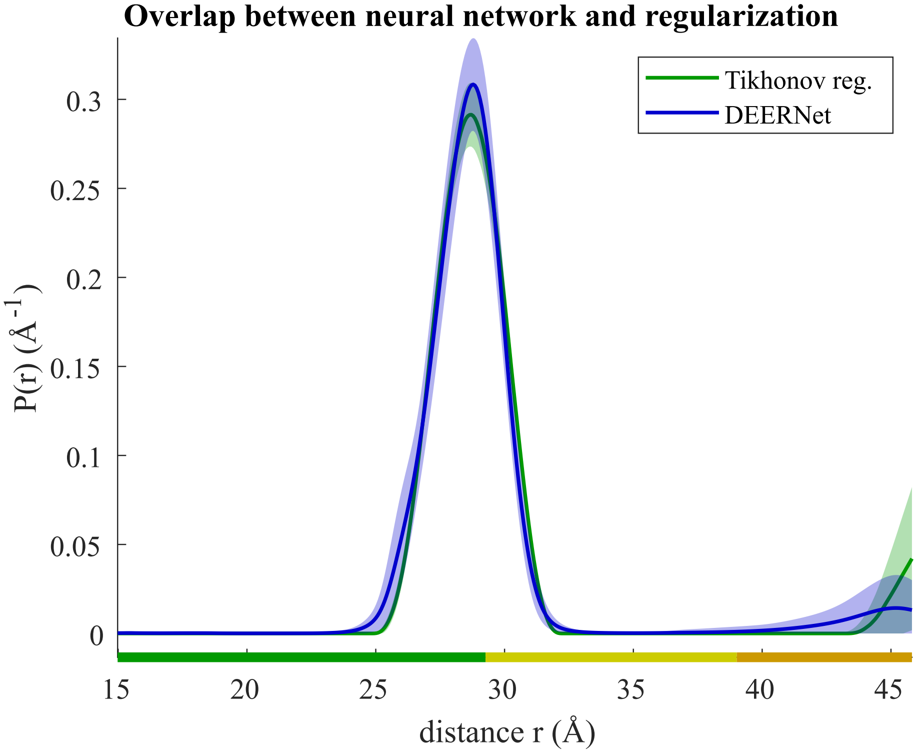


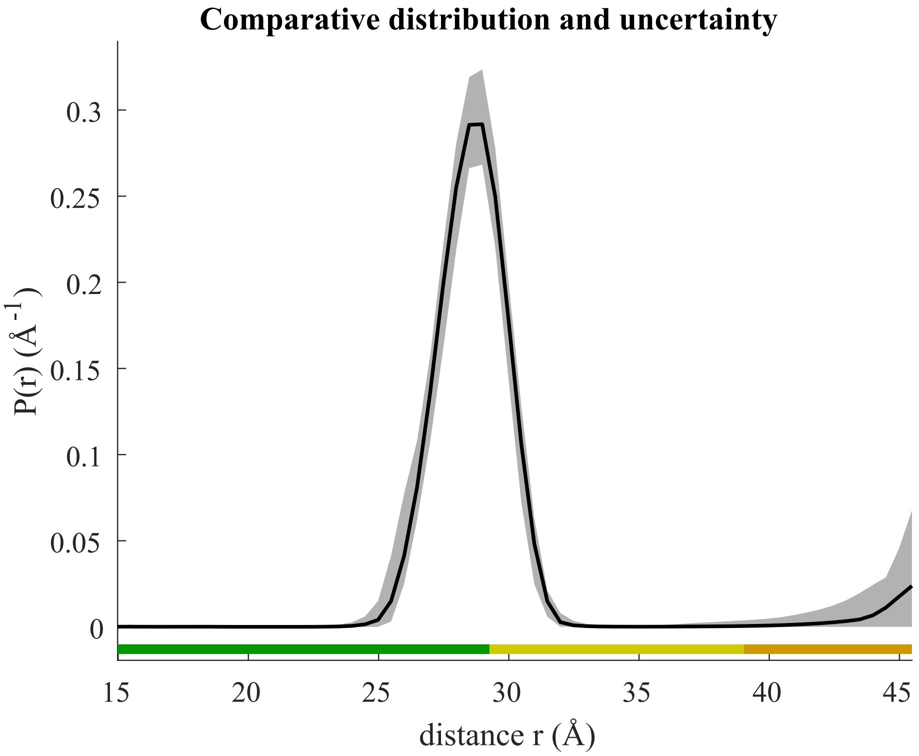

## 2. Fits of time-domain data

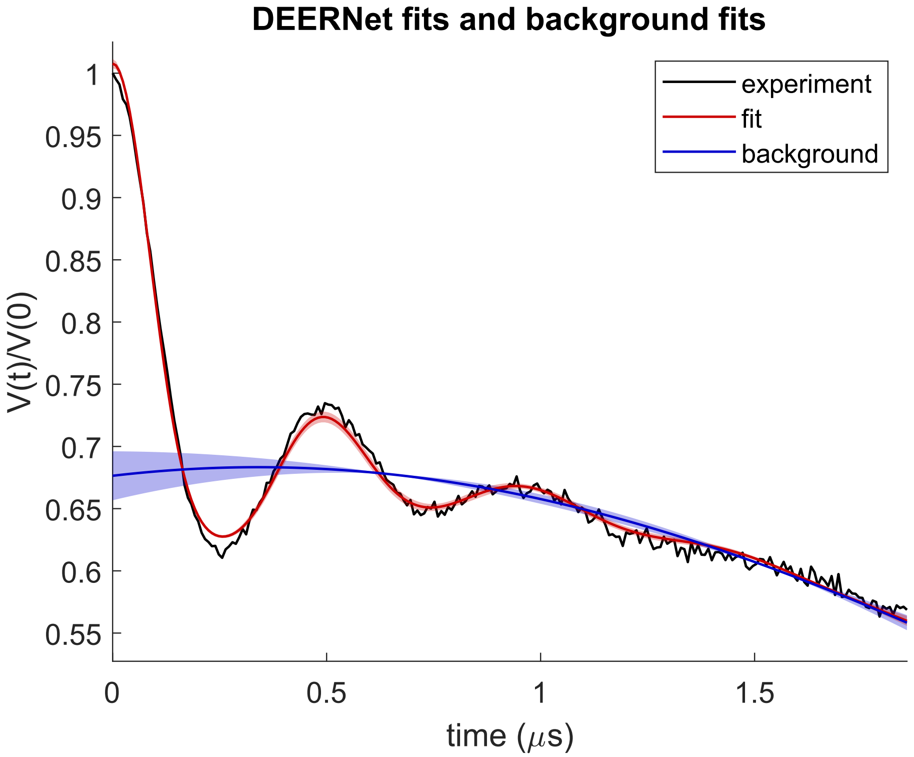


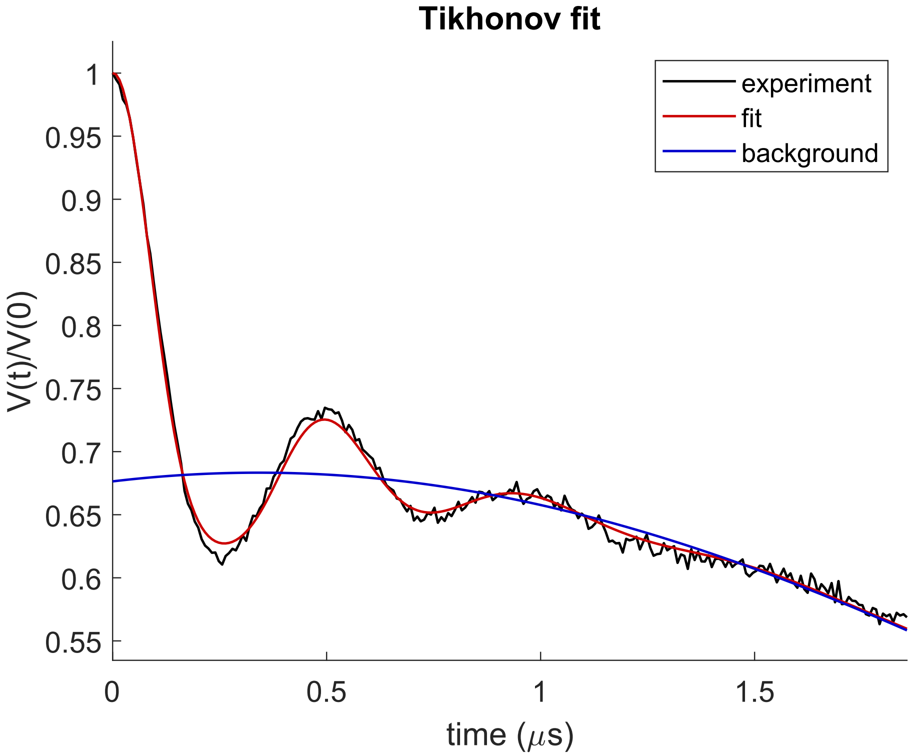

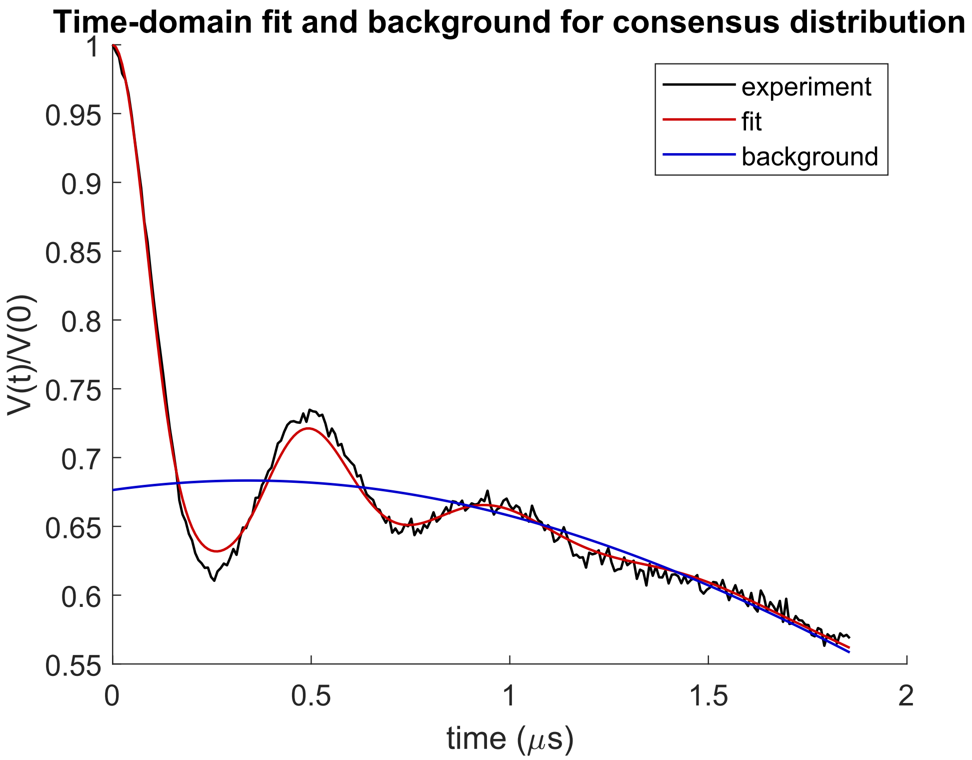
Time-domain fit and background for consensus distribution
experiment
fit
background
V(t)/V(0)
1
0.95
0.9
0.85
0.8
0.75
0.7
0.65
0.6
0.55
0
0.5
1
1.5
2
time (μs)

## 3. Experimental and processing parameters

**RIDME processing was requested. Only DEERNet output.**

Modulation depth: 0.329

Signal-to-noise ratio: 50.1 (w.r.t. modulation)

Noise estimates normalized to maximum signal

From imaginary part: 0.00451

From DEERNet fit: 0.00656

From Tikhonov fit: 0.00692

Zero time: 8 ns

Maximum time: 1856 ns

Time increment: 8 ns

Phase: -0.0 degree

Ensemble of 32 neural networks

Background separation by neural network

Background dimension: 3

Regularization parameter by best overlap with neural network solution

Regularization parameter used: 1.41

Reg. par. initial estimate by L-curve corner: 2.00

Overlap between DEERNet and regularization solutions: 0.933

Predicted overlap of consensus solution with ground truth: 0.80...0.97

Mean distance: 28.7 Å

Distance standard deviation: 1.1 Å

Full data set in Matlab format:
C:\Users\Katrin\Desktop\BEBQ78_500nM_vt_deconv\230413_78.60_vtctRIDME_vt_deconv_comparative_DEER_analysis.mat

Distance distributions in text format:
C:\Users\Katrin\Desktop\BEBQ78_500nM_vt_deconv\230413_78.60_vtctRIDME_vt_deconv_consensus_DEER_distribution.csv

Fit and background in text format:
C:\Users\Katrin\Desktop\BEBQ78_500nM_vt_deconv\230413_78.60_vtctRIDME_vt_deconv_consensus_DEER_fit.csv

Metadata:
C:\Users\Katrin\Desktop\BEBQ78_500nM_vt_deconv\230413_78.60_vtctRIDME_vt_deconv_comparative_DEER_meta_data.csv

# DEER analysis report on dataset 2330414_KAq213.2_vtctRIDME_5_60_ct

## DEERNet Spinach SVN Rev 5662 and DeerLab 0.9.1 Tikhonov regularization

### ComparativeDEERAnalyzer version 2.0

see: S. G. Worswick et al., DOI: 10.1126/sciadv.aat5218, L. Fabregas Ibanez et al., DOI: 10.5194/mr-1-209-2020

12-Sep-2023 10:16:34

## 1. Distance distributions

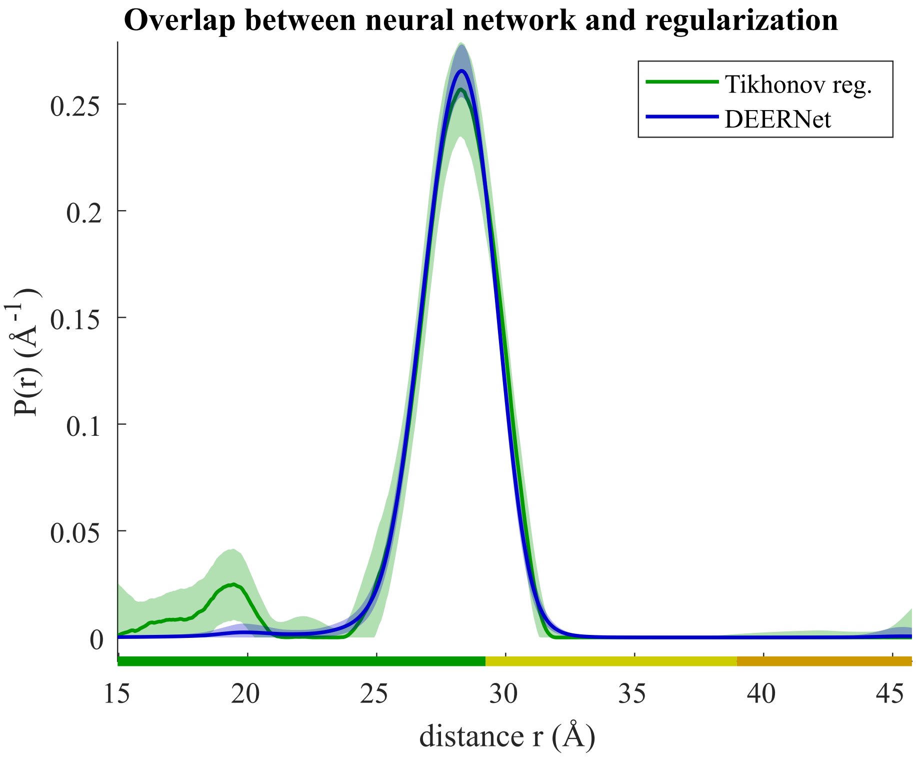


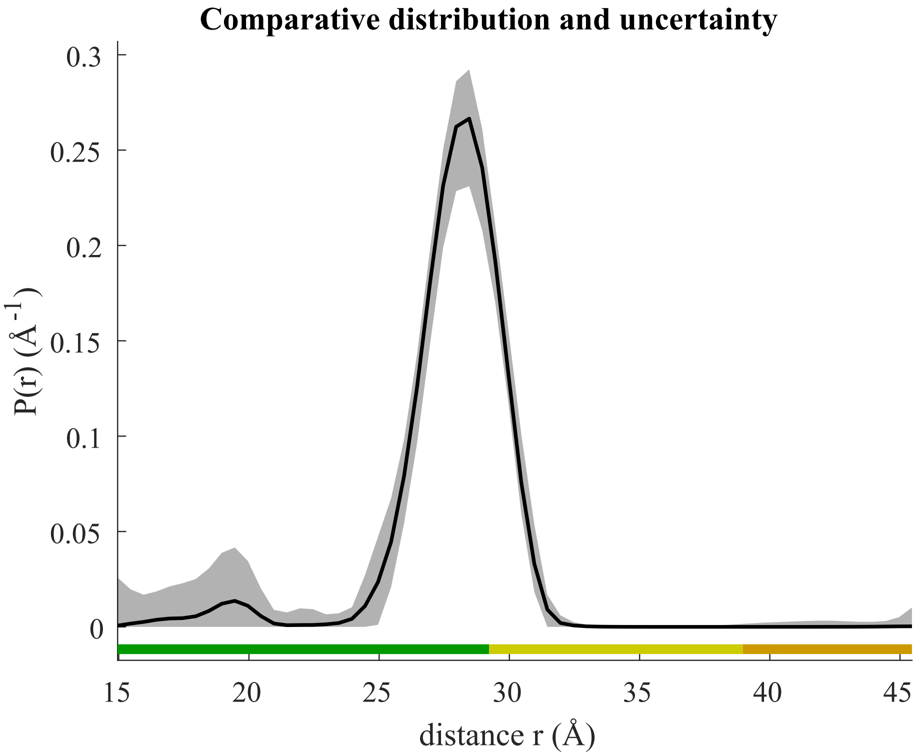

## 2. Fits of time-domain data

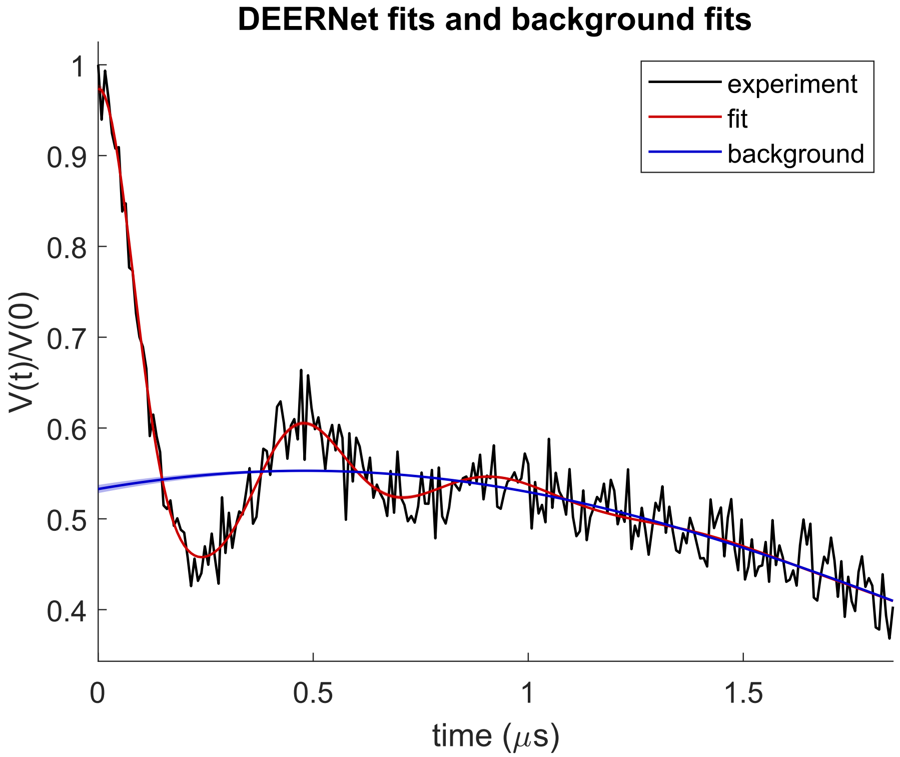


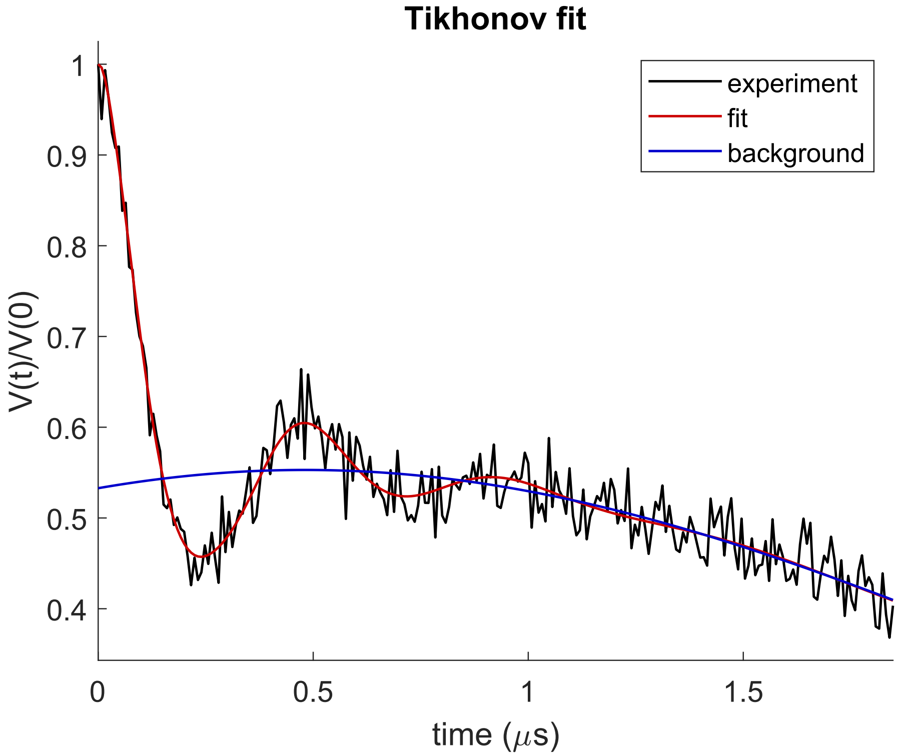

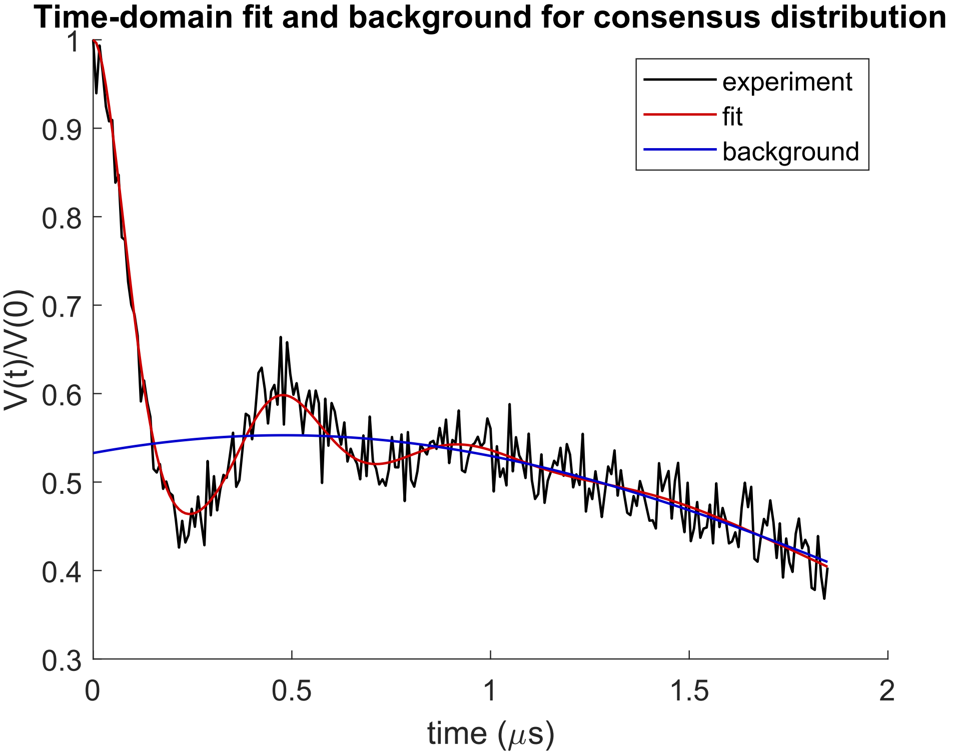
Time-domain fit and background for consensus distribution
experiment
fit
background
V(t)/V(0)
time (μs)
0
0.5
1
1.5
2
0.3
0.4
0.5
0.6
0.7
0.8
0.9
1

## 3. Experimental and processing parameters

**RIDME processing was requested. Only DEERNet output.**

**Please consider improving signal-to-noise ratio (below 20)**

Modulation depth: 0.453

Signal-to-noise ratio: 18.4 (w.r.t. modulation)

Noise estimates normalized to maximum signal

From imaginary part: 0.02513

From DEERNet fit: 0.02456

From Tikhonov fit: 0.02495

Zero time: 13 ns

Maximum time: 1848 ns

Time increment: 8 ns

Phase: -0.0 degree

Ensemble of 32 neural networks

Background separation by neural network

Background dimension: 3

Regularization parameter by best overlap with neural network solution

Regularization parameter used: 1.40

Reg. par. initial estimate by L-curve corner: 7.94

Overlap between DEERNet and regularization solutions: 0.924

Predicted overlap of consensus solution with ground truth: 0.79...0.96

Mean distance: 28.1 Å

Distance standard deviation: 1.4 Å

Full data set in Matlab format:
C:\Users\Katrin\Desktop\213_2_100nM_ct\2330414_KAq213.2_vtctRIDME_5_60_ct_comparative_DEER_analysis.mat

Distance distributions in text format:
C:\Users\Katrin\Desktop\213_2_100nM_ct\2330414_KAq213.2_vtctRIDME_5_60_ct_consensus_DEER_distribution.csv

Fit and background in text format:
C:\Users\Katrin\Desktop\213_2_100nM_ct\2330414_KAq213.2_vtctRIDME_5_60_ct_consensus_DEER_fit.csv

Metadata:
C:\Users\Katrin\Desktop\213_2_100nM_ct\2330414_KAq213.2_vtctRIDME_5_60_ct_comparative_DEER_meta_data.csv

# DEER analysis report on dataset 2330414_KAq213.2_vtctRIDME_5_60_ct_deconv

## DEERNet Spinach SVN Rev 5662 and DeerLab 0.9.1 Tikhonov regularization

### ComparativeDEERAnalyzer version 2.0

see: S. G. Worswick et al., DOI: 10.1126/sciadv.aat5218, L. Fabregas Ibanez et al., DOI: 10.5194/mr-1-209-2020

12-Sep-2023 10:38:14

## 1. Distance distributions

**Overlap between neural network and regularization**

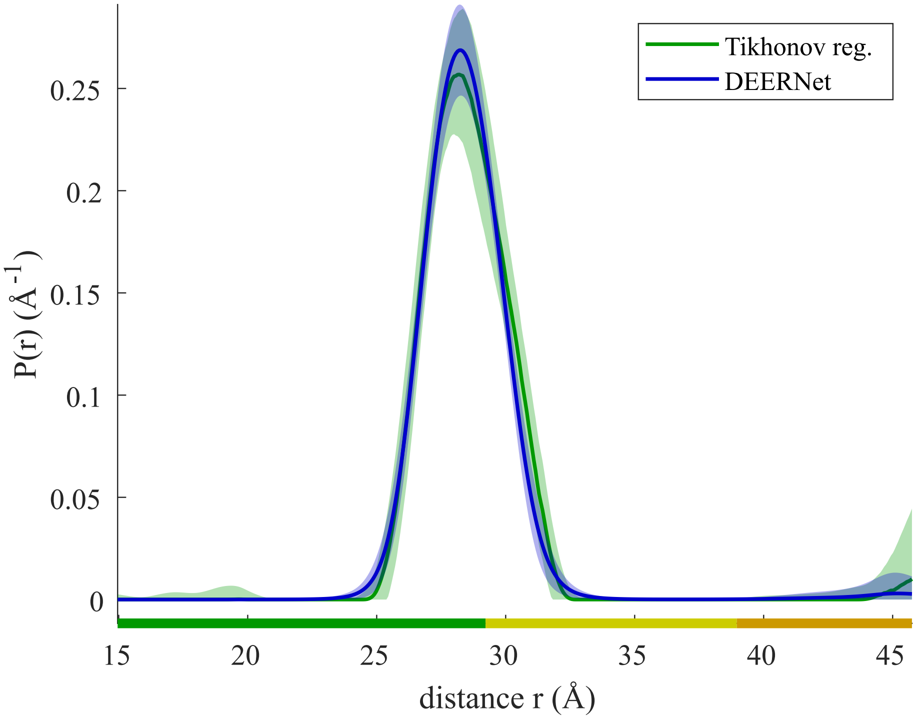


**Comparative distribution and uncertainty**

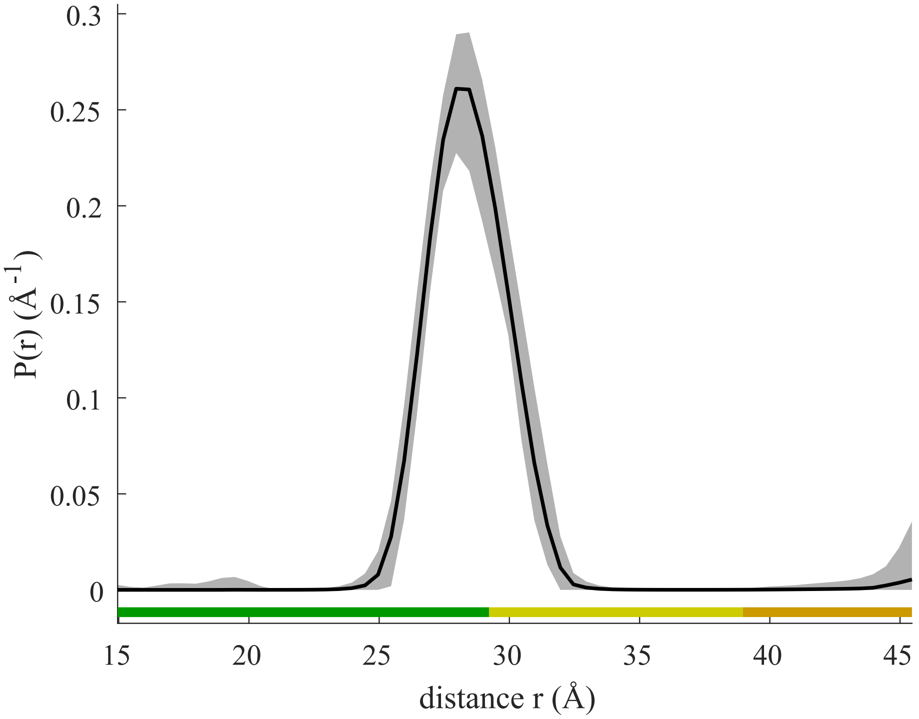

## 2. Fits of time-domain data

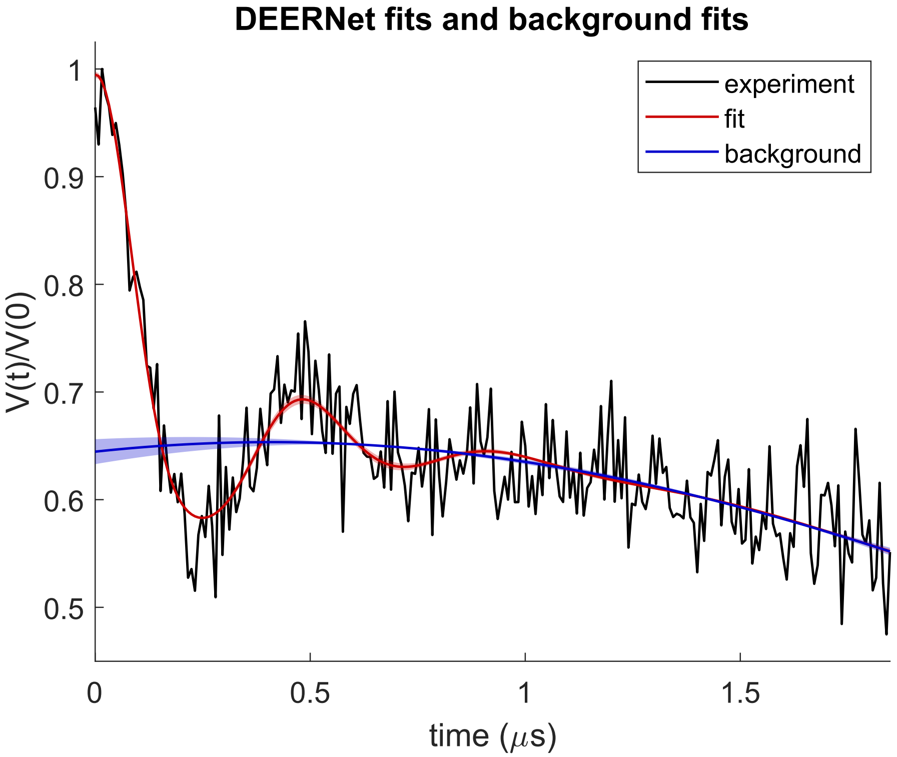


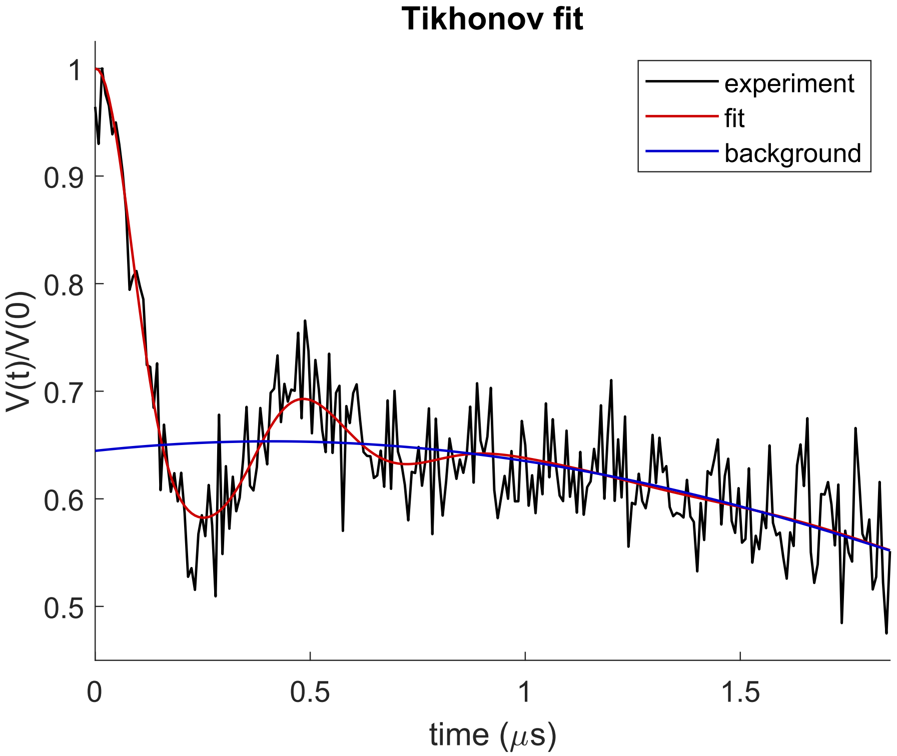

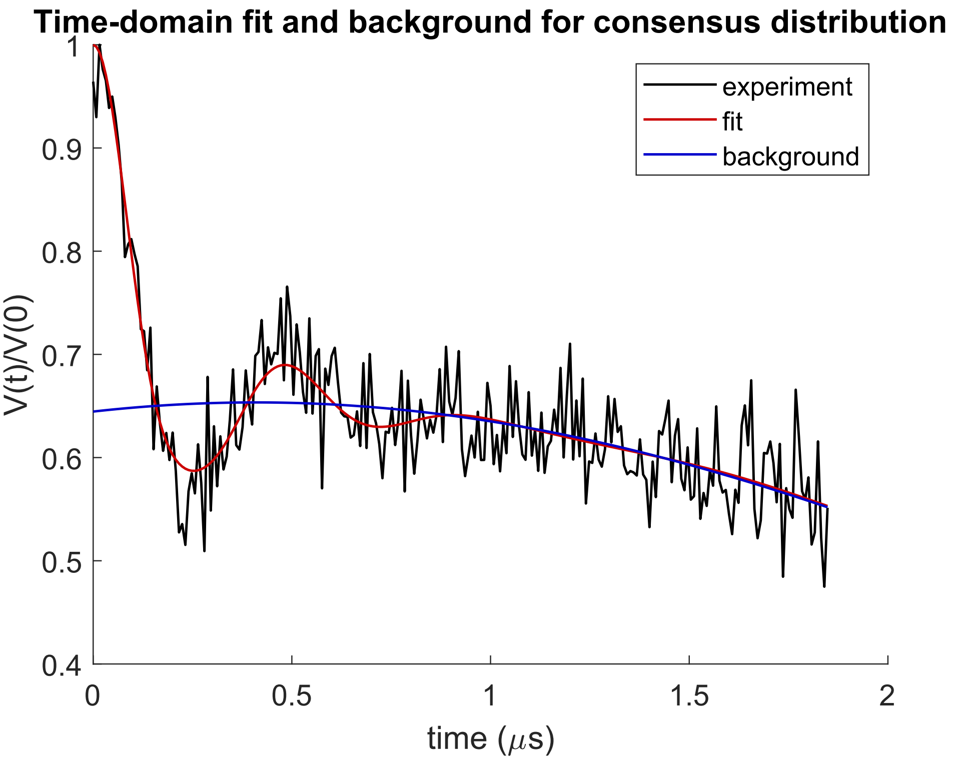
Time-domain fit and background for consensus distribution
experiment
fit
background
V(t)/V(0)
time ($\mu$s)
1
0.9
0.8
0.7
0.6
0.5
0.4
0
0.5
1
1.5
2

## 3. Experimental and processing parameters

**RIDME processing was requested. Only DEERNet output.**

**Use of distribution not recommended, since signal-to-noise ratio is below 10**

Modulation depth: 0.352

Signal-to-noise ratio: 9.7 (w.r.t. modulation)

Noise estimates normalized to maximum signal

From imaginary part: 0.03652

From DEERNet fit: 0.03628

From Tikhonov fit: 0.03638

Zero time: 15 ns

Maximum time: 1848 ns

Time increment: 8 ns

Phase: -0.2 degree

Ensemble of 32 neural networks

Background separation by neural network

Background dimension: 3

Regularization parameter by best overlap with neural network solution

Regularization parameter used: 1.99

Reg. par. initial estimate by L-curve corner: 7.94

Overlap between DEERNet and regularization solutions: 0.953

Predicted overlap of consensus solution with ground truth: 0.82...0.99

Mean distance: 28.4 Å

Distance standard deviation: 1.3 Å

Full data set in Matlab format:
C:\Users\Katrin\Desktop\213_2_100nM_ct_deconv\2330414_KAq213.2_vtctRIDME_5_60_ct_deconv_comparative_DEER_analysis.mat

Distance distributions in text format:
C:\Users\Katrin\Desktop\213_2_100nM_ct_deconv\2330414_KAq213.2_vtctRIDME_5_60_ct_deconv_consensus_DEER_distribution.csv

Fit and background in text format:
C:\Users\Katrin\Desktop\213_2_100nM_ct_deconv\2330414_KAq213.2_vtctRIDME_5_60_ct_deconv_consensus_DEER_fit.csv

Metadata:
C:\Users\Katrin\Desktop\213_2_100nM_ct_deconv\2330414_KAq213.2_vtctRIDME_5_60_ct_deconv_comparative_DEER_meta_data.csv

# DEER analysis report on dataset 2330414_KAq213.2_vtctRIDME_5_60_vt

## DEERNet Spinach SVN Rev 5662 and DeerLab 0.9.1 Tikhonov regularization

### ComparativeDEERAnalyzer version 2.0

see: S. G. Worswick et al., DOI: 10.1126/sciadv.aat5218, L. Fabregas Ibanez et al., DOI: 10.5194/mr-1-209-2020

12-Sep-2023 10:56:21

## 1. Distance distributions

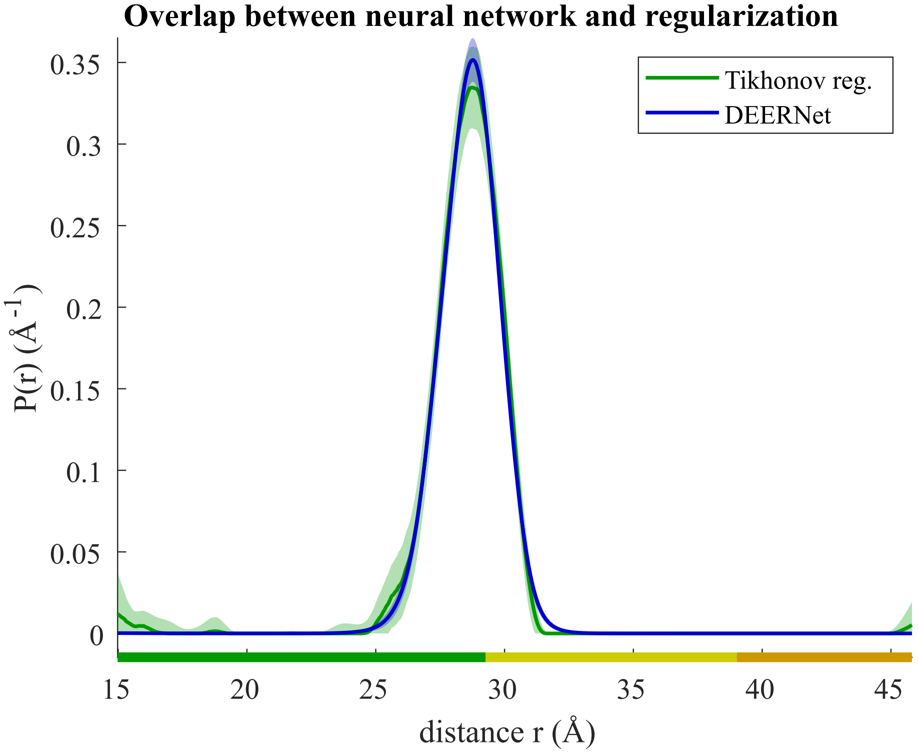


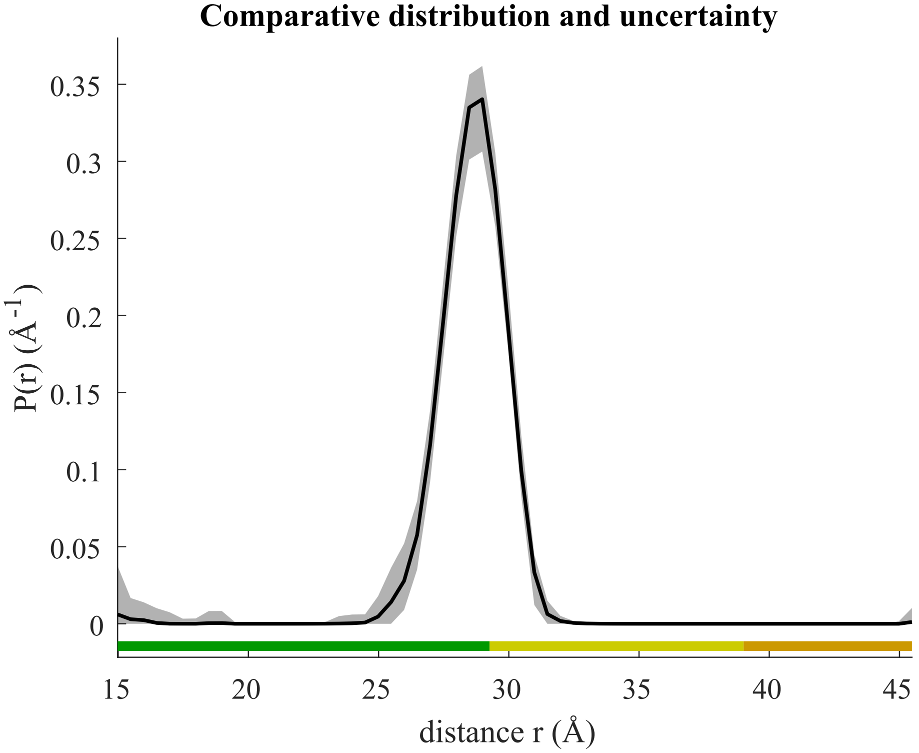

## 2. Fits of time-domain data

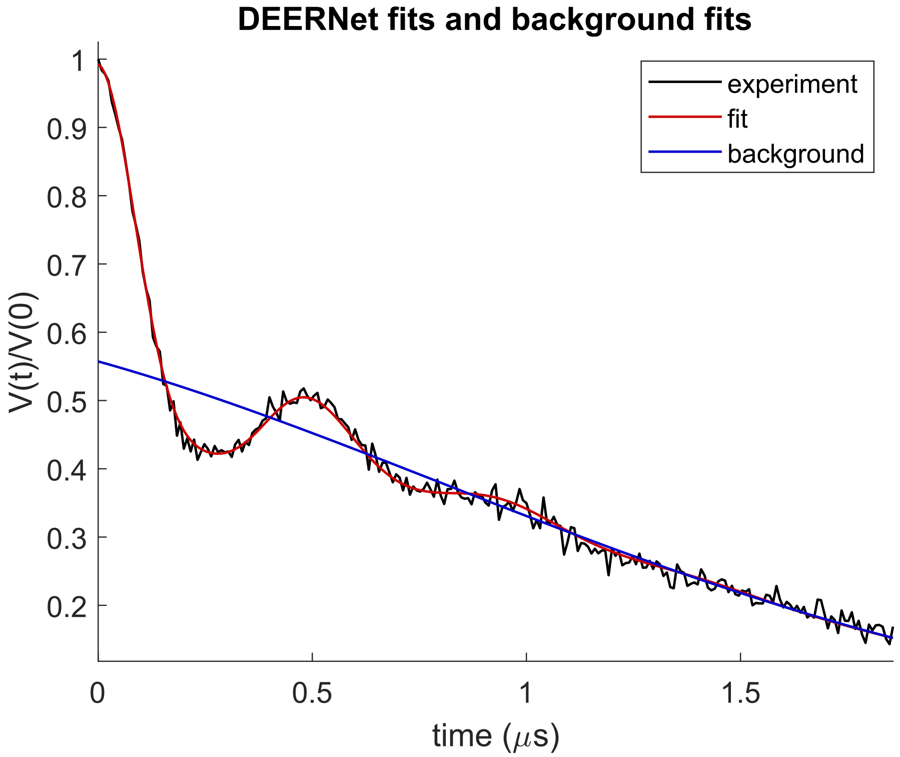


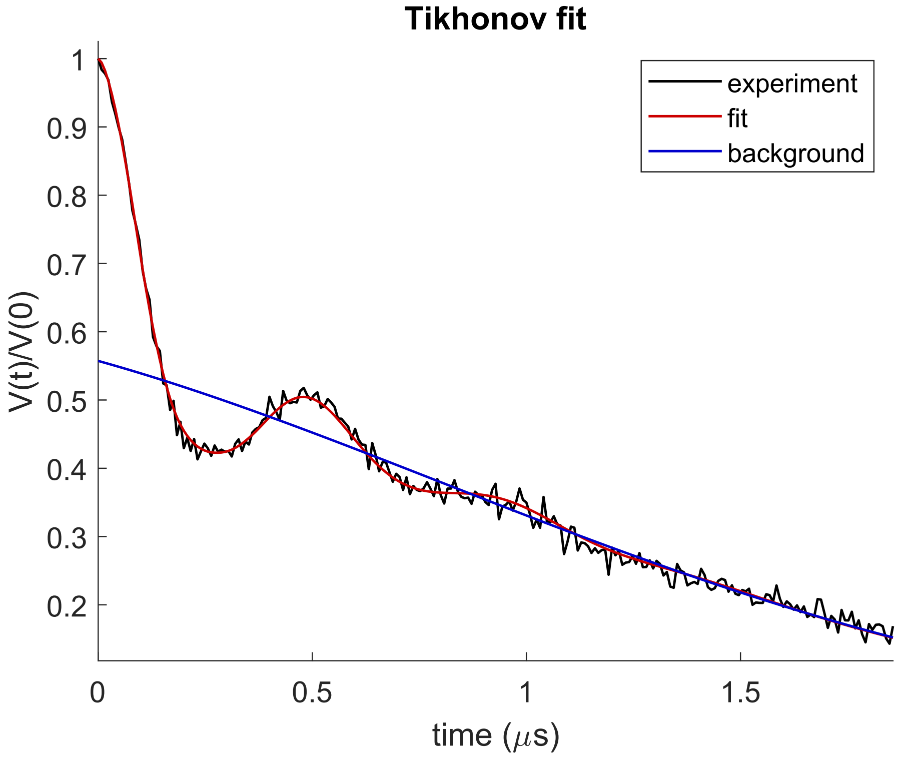

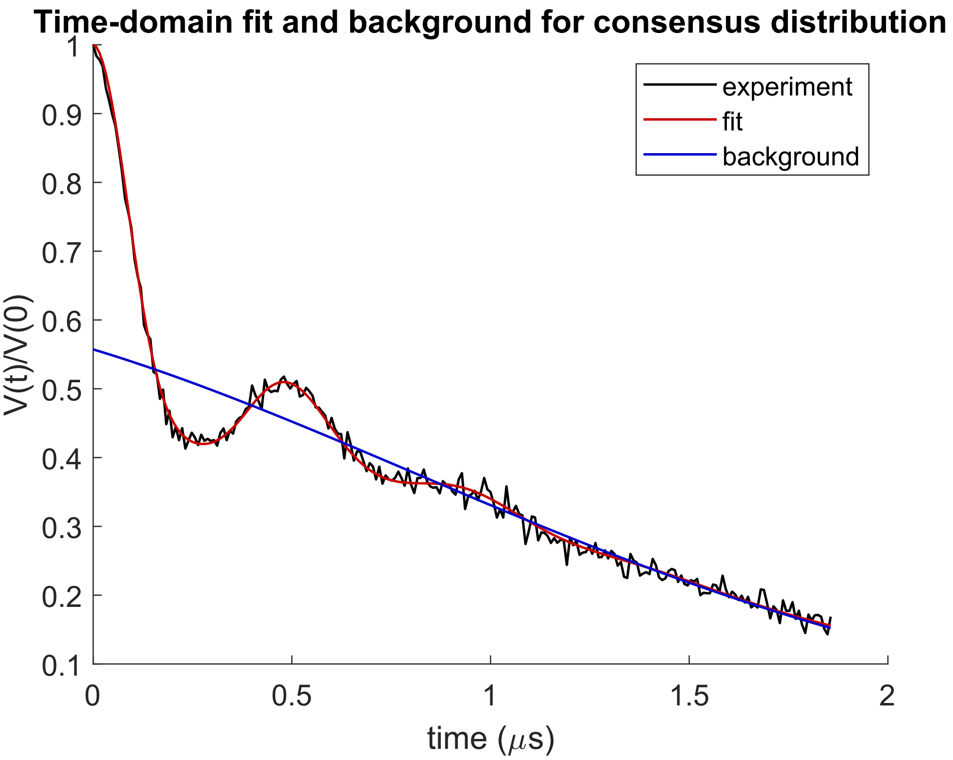
Time-domain fit and background for consensus distribution
experiment
fit
background
V(t)/V(0)
1
0.9
0.8
0.7
0.6
0.5
0.4
0.3
0.2
0.1
0
0.5
1
1.5
2
time (μs)

## 3. Experimental and processing parameters

**RIDME processing was requested. Only DEERNet output.**

Modulation depth: 0.439

Signal-to-noise ratio: 38.0 (w.r.t. modulation)

Noise estimates normalized to maximum signal

From imaginary part: 0.01188

From DEERNet fit: 0.01155

From Tikhonov fit: 0.01126

Zero time: 7 ns

Maximum time: 1856 ns

Time increment: 8 ns

Phase: 0.2 degree

Ensemble of 32 neural networks

Background separation by neural network

Background dimension: 3

Regularization parameter by best overlap with neural network solution

Regularization parameter used: 0.35

Reg. par. initial estimate by L-curve corner: 3.98

Overlap between DEERNet and regularization solutions: 0.967

Predicted overlap of consensus solution with ground truth: 0.83...1.00

Mean distance: 28.6 Å

Distance standard deviation: 1.2 Å

Full data set in Matlab format:
C:\Users\Katrin\Desktop\213_2_100nM_vt\2330414_KAq213.2_vtctRIDME_5_60_vt_comparative_DEER_analysis.mat

Distance distributions in text format:
C:\Users\Katrin\Desktop\213_2_100nM_vt\2330414_KAq213.2_vtctRIDME_5_60_vt_consensus_DEER_distribution.csv

Fit and background in text format:
C:\Users\Katrin\Desktop\213_2_100nM_vt\2330414_KAq213.2_vtctRIDME_5_60_vt_consensus_DEER_fit.csv

Metadata:
C:\Users\Katrin\Desktop\213_2_100nM_vt\2330414_KAq213.2_vtctRIDME_5_60_vt_comparative_DEER_meta_data.csv

# DEER analysis report on dataset 2330414_KAq213.2_vtctRIDME_5_60_vt_deconv

## DEERNet Spinach SVN Rev 5662 and DeerLab 0.9.1 Tikhonov regularization

### ComparativeDEERAnalyzer version 2.0

see: S. G. Worswick et al., DOI: 10.1126/sciadv.aat5218, L. Fabregas Ibanez et al., DOI: 10.5194/mr-1-209-2020

12-Sep-2023 11:14:48

# 1. Distance distributions

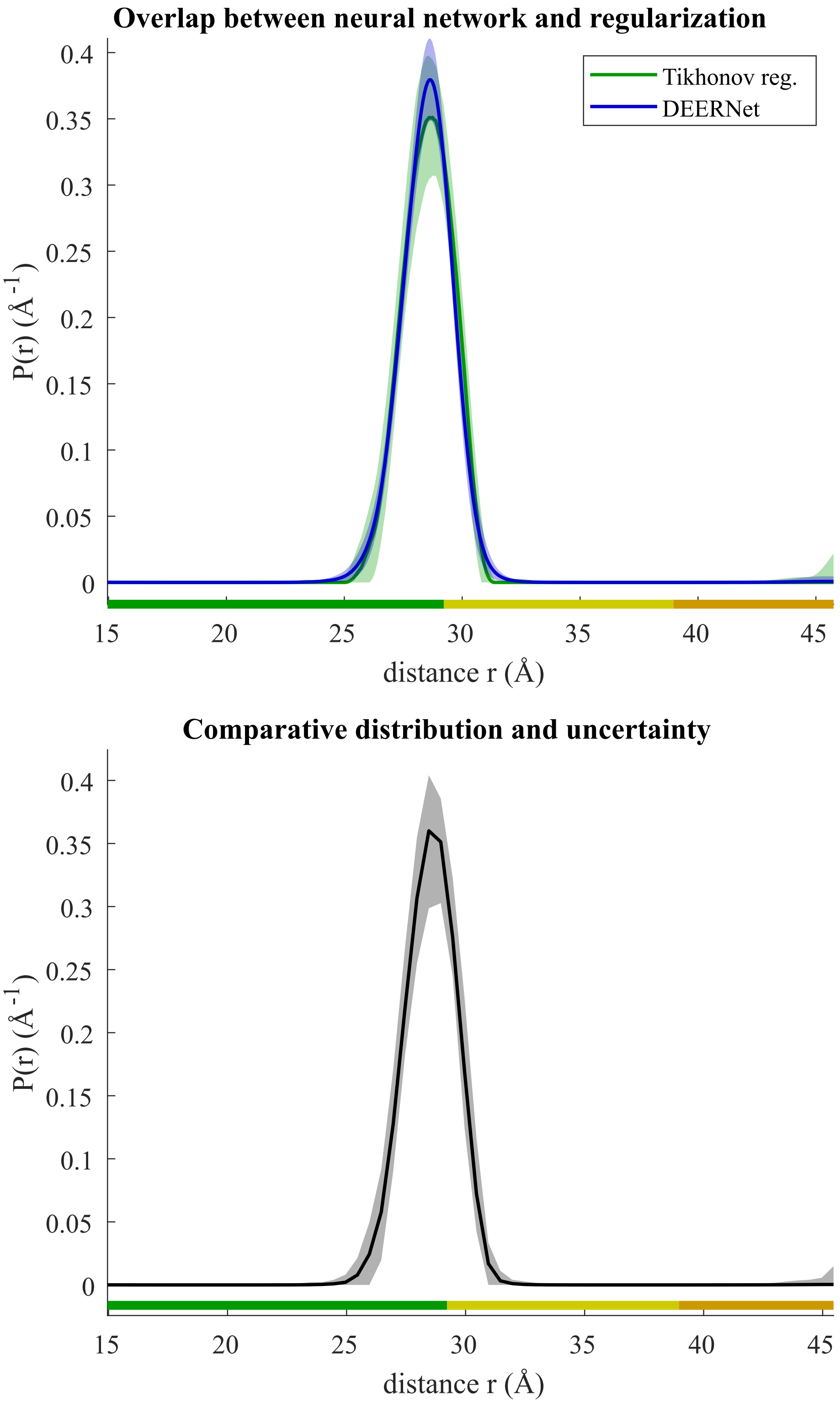

## 2. Fits of time-domain data

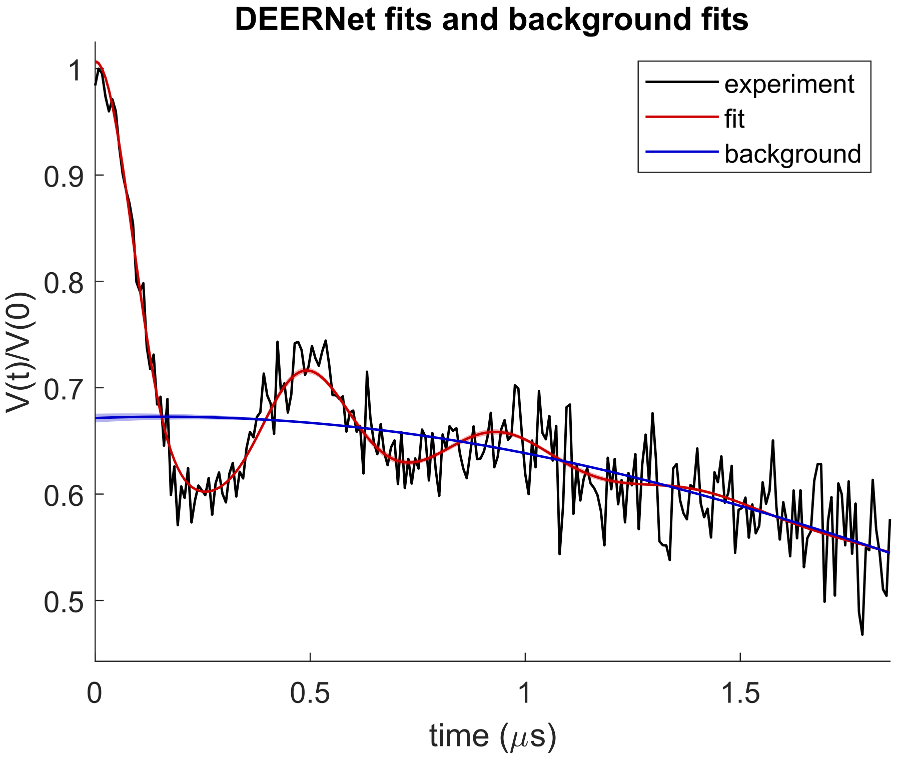


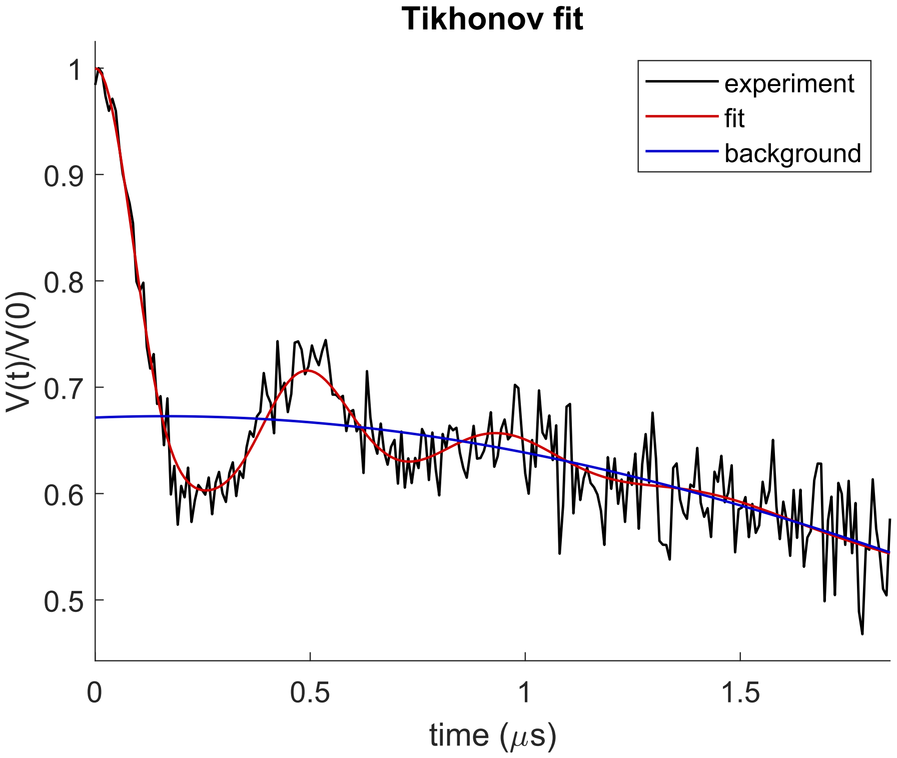

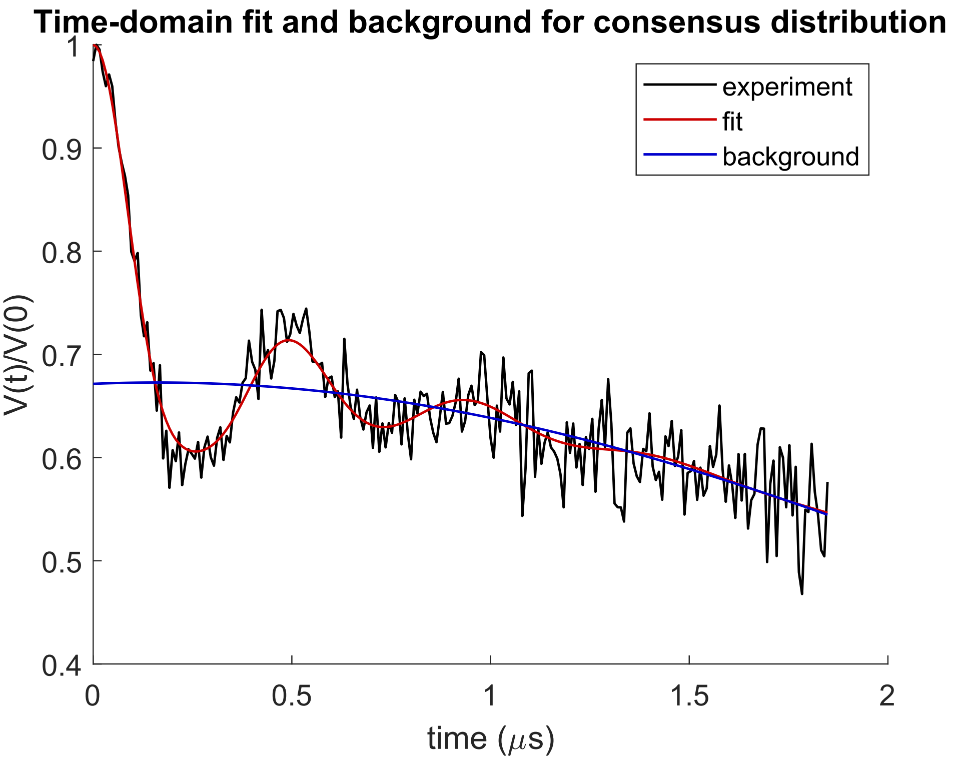
Time-domain fit and background for consensus distribution
experiment
fit
background
V(t)/V(0)
1
0.9
0.8
0.7
0.6
0.5
0.4
0
0.5
1
1.5
2
time (μs)

## 3. Experimental and processing parameters

**RIDME processing was requested. Only DEERNet output.**

**Please consider improving signal-to-noise ratio (below 20)**

Modulation depth: 0.333

Signal-to-noise ratio: 11.7 (w.r.t. modulation)

Noise estimates normalized to maximum signal

From imaginary part: 0.03021

From DEERNet fit: 0.02840

From Tikhonov fit: 0.02841

Zero time: 13 ns

Maximum time: 1848 ns

Time increment: 8 ns

Phase: 0.3 degree

Ensemble of 32 neural networks

Background separation by neural network

Background dimension: 3

Regularization parameter by best overlap with neural network solution

Regularization parameter used: 0.56

Reg. par. initial estimate by L-curve corner: 6.31

Overlap between DEERNet and regularization solutions: 0.956

Predicted overlap of consensus solution with ground truth: 0.82...0.99

Mean distance: 28.6 Å

Distance standard deviation: 1.0 Å

Full data set in Matlab format: C:\Users\Katrin\Desktop\213_2_100nM_vt_deconv\2330414_KAq213.2_vtctRIDME_5_60_vt_deconv_comparative_DEER_analysis.mat

Distance distributions in text format: C:\Users\Katrin\Desktop\213_2_100nM_vt_deconv\2330414_KAq213.2_vtctRIDME_5_60_vt_deconv_consensus_DEER_distribution.csv

Fit and background in text format:
C:\Users\Katrin\Desktop\213_2_100nM_vt_deconv\2330414_KAq213.2_vtctRIDME_5_60_vt_deconv_consensus_DEER_fit.csv

Metadata:
C:\Users\Katrin\Desktop\213_2_100nM_vt_deconv\2330414_KAq213.2_vtctRIDME_5_60_vt_deconv_comparative_DEER_meta_data.csv

# DEER analysis report on dataset 2330417_KAq213.4_vtctRIDME_60_1

## DEERNet Spinach SVN Rev 5662 and DeerLab 0.9.1 Tikhonov regularization

### ComparativeDEERAnalyzer version 2.0

see: S. G. Worswick et al., DOI: 10.1126/sciadv.aat5218, L. Fabregas Ibanez et al., DOI: 10.5194/mr-1-209-2020

12-Sep-2023 12:47:43

## 1. Distance distributions

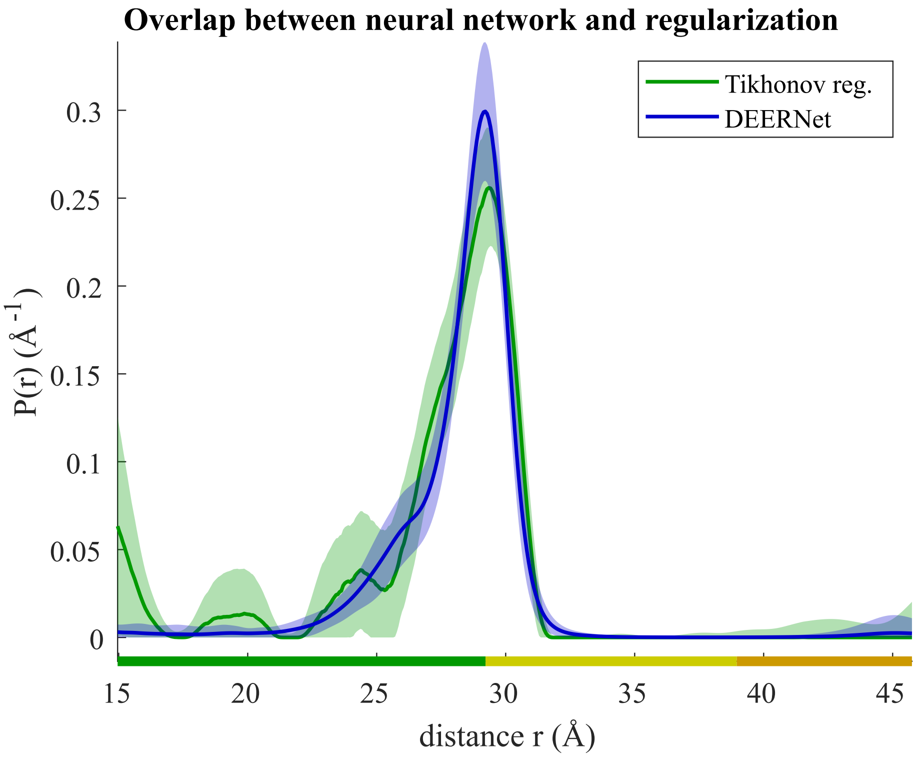


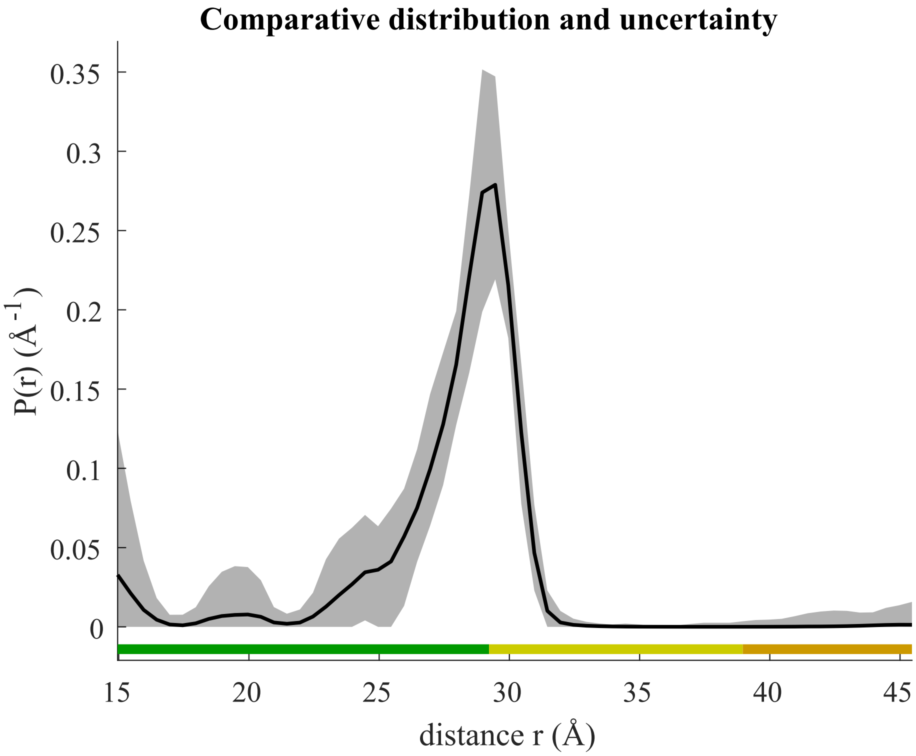

## 2. Fits of time-domain data

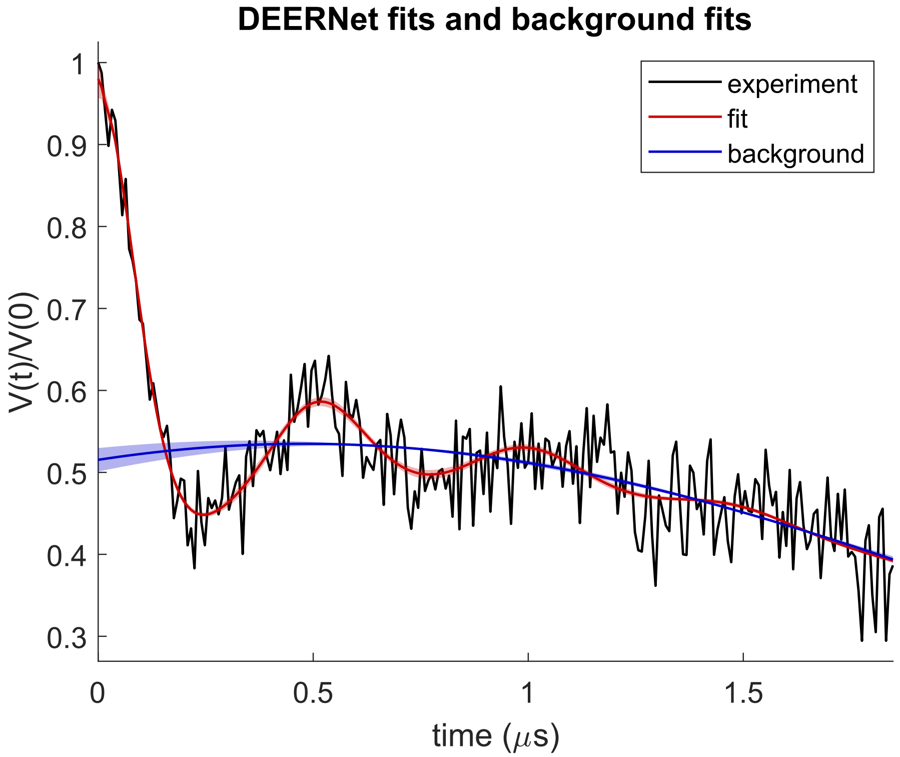


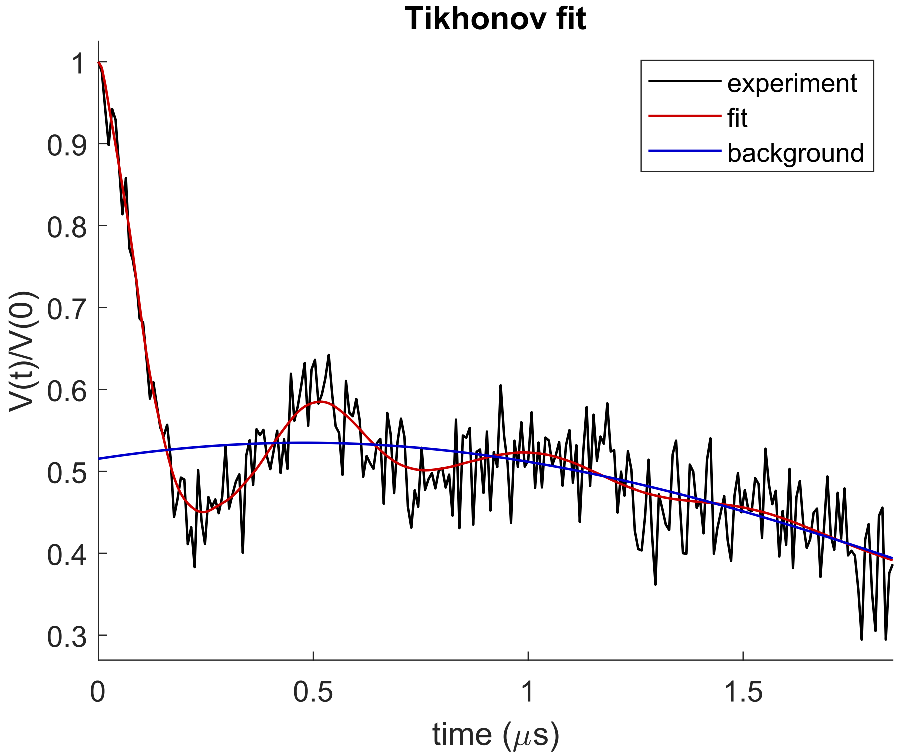

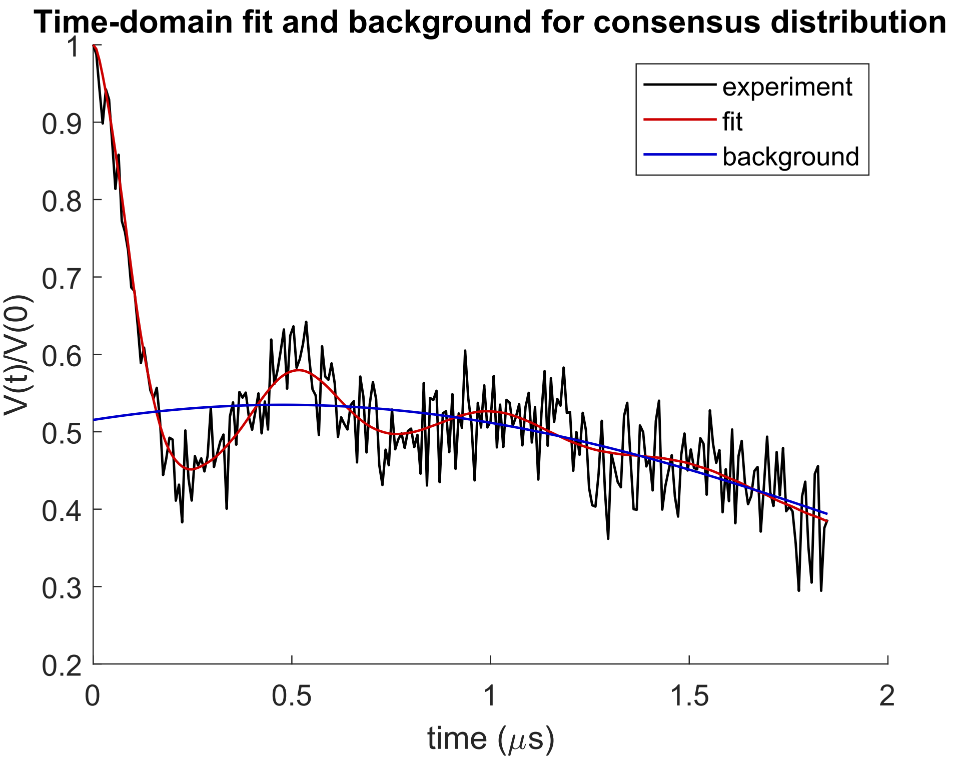
Time-domain fit and background for consensus distribution
experiment
fit
background
V(t)/V(0)
time (μs)
0
0.5
1
1.5
2
0.2
0.3
0.4
0.5
0.6
0.7
0.8
0.9
1

## 3. Experimental and processing parameters

**RIDME processing was requested. Only DEERNet output.**

**Please consider improving signal-to-noise ratio (below 20)**

Modulation depth: 0.474

Signal-to-noise ratio: 11.8 (w.r.t. modulation)

Noise estimates normalized to maximum signal

From imaginary part: 0.04384

From DEERNet fit: 0.04021

From Tikhonov fit: 0.04008

Zero time: 17 ns

Maximum time: 1848 ns

Time increment: 8 ns

Phase: -0.0 degree

Ensemble of 32 neural networks

Background separation by neural network

Background dimension: 3

Regularization parameter by best overlap with neural network solution

Regularization parameter used: 1.40

Reg. par. initial estimate by L-curve corner: 7.94

Overlap between DEERNet and regularization solutions: 0.863

Predicted overlap of consensus solution with ground truth: 0.73...0.91

Mean distance: 28.5 Å

Distance standard deviation: 1.5 Å

Full data set in Matlab format:
C:\Users\Katrin\Desktop\213_4_50nM_ct\2330417_KAq213.4_vtctRIDME_60_1_comparative_DEER_analysis.mat

Distance distributions in text format:
C:\Users\Katrin\Desktop\213_4_50nM_ct\2330417_KAq213.4_vtctRIDME_60_1_consensus_DEER_distribution.csv

Fit and background in text format:
C:\Users\Katrin\Desktop\213_4_50nM_ct\2330417_KAq213.4_vtctRIDME_60_1_consensus_DEER_fit.csv

Metadata:
C:\Users\Katrin\Desktop\213_4_50nM_ct\2330417_KAq213.4_vtctRIDME_60_1_comparative_DEER_meta_data.csv

# DEER analysis report on dataset 2330417_KAq213.4_vtctRIDME_60_0

## DEERNet Spinach SVN Rev 5662 and DeerLab 0.9.1 Tikhonov regularization

### ComparativeDEERAnalyzer version 2.0

see: S. G. Worswick et al., DOI: 10.1126/sciadv.aat5218, L. Fabregas Ibanez et al., DOI: 10.5194/mr-1-209-2020

12-Sep-2023 13:07:06

## 1. Distance distributions

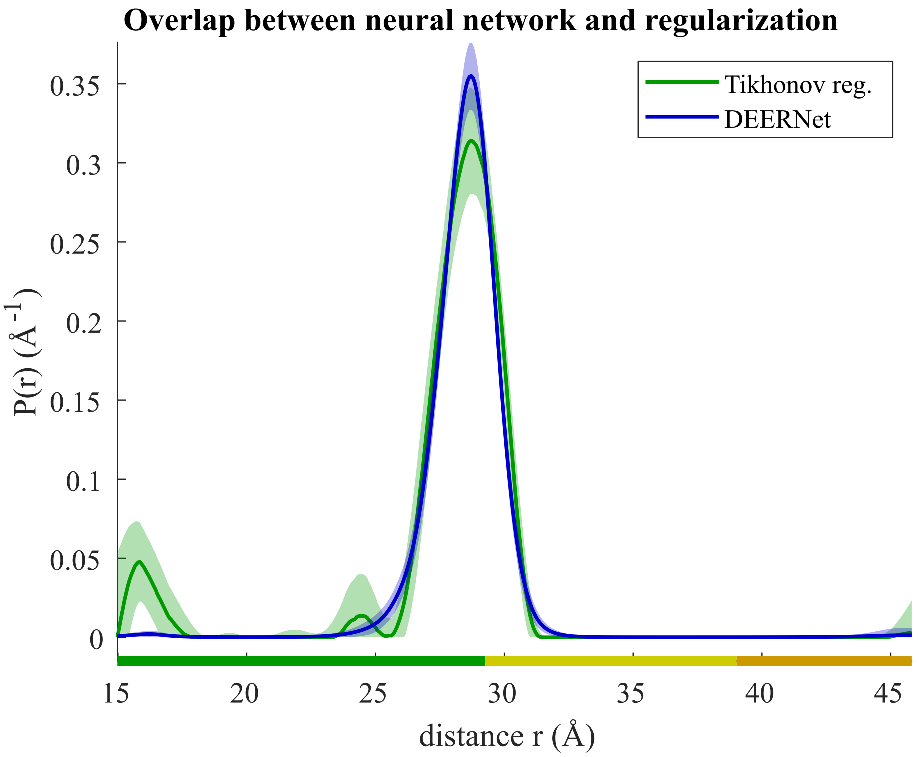


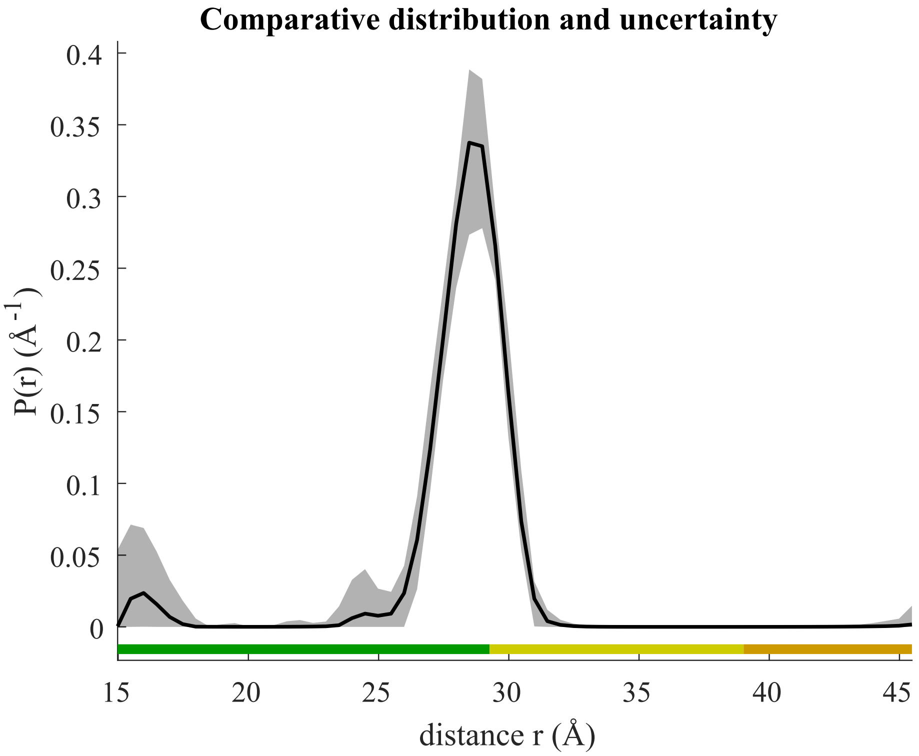

## 2. Fits of time-domain data

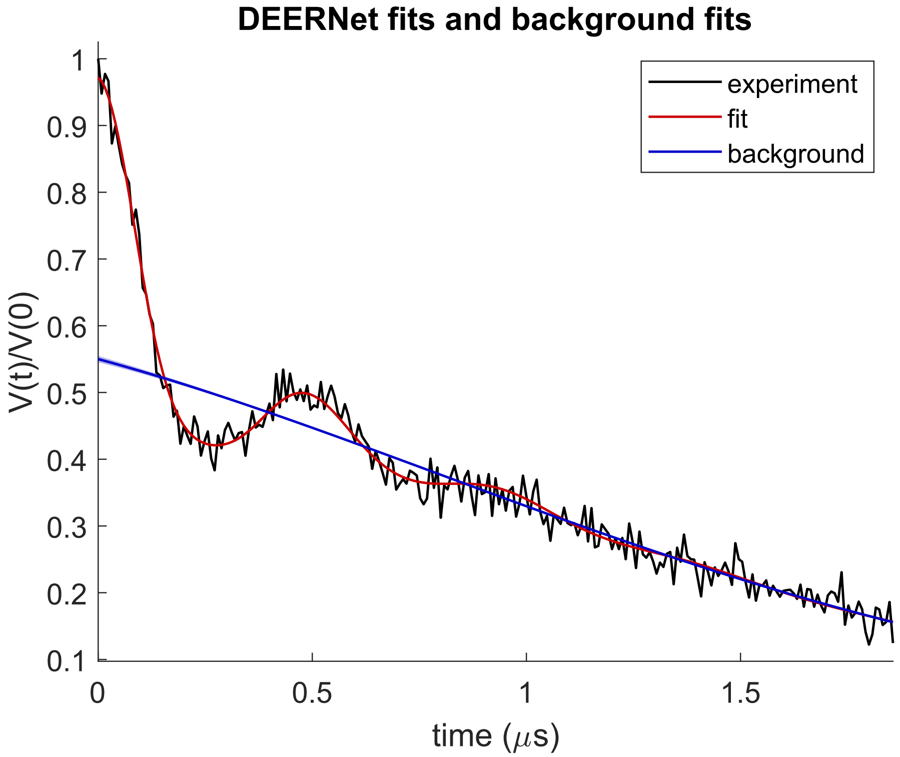


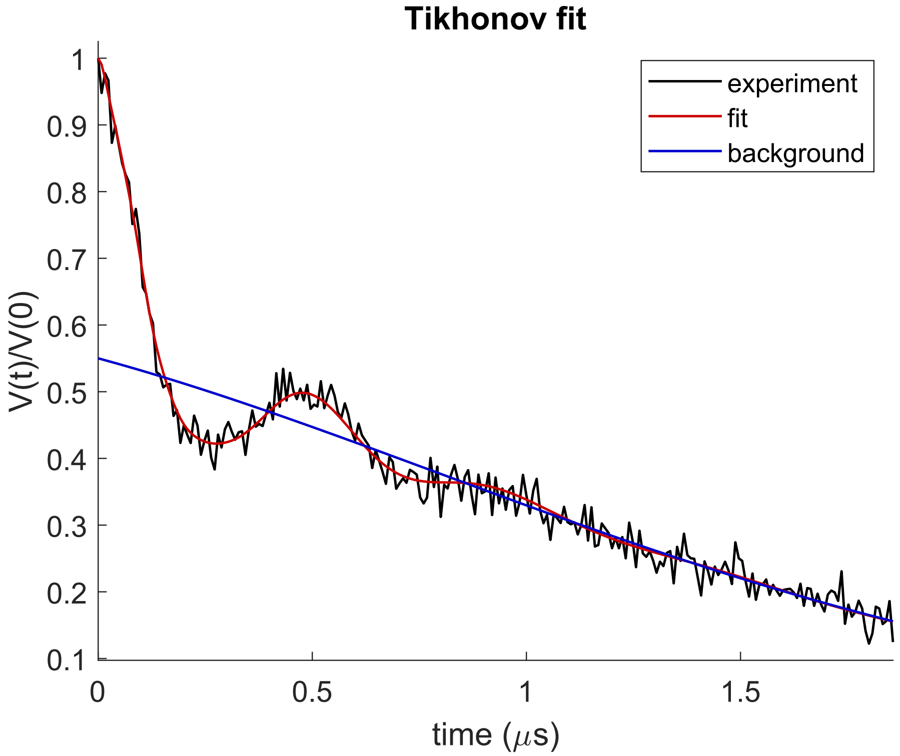

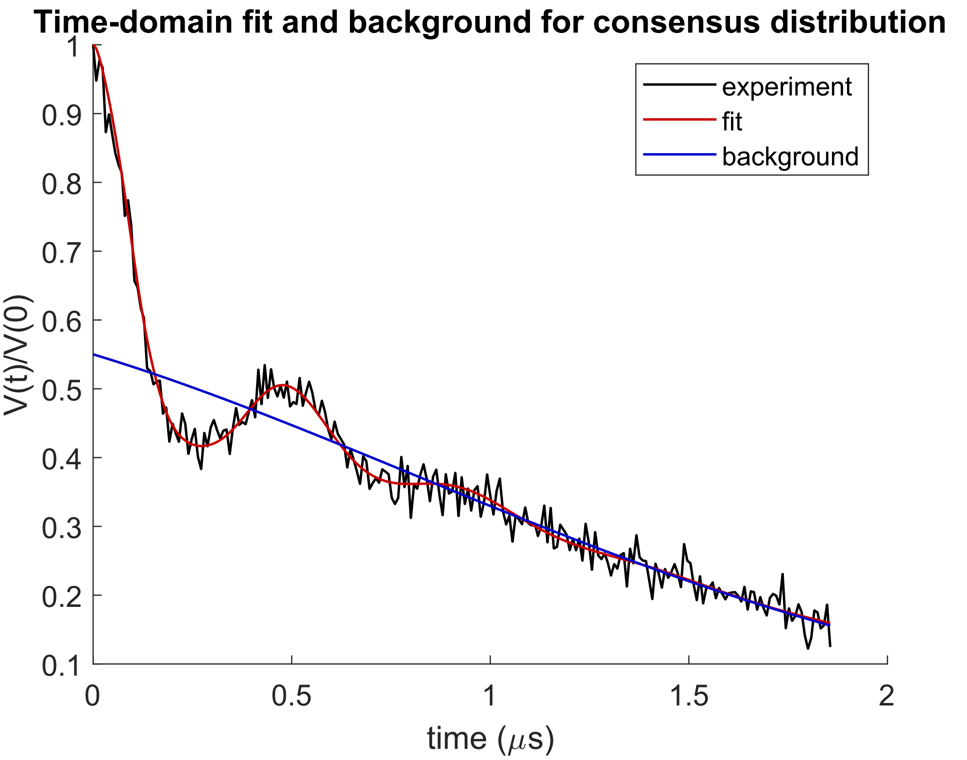
Time-domain fit and background for consensus distribution
experiment
fit
background
V(t)/V(0)
time (μs)
0
0.5
1
1.5
2
0.1
0.2
0.3
0.4
0.5
0.6
0.7
0.8
0.9
1

## 3. Experimental and processing parameters

**RIDME processing was requested. Only DEERNet output.**

Modulation depth: 0.434

Signal-to-noise ratio: 21.5 (w.r.t. modulation)

Noise estimates normalized to maximum signal

From imaginary part: 0.01969

From DEERNet fit: 0.02019

From Tikhonov fit: 0.01989

Zero time: 6 ns

Maximum time: 1856 ns

Time increment: 8 ns

Phase: -0.2 degree

Ensemble of 32 neural networks

Background separation by neural network

Background dimension: 3

Regularization parameter by best overlap with neural network solution

Regularization parameter used: 0.56

Reg. par. initial estimate by L-curve corner: 6.31

Overlap between DEERNet and regularization solutions: 0.894

Predicted overlap of consensus solution with ground truth: 0.76...0.93

Mean distance: 28.5 Å

Distance standard deviation: 1.3 Å

Full data set in Matlab format:
C:\Users\Katrin\Desktop\213_4_50nM_vt\2330417_KAq213.4_vtctRIDME_60_0_comparative_DEER_analysis.mat

Distance distributions in text format:
C:\Users\Katrin\Desktop\213_4_50nM_vt\2330417_KAq213.4_vtctRIDME_60_0_consensus_DEER_distribution.csv

Fit and background in text format:
C:\Users\Katrin\Desktop\213_4_50nM_vt\2330417_KAq213.4_vtctRIDME_60_0_consensus_DEER_fit.csv

Metadata:
C:\Users\Katrin\Desktop\213_4_50nM_vt\2330417_KAq213.4_vtctRIDME_60_0_comparative_DEER_meta_data.csv

# DEER analysis report on dataset 2330418_KAq213.6_vtctRIDME_60_0

## DEERNet Spinach SVN Rev 5662 and DeerLab 0.9.1 Tikhonov regularization

### ComparativeDEERAnalyzer version 2.0

see: S. G. Worswick et al., DOI: 10.1126/sciadv.aat5218, L. Fabregas Ibanez et al., DOI: 10.5194/mr-1-209-2020

12-Sep-2023 15:54:01

## 1. Distance distributions

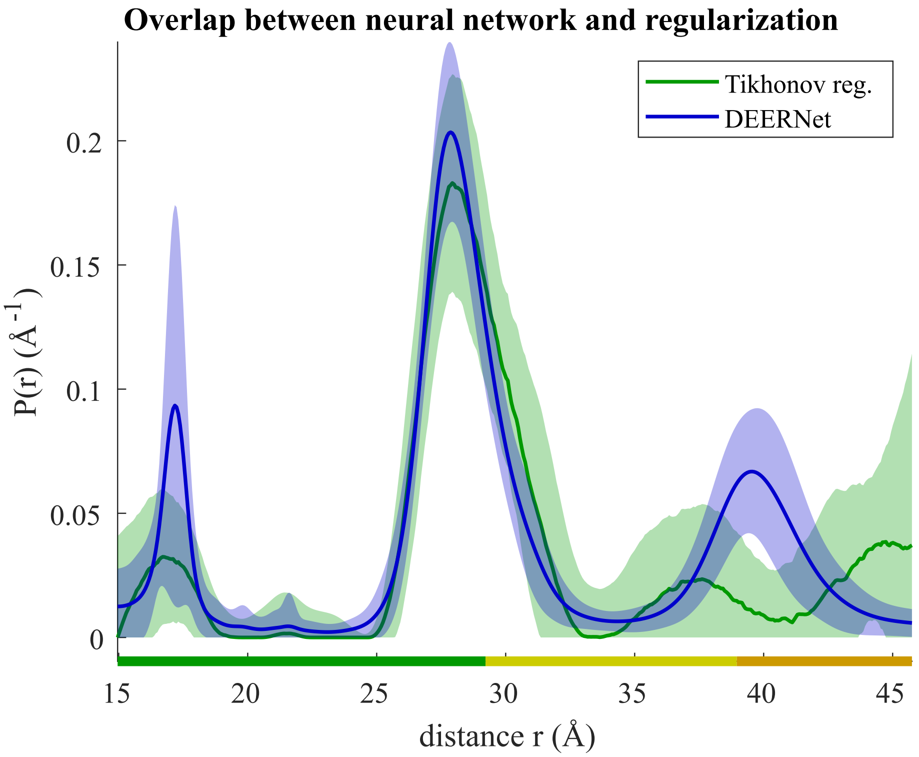


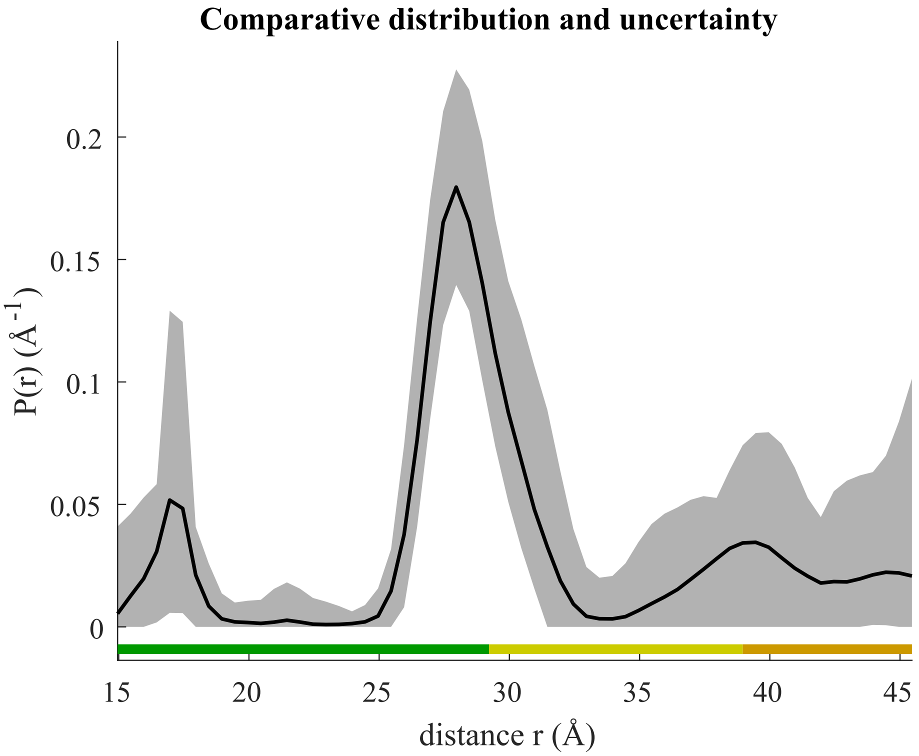

## 2. Fits of time-domain data

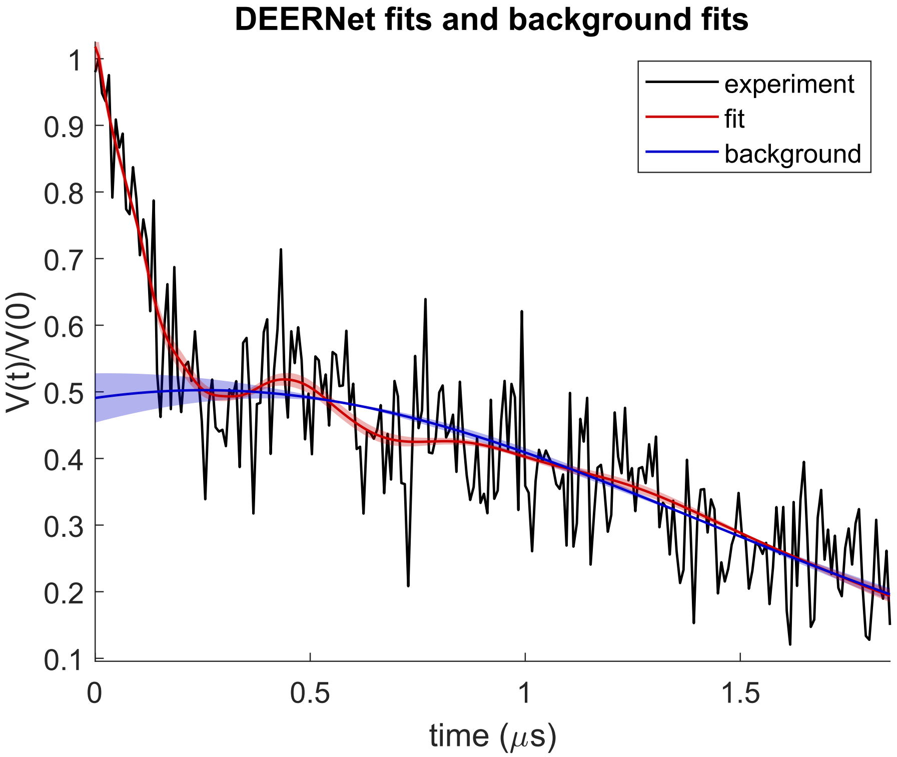


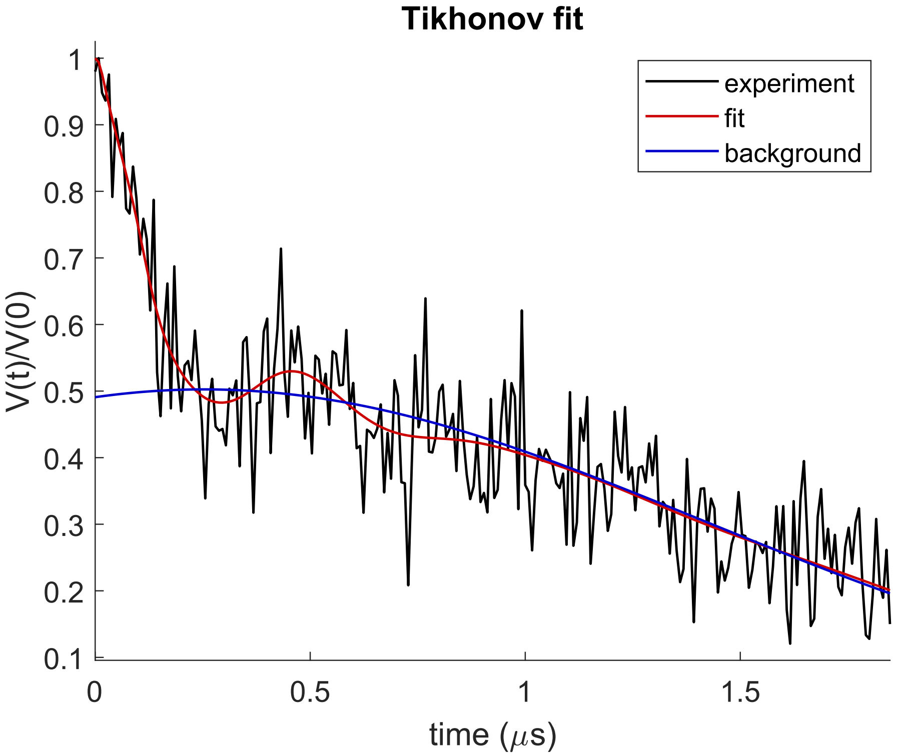

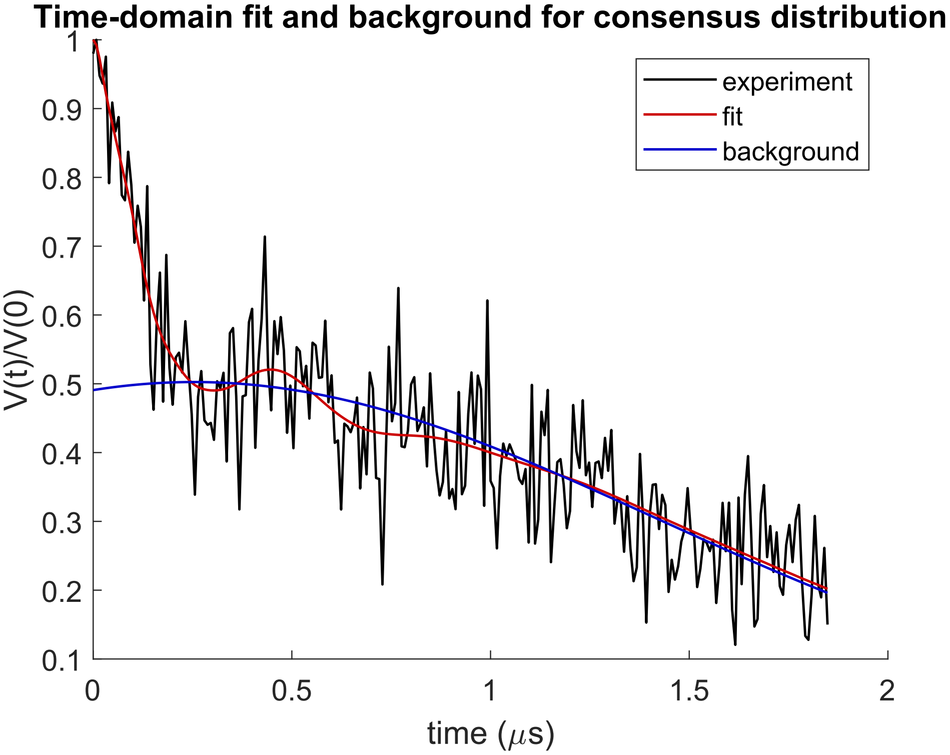
Time-domain fit and background for consensus distribution
experiment
fit
background
V(t)/V(0)
time ($\mu$s)
0
0.5
1
1.5
2
0.1
0.2
0.3
0.4
0.5
0.6
0.7
0.8
0.9
1

## 3. Experimental and processing parameters

**RIDME processing was requested. Only DEERNet output.**

**Use of distribution not recommended, since signal-to-noise ratio is below 10**

Modulation depth: 0.518

Signal-to-noise ratio: 7.3 (w.r.t. modulation)

Noise estimates normalized to maximum signal

From imaginary part: 0.07896

From DEERNet fit: 0.07123

From Tikhonov fit: 0.07059

Zero time: 15 ns

Maximum time: 1848 ns

Time increment: 8 ns

Phase: -0.6 degree

Ensemble of 32 neural networks

Background separation by neural network

Background dimension: 3

Regularization parameter by best overlap with neural network solution

Regularization parameter used: 3.54

Reg. par. initial estimate by L-curve corner: 5.01

**Overlap between DEERNet and regularization solutions: 0.781**

Predicted overlap of consensus solution with ground truth: 0.66...0.83

Mean distance: 29.6 Å

Distance standard deviation: 5.7 Å

Full data set in Matlab format:
C:\Users\Katrin\Desktop\213_6_25nM_vt\2330418_KAq213.6_vtctRIDME_60_0_comparative_DEER_analysis.mat

Distance distributions in text format:
C:\Users\Katrin\Desktop\213_6_25nM_vt\2330418_KAq213.6_vtctRIDME_60_0_consensus_DEER_distribution.csv

Fit and background in text format:
C:\Users\Katrin\Desktop\213_6_25nM_vt\2330418_KAq213.6_vtctRIDME_60_0_consensus_DEER_fit.csv

Metadata:
C:\Users\Katrin\Desktop\213_6_25nM_vt\2330418_KAq213.6_vtctRIDME_60_0_comparative_DEER_meta_data.csv
