# Supplementary material for: Pulse Dipolar Electron Paramagnetic Resonance Spectroscopy Distance Measurements at Low Nanomolar Concentrations: The CuII-Trityl Case
Source: J Phys Chem Lett. 2024 Jan 31;15(5):1455–61. doi: 10.1021/acs.jpclett.3c03311 (PMC10860127; doi:10.1021/acs.jpclett.3c03311)
Supplement: Supplementary file 2 — jz3c03311_si_002.pdf [file jz3c03311_si_002.pdf]

jz-2023-03311t.R1

Name: Peer Review Information for "Pulse Dipolar Electron Paramagnetic Resonance Spectroscopy Distance Measurements at Low Nanomolar Concentrations: the Cu^{II}-Trityl Case"

First Round of Reviewer Comments

Reviewer: 1

Comments to the Author

This manuscript reports on a sensitivity improvement in pulsed dipolar spectroscopy by using the Cu(II)dHis/trityl label combination together with the variable-time RIDME experiment. The work is on a high technical level and comparison to the CuII)dHis/nitroxide label combination as well as between constant-rime and variable-time RIDME is well done. However, I have some concerns on the reported concentration sensitivity as well as on discussion of limitation of this approach and on other approaches that require major revision. The manuscript is mostly clear and well written. Some details in presentation require minor revision.

Major:

1. Three of the four authors are also authors of a recent community paper that defined standards for measuring and analyzing PELDOR/DEER data on nitroxides (Ref. 4). I see with great dismay that the current manuscript does not live up to the standards set in the community paper. The community agreed that signal-to-noise ratio (SNR, defined as modulation-to-noise ratio) should exceed 20. The authors should not claim a concentration sensitivity that refers to data with lower SNR than that.

2. The same community paper discourages the use of the two-step data analysis whose results the authors show in the main text. Instead, it recommends the use of automated workflows. The community paper specifically requests that parameter choices in data analysis have no element of user discretion and that uncertainty bands are reported as 95% confidence intervals. The uncertainty bands reported in the main text were derived by an outdated approach and are statistically not well defined. Two different criteria were used for selecting the regularization parameter (L curve and GCV). It is unclear which data set was processed with which criterion and why. For the 5 nM sample, less noise was added than for the other samples. The authors did

analyze their data with state-of-the-art software, too, but they do not base their conclusions on the results of such analysis. This must be fixed.

3. Reporting concentration sensitivity for pulsed dipolar spectroscopy only makes sense together with reporting of the measured distance. This is because concentration sensitivity depends very strongly on distance. The Abstract and the Conclusion must state the measured distance of 3 nm together with the concentration sensitivity.

4. I object to the sentence "It is conceivable that these are often concentration-limited, either due to poor solubility or low expression yield". Conceivable it may be, but I have never heard of it or experienced it in my own research. Nitroxide-nitroxide PELDOR/DEER with a state-of-the-art high-power Q-band spectrometer is feasible with any expression yield at which a protein can still be handled in a wetlab. If the authors know a case, where more sensitive experiments, such as smFRET, were feasible and nitroxide-nitroxide PELDOR was not, they should provide a reference. The same applies to solubility. Some proteins undergo liquid-liquid phase separation already at concentrations where nitroxide-nitroxide PELDOR is not applicable. Measuring the dispersed state of such proteins is a case where the approach of the authors would be required.

5. If the authors want to argue with in-cell EPR, they should discuss whether a Cu(II)-dHis-NTA-labelled protein is stable in cell for the time required to prepare a sample. I doubt that. The authors could convince me and the readers by a reference.

6. The authors selectively discuss advantages of their approach and do not mention disadvantages. For instance, SLIM is (much) larger than a nitroxide label. Thus, SLIM is more prone to disturbing native structure. The Cu(II) labelling with the dHis motif puts more stringent requirements to the labelling site than approaches based on a single cysteine residue. Cu(II) labelling may also be problematic for proteins that bind Cu(II) unspecifically. Such limitations need to be mentioned.

Minor:

7. The authors argue that the SLIM label improves sensitivity because rectangular pulses cannot excite the whole nitroxide spectrum. What about Cu(II)-nitroxide RIDME with a chirp observer sequence?

8. Table S4, report should report the raw S values (modulation-to-noise ratios). The sensitivity per unit time can be reported as well. In this case, the unit must be given.

9. The abbreviation “ctvtRIDME” in the SI is undefined. To me it is also unclear what the “ct” means here.

Reviewer: 2

Comments to the Author

With recent development of DEER measurements, the key issues including the stability and quality of spin labels in structural biology still remain. The manuscript presents an way to improve the sensitivity of DEER measurement with trityl-Cu(II) couple following the published NO-Cu(II) pair. Overall, the experiments were well designed and the results are sound. However, the reviewer has several serious reservations that I wish the authors address correctly.

Major:

1) In the introduction, the reference 2 in line 49 is wrong. It is not the in-cell NO measurements. The authors have to cite correct references. In addition, there are too many self-citations and I suggest the authors cite the references probably in this field.

2) The authors argue the improvement of trityl-Cu(III) DEER signal to noise, which I certainly agree, but the reliability of low-concentration data has to be addressed clearly. For example, in Figure 2c (25 nm and 10 nm) the varied distance distributions in the low-concentration data compared with the high-concentration ones. The additional distance in the sample of 25 nm, which is in great contrast to the others. Similarly, in Figure 3c.

These inconsistences have to be explained and convince the users the shortened measurement time does not sacrifice the signal to noise and reliability.

3) One serious comment: the author use the two His in a protein and one additional NTA to chelate the copper ion, the stability of this trinary complex has to be discussed in very low concentration of protein samples, and preparations of the samples rather than direct dilutions from concentrated ones. Otherwise, this gain is not convincing for the EPR users.

Minor: It would be nice to comment on the in-cell applications of this method.

Reviewer: 3

Comments to the Author

Referee report

Manuscript title: “Enhanced sensitivity for pulse dipolar EPR spectroscopy using variable time RIDME”

Authors: Joshua L. Wort et al.

Submitted to JPC Letters

The manuscript describes approaches to improved sensitivity of distance determination by RIDME, a pulsed EPR technique widely used. A combination of using a trityl spin label instead of the commonly used nitroxides and the application of a procedure to optimize RIDME parameters lead to an improvement in sensitivity that promises reaching the 10 nanomolar range of protein concentrations.

The study clearly is a large step ahead in the sensitivity of distance determination by pulsed dipolar spectroscopy and merits speedy publication in the form of a letter.

Several points need the attention of the authors:

1. In Fig. 3, the data are shown for the 50 nanomolar and 25 nanomolar concentrations, and in contrast to Fig. 2, conventional RIDME, the accumulation times are the same. The concentration changes by a factor of two, yet the noise in the raw data (Fig. 3a) seems disproportionally higher for 25 nanomolar, compared to 50 nanomolar. Also, the uncertainty range (Fig. 3c) for the former sample is significantly worse. Perhaps the latter point is owing to the overly conservative error estimate. Perhaps the authors could comment on this point in the SI.

2. Overall the manuscript is well written and clear, Yet, on p. 8, lines 9 and 10, “lower concentrations led to significantly increased confidence bands“ can lead to confusion: increased confidence, means less confidence in this case, so rephrasing would be good.

3. On p. 7, line 34,” Thus, compared to the CuNTA-nitroxide RIDME measured at 30 K, 52 here the unfavourable change in the Boltzmann distribution res…“ This description is clear to magnetic resonance audiences, yet for the readers of JPC letters this seems too specialized. The authors could consider rephrasing or referring to the SI for details.

4. As rightly pointed out, the protein is a well-chosen model system with a stable fold and a relative short distance between the label positions. Consequently, relatively short evolution times and clear modulation facilitate the experiment. Could the authors sketch how, in their view, less optimal situations would affect the concentration limit?

Once these points are taken care of, this is a fine manuscript that is relevant for protein molecular structure investigations and will be of interest for a large range of the readership of JCPLett. The urgency of this publication derives from the fact that the nanomolar sensitivity approached here

represents one of the bottlenecks for using the pulsed dipolar EPR spectroscopy methods to real, biochemically relevant questions.

iew Comments:

Dr Bela Bode
Biomolecular Sciences Research Complex
Centre of Magnetic Resonance

Manuscript: "Pulse Dipolar Electron Paramagnetic Resonance Spectroscopy Distance Measurements at Low Nanomolar Concentrations: the Cu^{II}-Trityl Case"

St Andrews, 24th December 2023

Dear Editors,

We are submitting the revised manuscript "***Pulse Dipolar Electron Paramagnetic Resonance Spectroscopy Distance Measurements at Low Nanomolar Concentrations: the Cu^{II}-Trityl Case***" authored by Dr Katrin Ackermann, Mr Caspar Heubach, Prof. Olav Schiemann, and Dr Bela E. Bode for your kind consideration for publication as a Letter in *The Journal of Physical Chemistry Letters*.

We would like to thank the reviewers for their efforts in carefully assessing the manuscript. The reviewer and editorial comments and our point-by-point reply can be found overleaf. We believe the reviewers' suggestions have improved manuscript and supplementary information. Therefore, we hope the current manuscript is acceptable for publication in *The Journal of Physical Chemistry Letters*.

The manuscript has been published as an author preprint on chemRxiv (DOI:10.26434/chemrxiv-2023-j6mvr). To meet institutional and research funder open access requirements, any accepted manuscript arising shall be open access under a Creative Commons Attribution (CC BY) reuse licence with zero embargo.

Sincerely yours,

[redacted]

Purdie Building, North Haugh, St Andrews, Fife, Scotland, United Kingdom, KY16 9ST
T: +44 (0)1334 463869 F: +44 (0)1334 463808
E: beb2@st-andrews.ac.uk

The University of St Andrews is a charity registered in Scotland, No: SC013532

Responses to reviewer comments

We thank all three reviewers for carefully reading the manuscript and providing detailed suggestions for improvement. In the following we provide the comments in black text, with point-by-point responses in blue text. We have carefully addressed all comments and believe the readability and clarity of manuscript and supplementary information have been improved.

Therefore, we hope the current manuscript is acceptable for publication in *The Journal of Physical Chemistry Letters.*

Reviewer: 1

1. Three of the four authors are also authors of a recent community paper that defined standards for measuring and analyzing PELDOR/DEER data on nitroxides (Ref. 4). I see with great dismay that the current manuscript does not live up to the standards set in the community paper. The community agreed that signal-to-noise ratio (SNR, defined as modulation-to-noise ratio) should exceed 20. The authors should not claim a concentration sensitivity that refers to data with lower SNR than that. While this may sound pedantic, all four authors of the current manuscript are also authors of reference 4. Reference 4 describes guidelines for authors and reviewers of scientific outputs involving PELDOR/DEER data. While we agree that some considerations are equally valid for RIDME experiments, we must refer the referee to footnote b of table 3 in reference 4 that explicitly states: “If an SNR of 20 cannot be achieved at the required trace length, data with an SNR down to 10 can still provide useful restraints.” We have added the following statement to make this clear to the reader: “While this is below the recent recommendation of a modulation-to-noise ratio of at least 20, this data is still of sufficient quality to provide useful structural restraints.[4]”

2. The same community paper discourages the use of the two-step data analysis whose results the authors show in the main text. Instead, it recommends the use of automated workflows. The community paper specifically requests that parameter choices in data analysis have no element of user discretion and that uncertainty bands are reported as 95% confidence intervals. The uncertainty bands reported in the main text were derived by an outdated approach and are statistically not well defined. Two different criteria were used for selecting the regularization parameter (L curve and GCV). It is unclear which data set was processed with which criterion and why. For the 5 nM sample, less noise was added than for the other samples. The authors did analyze their data with state-of-the-art software, too, but they do not base their conclusions on the results of such analysis. This must be fixed. The community white paper does not state that the two-step approach is wrong and must not be used. Instead, it points to the issue and calls for reliable and tested single step approaches. The paper does also not call for all legacy data to be re-analysed. We choose here to use both, the two- and single-step approaches. This is exactly the approach taken in reference 4, where all laboratories participating in the ring-test did use the two-step analysis and the comparison to newer approaches was done in the supplement. In addition, it is also not so clear which of the analyses is supposedly incorrect, and the reviewer does not specify this. Along these lines, the

Consensus DEER Analyzer software fails to process certain data despite the modulation-to-noise ratio being above 10 and no other obvious problem with the data. We are confident that single-step and automated approaches will be widely adopted once their general applicability and robustness has been established and their documentation and usability meet user demand.

We have clarified the choice of the regularisation parameter in the SI (chapter 8, page S9). Furthermore, the data will become openly accessible, and anyone concerned can retrace the analysis and draw their own conclusions.
Adding lower amounts of noise to the 5 nM sample was done to demonstrate that the failed distance analysis is not caused by the noise added in validation. Please note that we specifically point out that in this case, even though only 10% noise was added, we do not deem the resulting distance distribution reliable.

3. Reporting concentration sensitivity for pulsed dipolar spectroscopy only makes sense together with reporting of the measured distance. This is because concentration sensitivity depends very strongly on distance. The Abstract and the Conclusion must state the measured distance of 3 nm together with the concentration sensitivity.
We have added the requested clarification to abstract and conclusion.

4. I object to the sentence "It is conceivable that these are often concentration-limited, either due to poor solubility or low expression yield". Conceivable it may be, but I have never heard of it or experienced it in my own research. Nitroxide-nitroxide PELDOR/DEER with a state-of-the-art highpower Q-band spectrometer is feasible with any expression yield at which a protein can still be handled in a wetlab. If the authors know a case, where more sensitive experiments, such as smFRET, were feasible and nitroxide-nitroxide PELDOR was not, they should provide a reference. The same applies to solubility. Some proteins undergo liquid-liquid phase separation already at concentrations where nitroxide-nitroxide PELDOR is not applicable. Measuring the dispersed state of such proteins is a case where the approach of the authors would be required.
We rephrased this sentence by removing "often". We also added a statement and referenced liquidliquid phase separation. Providing references where smFRET worked but PELDOR did not is difficult to do because failed experiments are rarely reported in the literature. However, examples from the lab of OS are sea urchin and human SAC I and TRIM71 (Kolanus, collaborator of OS). We would love to be able to express these in large enough amounts for PELDOR studies but are currently only able to reach sample amounts that would satisfy smFRET.

5. If the authors want to argue with in-cell EPR, they should discuss whether a Cu(II)-dHis-NTA-labelled protein is stable in cell for the time required to prepare a sample. I doubt that. The authors could convince me and the readers by a reference.
We have added the relevant paper from the Ruthstein lab:
"Despite its vulnerability to reduction CuNTA has been used for in-cell PDS.[52]"

6. The authors selectively discuss advantages of their approach and do not mention disadvantages. For instance, SLIM is (much) larger than a nitroxide label. Thus, SLIM is more prone to disturbing native structure. The Cu(II) labelling with the dHis motif puts more stringent requirements to the labelling site

than approaches based on a single cysteine residue. Cu(II) labelling may also be problematic for proteins that bind Cu(II) unspecifically. Such limitations need to be mentioned.
We write now: “Another approach uses Cu^{II} complexed with nitrilotriacetic acid (CuNTA) coordinated to a site-specifically introduced double-histidine (dHis) motif (dHis-CuNTA), posing more stringent requirements on the labelling site but yielding very narrow distance distributions due to the rigidity of this labelled side-chain.[35,42,47-50]”

We also added a sentence to the introduction: “Additionally, different spin labels may lead to different degrees of perturbation of native structure due to their size and interactions with protein residues, e.g. trityl labels.”

We also have added a sentence clarifying that CuNTA labelling is not prone to unspecific binding as labelling with Cu without chelator is: “CuNTA increases specificity to dHis sites compared to free Cu^{II} in solution, which has higher propensity to unspecific binding.[42,47,51]”

Minor:

7. The authors argue that the SLIM label improves sensitivity because rectangular pulses cannot excite the whole nitroxide spectrum. What about Cu(II)-nitroxide RIDME with a chirp observer sequence?
We did not try this, but literature suggests no improvement https://doi.org/10.1016/j.jmr.2019.07.047.

8. Table S4, report should report the raw S values (modulation-to-noise ratios). The sensitivity per unit time can be reported as well. In this case, the unit must be given. We added the raw S (modulation-to-noise ratio) values.

9. The abbreviation “ctvtRIDME” in the SI is undefined. To me it is also unclear what the “ct” means here.
We have added the definition to the SI: ctvtRIDME (constant and variable time RIDME in a single combined pulse program).

Reviewer: 2

With recent development of DEER measurements, the key issues including the stability and quality of spin labels in structural biology still remain. The manuscript presents an way to improve the sensitivity of DEER measurement with trityl-Cu(II) couple following the published NO-Cu(II) pair. Overall, the experiments were well designed and the results are sound. However, the reviewer has several serious reservations that I wish the authors address correctly.

We thank the reviewer for considering our experiments "well designed" and the results "sound". However, we would like to emphasise that the manuscript deals with RIDME and not DEER experiments. This confusion of PDS methods raises some concern regarding the suitability of this reviewer.

1) In the introduction, the reference 2 in line 49 is wrong. It is not the in-cell NO measurements. The authors have to cite correct references. In addition, there are too many self-citations and I suggest the authors cite the references probably in this field.

The paragraph in question discusses Pulsed Dipolar EPR Spectroscopy (and not just PELDOR), the need for paramagnetic centres, and their use in *in vitro* and *in cellulo* experiments. This is not limited to nitroxides. Reference 2 from the Bordignon lab describes *in vitro* measurements on nitroxide labels and *in cellulo* measurements on Gd^{III} labels. All of this is within scope of the text, and we cannot follow where this is supposed to be erroneous.

The reviewer seems to be misled that this is a PELDOR/DEER manuscript. We report and discuss RIDME applications and the use of CuNTA and trityl. Given that our labs have made significant contributions to the development and applications of RIDME (including the vtRIDME experiment), CuNTA and SLIM labelling (including the development of SLIM) the references reflect this. The other reviewers are not concerned by our reference list.

2) The authors argue the improvement of trityl-Cu(III) DEER signal to noise, which I certainly agree, but the reliability of low-concentration data has to be addressed clearly. For example, in Figure 2c (25 nm and 10 nm) the varied distance distributions in the low-concentration data compared with the highconcentration ones. The additional distance in the sample of 25 nm, which is in great contrast to the others. Similarly, in Figure 3c.

These inconsistences have to be explained and convince the users the shortened measurement time does not sacrifice the signal to noise and reliability.

We cannot follow the reviewer's concern. There are no additional distances outside the confidence estimates. While there is distance probability appearing, it is within uncertainty. Thus, the analysis shows exactly what is expected. Larger noise gives larger uncertainty, but no new distance artefacts as suggested by reviewer 2.

3) One serious comment: the author use the two His in a protein and one additional NTA to chelate the copper ion, the stability of this trinary complex has to be discussed in very low concentration of protein samples, and preparations of the samples rather than direct dilutions from concentrated ones. Otherwise, this gain is not convincing for the EPR users.

All samples were prepared from mixing diluted protein stocks with diluted CuNTA stocks not diluting the final complex as suggested by the reviewer (this is described in detail in the reference given – ref. 55 in the main text, ref. 1 in the SI). Thus, complex stability is not a problem. As common for multidentate ligands,

CuNTA is orders of magnitude more stable than the complex formed with the dHis motif. Dissociation constants assumed for labelling are cited and the CuNTA excess is calculated to lead to 90% labelling.

Minor: It would be nice to comment on the in-cell applications of this method.
There have not yet been any in cell applications of this method. But we cite a paper from the Ruthstein lab on the use of dHisCuNTA in *in cellulo* experiments (see response to reviewer 1).

Reviewer: 3

Manuscript title: "Enhanced sensitivity for pulse dipolar EPR spectroscopy using variable time RIDME"
Although this is not the manuscript title the comments clearly refer to the manuscript in question.

Authors: Joshua L. Wort et al.
Dr Wort is not an author on the current manuscript.

1. In Fig. 3, the data are shown for the 50 nanomolar and 25 nanomolar concentrations, and in contrast to Fig. 2, conventional RIDME, the accumulation times are the same. The concentration changes by a factor of two, yet the noise in the raw data (Fig. 3a) seems disproportionally higher for 25 nanomolar, compared to 50 nanomolar. Also, the uncertainty range (Fig. 3c) for the former sample is significantly worse. Perhaps the latter point is owing to the overly conservative error estimate. Perhaps the authors could comment on this point in the SI.

We have added a sentence to the SI to discuss this:
"The 25 nM sample is worse than extrapolation would suggest. As we cannot measure the final concentrations but just the stocks and then dilute this may be an error or just variation. However, we believe it would be unethical to remove a data point for no objective reason."

2. Overall the manuscript is well written and clear, Yet, on p. 8, lines 9 and 10, "lower concentrations led to significantly increased confidence bands" can lead to confusion: increased confidence, means less confidence in this case, so rephrasing would be good.

We rephrased to: "lower concentrations led to significantly increased uncertainties including broader confidence bands and such data should only be interpreted with great care."

3. On p. 7, line 34," Thus, compared to the CuNTA-nitroxide RIDME measured at 30 K, 52 here the unfavourable change in the Boltzmann distribution res…" This description is clear to magnetic resonance audiences, yet for the readers of JPC letters this seems too specialized. The authors could consider rephrasing or referring to the SI for details.

We rephrased to: "… here the unfavourable change in thermal polarisation resulting from a 10 K increase in temperature was outweighed by faster repetition rate with higher temperature."

4. As rightly pointed out, the protein is a well-chosen model system with a stable fold and a relative short distance between the label positions. Consequently, relatively short evolution times and clear modulation facilitate the experiment. Could the authors sketch how, in their view, less optimal situations would affect the concentration limit?

We have added to the conclusion paragraph:
"It is expected that longer distances and broader or more complex distributions will require longer dipolar evolution times or better modulation-to-noise ratio. This would likely demand higher concentrations than achieved here."

Editorial comments:

1. Please submit your publication files without any markups. Any copies that contain highlights, colored text, or tracked changes should be submitted as "Supporting Information for Review Only. We will submit manuscript and SI files without markup and add files with changes highlighted in yellow as review only material.

2. Title: Using acronyms in title is discouraged. Please spell out all acronyms in the title of the manuscript and Supporting Information.
We have changed the title to "Pulse Dipolar Electron Paramagnetic Resonance Spectroscopy Distance Measurements at Low Nanomolar Concentrations: the Cu^{II}-Trityl Case".

3. References: In both the main file and the supporting information, fix the style of all references to use JPCL formatting (check all references carefully). ***JPC Letters reference formatting requires that journal references should contain: () around numbers, author names, article title (titles entirely in title case or entirely in lower case), abbreviated journal title (italicized), year (bolded), volume (italicized), and pages (first-last). Book references should contain author names, book title (in the same pattern), publisher, city, and year. Websites must include date of access.
We have checked all references to match the current JPC Letters style.

4. Graphics: One or more of your figure legends includes a citation. Permission is required if you are using another publisher's or copyright owner's figures/tables verbatim, adapting or modifying them, or using them in part. Permission may not be required if you are only using data to create your figure. If this is the case, please notify our office. Additionally, permission is not required when images are reused from ACS publications.

In all cases of reused or adapted images, even those from papers published open access or in ACS publications, you must cite the source with a credit line in the figure legend. Please use the following format: "Reproduced/adapted from [REFERENCE #]. Copyright [YEAR] [Publisher/Copyright holder]."

You must upload all required permissions documents as individual PDF files with the designation "Other files for Editors only". Please ensure each document is named for the corresponding graphic (e.g. "Permissions-Figure 1"). If the document list is extensive, you may consider compressing the files and uploading a ".zip" file as "Other files for Editors only". If permissions are not accessible, then the graphics need to be redrawn, replaced, or removed. For details, see: […]
No graphics have been reused, we add citations to refer to the science and methods used. No permissions are required.

5. Supporting Information: Please number SI pages in the following format: "S1, S2…" Page numbering has been changed as requested.

jz-2023-03311t.R2

Name: Peer Review Information for "Pulse Dipolar Electron Paramagnetic Resonance Spectroscopy Distance Measurements at Low Nanomolar Concentrations: the Cu^{II}-Trityl Case"

Second Round of Reviewer Comments

Reviewer: 2

Comments to the Author

The revised version addressed partially the pointes raised by the reviewer. The reviewer admits fact of the mis-typed DEER and RIDME in previously submitted comments, but understands the meaning of DEER, RIDME and DQC. All the distance measurement relies on key: the memory time of the spin label during the dipolar evolution time, which restricts the distance limits for determination. The reviewer would not argue the theory of this work since there is no development in this direction in this work. However, the review would ask the author to address carefully the concentration of samples for distance measurements since reliable distance is essential in these experiments.

1) Reference 2 is an excellent work on the stability assay of NO spin labels for in cell measurements. It is the stability of NO spin label itself for in cell distance measurement but not the concentration of protein samples. This has to be stated clearly in the manuscript and it has to be for the EPR users.

2) Again, the signal to noise in the measured data for low concentrations. The reviewer pointed out the inconsistency is the reliability of the data for very low concentration.

3) The reviewer understands the high stability of NTA-Cu. However, the binding affinity of NTA-Cu for two additional HIS is certainly variable and it is also geometry and buffer (potentially binding ligands or pH) dependent. The reviewer's concern is the author has to state the case objectively, because the author used only one case of GB1 K28H/Q32H for such small and rock protein. If they would claim their proposed measurement more convincing, they have to measure a number of two His mutants of different proteins. One case is just not convincing.

I wish the authors to state the comments clearly.

The points of 2 and 3 were already pointed by reviewer 1 in different ways.

Reviewer: 3

Comments to the Author

Referee report

Revised manuscript title: “Pulse Dipolar Electron Paramagnetic Resonance Spectroscopy Distance Measurements at Low Nanomolar Concentrations: the CuII -Trityl Case”

Authors: Ackermann et al.

Submitted to JPC Letters

My points are largely answered by the revision.

I am a bit confused about the answer to my question 1.:

The measurements of the samples with concentrations of 50 and 25 nanomolar in Fig. 3 are the only ones where the data are presented in such a way that a direct comparison of S/N can be made. For the other concentrations, measurement conditions differ, making such a comparison impossible. The authors argue that the 25 nanomolar sample is ‘an outlier’, yet fail to show that for the remaining concentrations the S/N indeed follows the ‘extrapolated trend’ of S/N vs. concentrations. Otherwise, the suggestion that the 25 nanomolar sample is an outlier, together with the rather vague statement that the concentrations themselves may be flawed, because based on dilutions only, is weak. Perhaps the authors should give confidence levels for the concentrations, particularly for the 5 nanomolar concentration.

Author's Response to Peer Review Comments:

Dr Bela Bode
Biomolecular Sciences Research Complex
Centre of Magnetic Resonance

Manuscript: "Pulse Dipolar Electron Paramagnetic Resonance Spectroscopy Distance Measurements at Low Nanomolar Concentrations: the Cu^{II}-Trityl Case"

St Andrews, 10th January 2024

Dear Editors,

We are submitting the revised manuscript "***Pulse Dipolar Electron Paramagnetic Resonance Spectroscopy Distance Measurements at Low Nanomolar Concentrations: the Cu^{II}-Trityl Case***" authored by Dr Katrin Ackermann, Mr Caspar Heubach, Prof. Olav Schiemann, and Dr Bela E. Bode for your kind consideration for publication as a Letter in *The Journal of Physical Chemistry Letters*.

We would like to thank the reviewers for their efforts in carefully assessing the manuscript. The reviewer comments and our point-by-point reply can be found overleaf. We believe we have fully addressed all remaining and newly raised concerns. Therefore, we hope the current manuscript is acceptable for publication in *The Journal of Physical Chemistry Letters*.

The manuscript has been published as an author preprint on chemRxiv (DOI:10.26434/chemrxiv-2023-j6mvr). To meet institutional and research funder open access requirements, any accepted manuscript arising shall be open access under a Creative Commons Attribution (CC BY) reuse licence with zero embargo.

Sincerely yours,

[redacted]

Purdie Building, North Haugh, St Andrews, Fife, Scotland, United Kingdom, KY16 9ST
T: +44 (0)1334 463869 F: +44 (0)1334 463808
E: beb2@st-andrews.ac.uk

The University of St Andrews is a charity registered in Scotland, No: SC013532

Responses to reviewer comments

We thank all reviewers 2 and 3 for carefully reading the manuscript and providing additional detailed suggestions for improvement. In the following we provide the reviewers' comments in black text, with point-by-point responses in blue text. We have carefully addressed all their comments, and believe the clarity of manuscript and supplementary information have been further improved.

Therefore, we hope the current manuscript is acceptable for publication in *The Journal of Physical Chemistry Letters.*

Reviewer: 2

Recommendation: This paper may be publishable, but major revision is needed; I would like to be invited to review any future revision.

Comments:
The revised version addressed partially the pointes raised by the reviewer. The reviewer admits fact of the mis-typed DEER and RIDME in previously submitted comments, but understands the meaning of DEER, RIDME and DQC. All the distance measurement relies on key: the memory time of the spin label during the dipolar evolution time, which restricts the distance limits for determination. The reviewer would not argue the theory of this work since there is no development in this direction in this work.
It is unclear what the reviewer is trying to express by mentioning the phase memory time of the spin label - yes, it does affect the maximum achievable dipolar evolution time, which determines the distance limits. As the reviewer will be aware the phase memory time will become concentration independent at low concentrations as e.g., instantaneous diffusion contributions will be negligible. We have refrained from discussing concentration and deuteration effects as they are well-reported. However, to make practitioners new to the field aware we have now cited two manuscripts detailing the effects of local concentration and deuteration on the phase memory time and achievable distances: "At sufficiently low temperature and spin concentration the electron spin dephasing time becomes concentration independent,[56] and the achievable distance range is largely limited by deuteration levels of the sample.[4, 57]"

However, the review would ask the author to address carefully the concentration of samples for distance measurements since reliable distance is essential in these experiments.
We understand that the reviewer considers our previous wording too nuanced and encourages us to be very explicit regarding concentration of samples limiting sensitivity, which in turn limits reliability of distance measurements. We have now stated more clearly in the manuscript text that even though the used label combination affords measurements at lower concentrations than previously reported, not all data shown is desirable for interpretation of distance distributions but used to show the trend in sensitivity and allow analysis: "Thus, concentrations below 50 nM remain a challenge for ctRIDME even when using CuNTA-SLIM label pairs, and higher-quality data with lower uncertainties than seen for 10 and 25 nM will be required to e.g., determine whether multiple distance populations are present, as observed previously."

1) Reference 2 is an excellent work on the stability assay of NO spin labels for in cell measurements. It is the stability of NO spin label itself for in cell distance measurement but not the concentration of protein samples. This has to be stated clearly in the manuscript and it has to be for the EPR users.

We have clarified that the paper by Kucher et al. describes similar (~100 nM) protein concentrations for PELDOR measurements *in vitro* using nitroxide spin labels and in-cell using gadolinium labels.

2) Again, the signal to noise in the measured data for low concentrations. The reviewer pointed out the inconsistency is the reliability of the data for very low concentration.

As stated above, we now understand that the reviewer feels we were not clear enough that Figures 2 and 3 do contain data that, while displaying the trends in improved sensitivity we aim to demonstrate, should not actually be used for obtaining high-quality structural restraints.

We have emphasised and clarified this in the revised manuscript: "we would refrain from using data obtained at lower [than 50 nM] concentrations using ctRIDME due to the poor modulation-to-noise ratios achieved" and "While for the 50 nM vtRIDME a modulation-to-noise ratio > 20 was achieved with 8 h averaging time, the 25 nM and 10 nM samples yielded ratios < 10 with 8 h and 18 h averaging time, respectively; data of this quality should not be used to interpret distance distributions."

We are further clarifying that the 25 nM sample is an outlier, see also reviewer #3.

3) The reviewer understands the high stability of NTA-Cu. However, the binding affinity of NTA-Cu for two additional HIS is certainly variable and it is also geometry and buffer (potentially binding ligands or pH) dependent. The reviewer's concern is the author has to state the case objectively, because the author used only one case of GB1 K28H/Q32H for such small and rock protein. If they would claim their proposed measurement more convincing, they have to measure a number of two His mutants of different proteins. One case is just not convincing.

I wish the authors to state the comments clearly.

We have extended the relevant part of the introduction as follows:

"Depending on the secondary structural elements (α-helix or β-sheet), dissociation constants (K_d) were determined to be in the order of 10^{-5} to 10^{-7} under EPR conditions.[49, 52] An advantage of using spectroscopically orthogonal labels is that binding sites can be saturated by an excess of CuNTA without overlap with the detected signal.[52] Furthermore, the dHis-CuNTA labelling has been shown to be robust against competing ligands and retaining its high affinity binding over a wide pH range, thus demonstrating biologically relevant compatibility.[50] While the majority of the benchmarking studies on dHis-CuNTA have been performed on different constructs of a model protein (GB1, vide infra), this labelling approach has also been applied to a variety of more complex biological systems.[30, 45, 53]"

Thus, all points of concern of the reviewer – applicability to different proteins and robustness to geometry, pH, competitors - have been investigated and we have now explicitly pointed to the relevant references.

The points of 2 and 3 were already pointed by reviewer 1 in different ways.

Reviewer 1 seemed satisfied with our revised manuscript. We believe we have fully addressed all concerns about data quality and general applicability of our findings.

Reviewer: 3

Recommendation: This paper is publishable subject to minor revisions noted. Further review is not needed.
We thank the reviewer for their positive evaluation of our revised manuscript.

Comments:
Referee report
Revised manuscript title: “Pulse Dipolar Electron Paramagnetic Resonance Spectroscopy Distance Measurements at Low Nanomolar Concentrations: the CuII -Trityl Case”
Authors: Ackermann et al.
Submitted to JPC Letters
My points are largely answered by the revision.
I am a bit confused about the answer to my question 1.:
The measurements of the samples with concentrations of 50 and 25 nanomolar in Fig. 3 are the only ones where the data are presented in such a way that a direct comparison of S/N can be made. For the other concentrations, measurement conditions differ, making such a comparison impossible. The authors argue that the 25 nanomolar sample is ‘an outlier’, yet fail to show that for the remaining concentrations the S/N indeed follows the ‘extrapolated trend’ of S/N vs. concentrations. Otherwise, the suggestion that the 25 nanomolar sample is an outlier, together with the rather vague statement that the concentrations themselves may be flawed, because based on dilutions only, is weak. Perhaps the authors should give confidence levels for the concentrations, particularly for the 5 nanomolar concentration.
We respectfully disagree with the reviewer that only the vtRIDME measurements shown in Figure 3 with concentrations of 50 and 25 nanomolar can be compared regarding their sensitivity. While the averaging times for the different measurement series and samples differ (and we do not consider it a good use of measurement time to average a high-concentration sample for days when it can be done in a few of hours, just to have the same number of scans as the low-concentration sample of a series), sensitivity considerations can be done to compare across all samples. This is described in detail in the supplementary information and has also been reported previously (for example, Wort et al, *Angew. Chem. Int. Ed.* **2019**, *58*, 11681-11685). Briefly, sensitivities are calculated by taking into account the total number of echoes per point (which means, the averaging time is included), as well as the averaging rate, making it even possible to compare sensitivities per unit time (S_t) across spin labels and experiments.
We have now included a direct reference to supplementary table S4, which shows a column for S_t extrapolated to a common concentration of 1 μM for all samples. “The sensitivity comparison also identifies the 25 nM sample as an outlier of the series, with an extrapolated sensitivity of around half of the expected value, while the 100, 50, and 10 nM samples are in good agreement with extrapolated values (see Table S4, column “S_t at 1 μM extrapolated.”
Please note that for the 5 nM sample the noise is so large that determination of the modulation depth and rmsd bear larger errors, which is why we do not consider this data of sufficient quality for processing.

jz-2023-03311t.R2

Name: Peer Review Information for "Pulse Dipolar Electron Paramagnetic Resonance Spectroscopy Distance Measurements at Low Nanomolar Concentrations: the Cu^{II}-Trityl

Case"

Final Reviewer Comments

Reviewer: 2

Comments to the Author

The revision was greatly improved and it is acceptable for publication as is.

Reviewer: 3

Comments to the Author
Referee report
Revision 2
Manuscript title: “Pulse Dipolar Electron Paramagnetic Resonance Spectroscopy Distance Measurements at Low Nanomolar Concentrations: the CuII -Trityl Case”
Authors: Ackermann et al.
Submitted to JPC Letters
I thank the authors for their answer, and indeed, I did not grasp the full significance of table S4, thanks for pointing this out, and, evidently the reader will appreciate the explanation about the 25 nM sample sensitivity.
As far as I am concerned, all my question are solved and I think this is a fine manuscript that should be published.
